# The Efferocytosis‐Driven Macrophage Polarization: A Concerto in the Tumor Immunosuppressive Microenvironment

**DOI:** 10.1002/mco2.71038

**Published:** 2026-10-19

**Authors:** Qiuping Chen, Chunzhi Wu, Changquan Huang, Hang Zhou

**Affiliations:** ^1^ School of Basic Medical Sciences Chengdu University of Traditional Chinese Medicine Chengdu China; ^2^ Department of Gastroenterology The First Medical Center Chinese PLA General Hospital Beijing China; ^3^ Department of Oncology Chengdu Sixth People's Hospital Chengdu China; ^4^ West China School of Medicine Sichuan University Affiliated Chengdu Second People's Hospital Chengdu China

**Keywords:** efferocytosis, immunosuppression, macrophage polarization, tumor microenvironment

## Abstract

Efferocytosis plays a critical role in maintaining tissue homeostasis and controlling inflammation. However, in the tumor microenvironment, this process is hijacked to promote immune suppression. Treatments like radiotherapy and chemotherapy lead to widespread tumor cell death, which triggers efferocytosis and drives macrophages into an immunosuppressive state, allowing the tumor to evade the immune system and resist treatment. Nonetheless, the way efferocytic signals alter macrophage function and promote protumor effects during the recognition, engulfment, and digestion of apoptotic cells remains largely unclear. Therefore, this review thoroughly examines the influence of signaling molecules during the three stages of efferocytosis on macrophage phenotype and function. Subsequently, we explore the impact of polarized M2‐type macrophages on shaping the immunosuppressive tumor microenvironment and regulating the development of drug resistance. Additionally, we critically evaluate the current status of clinical translation of efferocytosis receptor‐related drugs and analyze the potential reasons for their synergistic or antagonistic interactions with chemotherapy and immunotherapy. This study provides a comprehensive understanding of the regulatory mechanisms of efferocytosis in macrophage polarization, enhancing knowledge of the fundamental mechanisms underlying tumor immune evasion and offering important guidance for the development of anticancer therapies targeting the efferocytosis process.

## Graphical abstract

Efferocytosis is a key process for macrophages to clear apoptotic cells, which undergoes the “ Find Me” chemotaxis signal stage, the “eat me” phagocytosis recognition stage and the “digest me” digestion stage in turn. During the aforementioned three stages, various signaling molecules released by apoptotic cells can promote the phenotypic polarization of macrophages, particularly toward M2 polarization. Polarized M2 macrophages promote tumor cell migration and invasion, angiogenesis, and immune regulation through direct and indirect pathways, thereby promoting tumor malignant progression. Moreover, while the therapeutic approach aimed at efferocytosis shows certain promise, its clinical application still faces several challenges, such as the complexity of synergistic effects, tumor type heterogeneity, limited effectiveness of certain targets, and possible cancer‐promoting consequences after phagocytosis. Future research should integrate emerging techniques like single‐cell omics, spatial omics, and nanotechnology to develop new tools that can monitor and regulate the efferocytosis state in the tumor microenvironment in real time, and explore combined treatment strategies based on precision medicine, with a view to providing a new breakthrough direction for tumor immunotherapy.

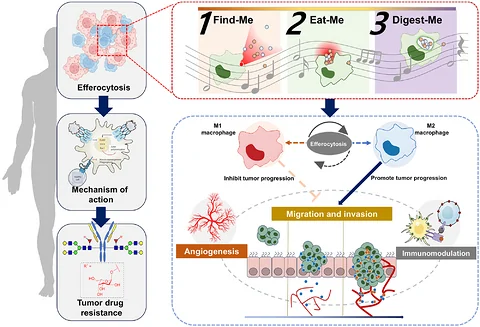

## 1 Introduction

The global increase in cancer‐related illnesses and fatalities poses a significant challenge to improving life expectancy worldwide [1, 2]. According to data from the International Agency for Research on Cancer, approximately 19.3 million new diagnoses and 10.2 million deaths occurred in 2020, with projections suggesting the global impact will rise by 47% to 28.4 million cases by 2040 [3]. At present, chemotherapy and radiotherapy serve as primary treatments for advanced‐stage malignancies [4], primarily functioning by triggering programmed cell death in cancerous tissue [5]. When chemotherapy kills tumor cells, several immune cells in the tumor microenvironment participate in the removal of apoptotic tumor cells through efferocytosis [6]. Efferocytosis involves a highly coordinated sequence in which phagocytic cells recognize, engulf, and digest apoptotic cells to avoid the onset of secondary necrosis and inflammatory responses [7]. This process is carried out by either professional phagocytes (like macrophages, neutrophils, and dendritic cells) or neighboring “nonprofessional” phagocytes (such as epithelial cells and fibroblasts). In particular, it is regulated by a set of precise signals: “find me” signals direct phagocytes to apoptotic cells, “eat me” signals facilitate their identification and ingestion, and “don't eat me” signals limit phagocytic activity when necessary to maintain tissue homeostasis [8]. Efferocytosis is essential for the removal of apoptotic cells, helping to maintain tissue balance and facilitate healing after injury or infection.

However, in the tumor microenvironment, this mechanism may be utilized by tumor cells to escape immune surveillance and facilitate tumor growth. Recent studies indicate that efferocytosis in the tumor microenvironment is a key pathological factor contributing to immunosuppression, tumor growth, and therapy resistance [9, 10, 11, 12]. The research by Cook et al. [13] demonstrated that the deletion of the efferocytosis receptor MerTK reduced tumor progression. Subsequent research also indicates that the process of efferocytosis can alter the polarization of macrophages [14, 15, 16, 17]. Although there has been notable advancement in efferocytosis research recently, the molecular mechanisms through which efferocytosis influences the shift of macrophages to a protumor phenotype remain a key unresolved question in this area. This research gap not only limits our understanding of tumor immune escape but also hinders the progress of targeted anticancer treatments.

In this review, we will provide a thorough exploration of how efferocytosis regulates macrophage polarization (Figure 1). This study explores the regulatory mechanisms of macrophage polarization influenced by signaling molecules during the three phases of efferocytosis: the “find me” phase, the “eat me” phase, and the digestion stage. Based on this, we conducted a further analysis of the current state of clinical translation in this area and systematically summarized the main challenges being faced, such as the complexity of synergistic effects, tumor type heterogeneity, limited efficacy of certain targets, and potential procancer effects following phagocytosis. At the same time, we pointed out emerging opportunities, including the identification of key immunosuppressive macrophage subsets, the development of nanodelivery platforms, and combinatorial therapeutic strategies guided by precision medicine. It should be highlighted that the regulatory mechanisms in this review are summarized via the M1/M2 classification. Even though this framework is a useful theoretical tool for understanding macrophage polarization and functional distinctions, it is unable to represent the extreme plasticity and heterogeneous nature of macrophages in the TME. Future studies should go beyond the fixed classification of M1/M2 phenotypes and investigate how efferocytosis influences the functional states of various macrophage subsets within the dynamic tumor microenvironment, emphasizing its varying effects on cellular metabolism, antigen presentation, and interactions with other immune cells. In summary, advancements in this research area could lead to new possibilities for tumor immunotherapy, providing significant scientific insights and clinical potential.

**FIGURE 1:**
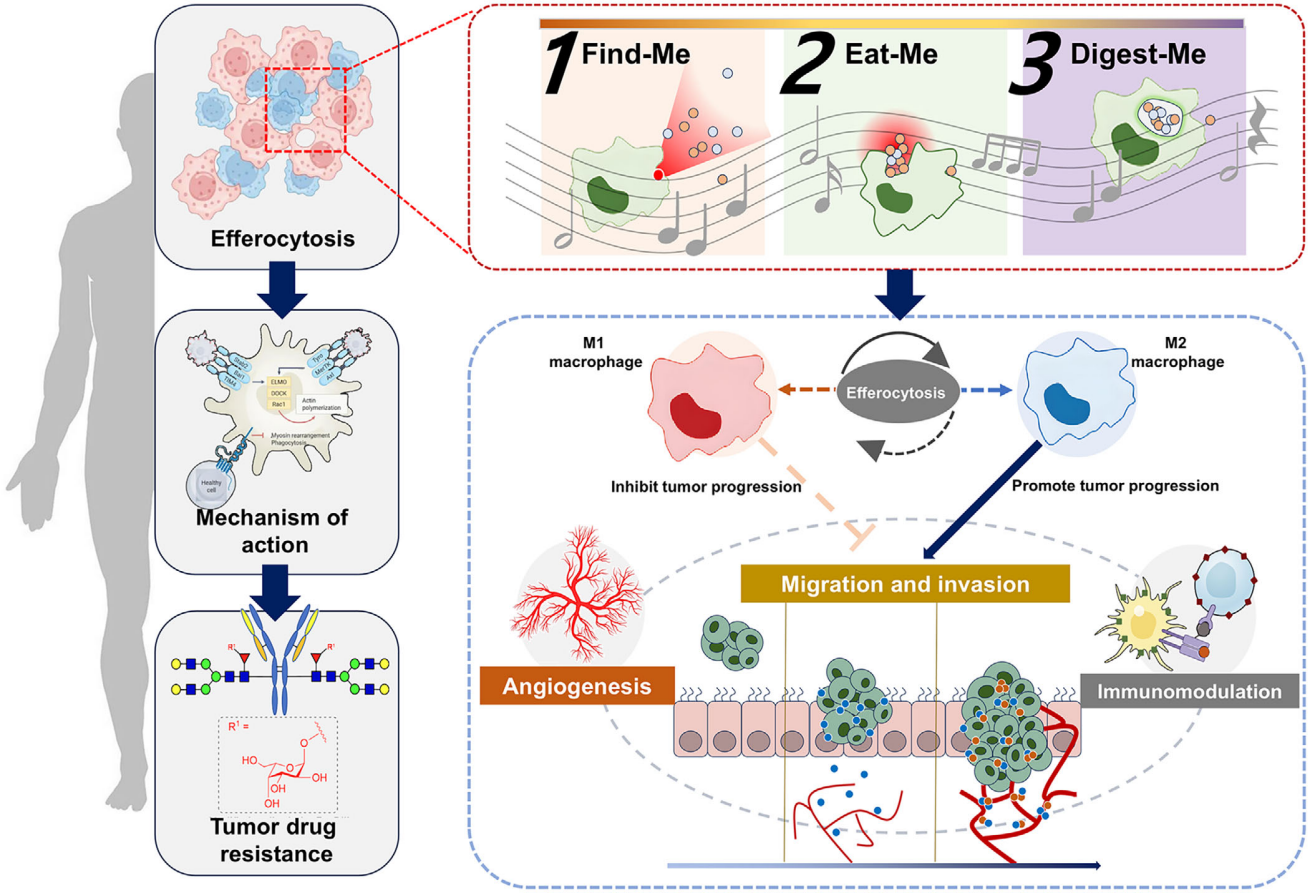
The effect of macrophage polarization mediated by efferocytosis on tumor cells. Apoptotic cells induce macrophage burial, which inhibits the polarization of M1 macrophages and promotes the polarization of M2 macrophages. Polarized M2 macrophages enhance tumor angiogenesis, tumor cell proliferation, metastasis, and immune escape by secreting specific cytokines. This multifaceted process eventually leads to the formation of a tumor immunosuppressive microenvironment and the generation of tumor resistance.

## 2 Macrophage Polarization Mediated by Efferocytosis

Apoptotic cells generate “find me,” “eat me,” and “digest me” signals that direct the efferocytosis process performed by phagocytic macrophages. This process not only facilitates the clearance of apoptotic cells but also plays a crucial role in regulating macrophage polarization. Efferocytosis in the tumor microenvironment enhances macrophage polarization to the M2 phenotype, modulates the release of anti‐inflammatory factors and proinflammatory cytokines, and influences the activation and maturation of immune cells, leading to a cascade of anti‐inflammatory and immunosuppressive signals. This process promotes tumor cells to evade immune surveillance mechanisms, leading to tumor progression. Therefore, this section will describe the regulatory mechanisms of key signaling molecules in macrophage polarization during the three stages of efferocytosis.

### 2.1 The Regulatory Role of the “Find Me” Phase in Macrophage Polarization

During the “find me” phase, cells undergoing apoptosis secrete several soluble signals, including nucleotides (ATP), sphingosine‐1‐phosphate (S1P), the C‐X3‐C motif chemokine ligand 1 (CX3CL1), lysophosphatidylcholine (LPC), and intercellular adhesion molecule 3 (ICAM3) [18]. These molecules not only help phagocytes identify and approach apoptotic cells but also significantly influence the polarization of macrophages, especially in fostering the development of immunosuppressive M2‐type macrophages. In this section, we summarize the regulatory mechanisms for macrophage polarization during the “find me” phase of efferocytosis (Figure 2).

**FIGURE 2:**
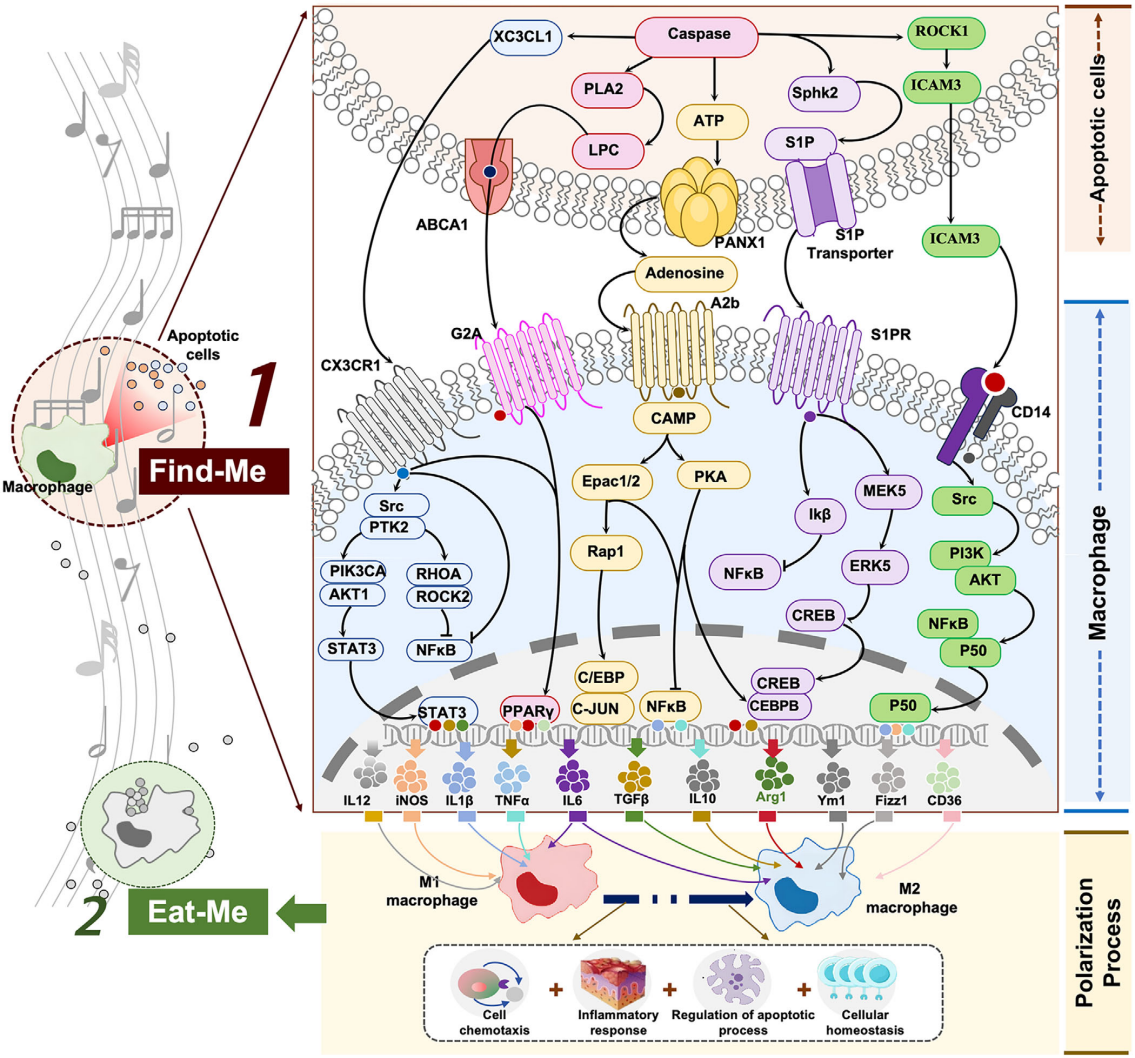
The mechanism of the “find me” phase in regulating macrophage polarization. During efferocytosis, apoptotic cells release a series of “find‐me” signals that recruit phagocytes to sites of cell clearance. Beyond facilitating apoptotic cell engulfment, these signals actively modulate macrophage polarization, thereby reshaping inflammatory and tumor microenvironments. Specifically, CX3CL1 primarily enhances the transcription of STAT3 and PPARγ, while inhibiting the transcriptional activity of NF‐κB. LPC directly enhances the transcription of PPARγ, while inhibiting the transcriptional activity of NF‐κB. ATP regulates the Epac1/2 and PKA pathways by converting into the signaling molecule cAMP. S1P, on the other hand, upregulates MEK5 to modulate the transcriptional activity of CREB while inhibiting the activation of NF‐κB. In contrast to these predominantly immunoregulatory signals, ICAM3 preferentially drives M1‐like macrophage polarization and proinflammatory cytokine expression through activation of p50‐dependent transcriptional programs. Collectively, the “find me” signal predominantly favors M2‐like macrophage polarization, thereby contributing to the establishment of an immunosuppressive microenvironment that supports tumor progression.

#### 2.1.1 Atp

ATP serves as a primary energy source, supplying essential energy for cellular functions. Research indicates that during apoptosis, dying cells release considerable ATP into the extracellular environment via the caspase‐activated pannexin‐1 channel [19]. Furthermore, ATP can be released from cells undergoing apoptosis via the secretion of exosomes or through the use of particular enzymes [20]. During efferocytosis, the ATP released from apoptotic cells in a caspase‐dependent manner is converted to adenosine on the surface of macrophages by 5′‐ectonucleotidase to trigger the adenosine receptor A2B [21, 22, 23]. Once activated, the A2B receptor associates with the Gs protein, which triggers an increase in intracellular cyclic adenosine monophosphate (cAMP) by activating adenylyl cyclase to catalyze the transformation of ATP into cAMP [24, 25]. It should be noted that this procedure triggers the “find me” signal release while also playing a vital role in the induction of macrophage polarization. Yet, how cAMP leads to macrophage polarization continues to be a subject of interest.

Many studies have shown that cAMP can influence macrophages to adopt an M2‐like phenotype, reducing the immune response and facilitating tumor immune evasion in the tumor microenvironment. In high‐grade serous ovarian cancer, the accumulation of cAMP in macrophages drives their polarization toward the M2 phenotype, resulting in immunosuppressive and tumor‐promoting effects [26]. This phenotype is characterized by diminished phagocytosis, lower levels of immune‐stimulating cytokines, and increased release of metastasis‐promoting molecules, including cytokines, growth factors, ECM components, proteases, and prostaglandin E2. In breast cancer, cAMP expression shifted as macrophages polarized, occurring mainly in the M2 phenotype [27]. In lung adenocarcinoma, cAMP facilitates the development of an immunosuppressive microenvironment and tumor progression by inducing M2 polarization through the PKA/CREB pathway [28]. Additionally, Serezani et al. [29] discovered that cAMP attaches to and triggers the regulatory subunit of PKA, which leads to the phosphorylation of the transcriptional activation site at serine 133 and the subsequent activation of the anti‐inflammatory transcription factor CREB. In glioblastoma multiforme, apoptotic tumor cells promote M2 macrophage polarization via the cAMP–CREB signaling pathway [30]. Ruffell and coworkers demonstrated that deletion of CREB‐binding sites from the Cebpb promoter blocked macrophage activation and reduced specific M2 genes, including Msr1, IL‐10, Il13ra, and Arg‐1 [31].

It is important to note that cAMP signaling is vital in modulating macrophage polarization by suppressing proinflammatory pathways in inflammatory diseases. In B. anthracis infection, the edema toxin released by the bacteria inhibits p65 phosphorylation at Ser536 via the cAMP/PKA pathway, thus reducing NF‐κB's transcriptional activity [32]. Moreover, the study by Tavares et al. [33] revealed that cAMP triggers the transcription of C/EBP and c‐Jun via the Epac/Rap1 axis and simultaneously restricts NF‐κB levels, playing a crucial bidirectional regulatory role in maintaining the homeostasis of M1 macrophages. It is well known that the activation of the NF‐κB signaling pathway has been widely recognized as a crucial mechanism for M1 polarization. The increased levels of its downstream proinflammatory factors, including TNF‐α, IL‐1β, and iNOS, establish the molecular basis of the classical inflammatory phenotype [34, 35]. Dai et al. [36] additionally demonstrated that blocking NF‐κB signaling could shift M1 macrophages to the M2 phenotype in a model of cardiac dysfunction.

In conclusion, cAMP facilitates the M2 polarization of macrophages in the tumor microenvironment by activating the core transcription factor CREB. M2‐type macrophages promote migration and new blood vessel formation through the synthesis of beneficial extracellular matrix (ECM)G2 accumulation receptor and vascular endothelial growth factor (VEGF) secretion, while also aiding immune evasion by attracting immunosuppressive cells via the release of proangiogenic factors and growth factors. Conversely, in noncancerous inflammatory conditions, cAMP mainly drives M1 macrophage polarization via signaling pathways like NF‐κB and c‐Jun, which efficiently regulate efferocytosis and accelerate the regression of inflammation.

#### 2.1.2 S1p

During apoptosis, the upregulation of sphingosine kinase 1 (SphK1) results in increased S1P release from dying cells. Concurrently, sphingosine kinase 2 (SphK2) is activated by Caspase‐1 cleaving its N‐terminus, leading to a substantial release of S1P outside the cell membrane [37, 38]. The primary way S1P functions is by interacting with S1P receptors located on the cell membrane. To date, five subtypes of S1P receptors have been identified: S1PR1, S1PR2, S1PR3, S1PR4, and S1PR5, with S1PR1–S1PR4 being commonly expressed in macrophages [39]. A variety of earlier investigations have established that S1P is expressed in different types of cancer, such as those of the breast, colon, and prostate, and is associated with the advancement of tumors, metastatic activity, and the development of an immunosuppressive niche [40, 41]. Nonetheless, the way in which S1P achieves such specificity is yet to be clarified.

In the tumor microenvironment, S1P released by dying cancer cells can promote macrophage polarization toward an M2‐like phenotype while suppressing the M1 phenotype, which is characterized by reduced proinflammatory factor secretion (TNFα, IL‐12) and increased anti‐inflammatory factor IL‐10 secretion [42]. In cocultures of macrophages and apoptotic cells, S1P promotes CREB phosphorylation through the MEK5/ERK5 pathway, leading to increased ARG2 expression and reduced NO production [43]. Similarly, in bone marrow‐derived macrophages, S1P regulates NF‐κB expression by inhibiting iNOS, while simultaneously increasing the expression of Arg1 [44]. Arg1 and Arg2 are essential indicators of M2‐type macrophages, significantly contributing to arginine metabolism and mitochondrial energy reprogramming during M2 polarization [45]. In glioblastoma, S1P prevents the polarization of M1 macrophages by hindering the activation of NF‐κB signaling through the reduction of p65 phosphorylation and IκBα degradation [46]. In a coculture of human primary macrophages and MCF‐7 breast cancer cells, S1P from apoptotic tumor cells influences macrophage phenotype by suppressing NF‐κB activation [42]. In B16 melanoma cells, inhibition of SPHK1‐dependent S1P production increased the number of M1 macrophages and promoted tumor growth [47]. Along with S1P derived from apoptotic cells, S1P can also be secreted by other cells in the tumor microenvironment when triggered by tumor cells. In an in vitro study in which macrophages were stimulated by conditioned medium from liver cancer cells and hepatic stellate cells (HSCs), tumor cells triggered the Nrf2/SphK1 pathway in HSCs to release S1P, which subsequently activated PPARα through S1PR4 and promoted M2 polarization in macrophages [48]. Notably, the regulatory role of S1P on M2 macrophage polarization is not only present in the tumor microenvironment but also exhibits a similar mechanism in inflammatory diseases. For example, in the atherosclerosis model, S1P facilitates IL‐4 interaction with IL‐4Rα/IL‐2Rγ via S1PR1 and S1PR2, leading to STAT6 activation and its nuclear translocation, which enhances SOCS1 expression [49]. It is well known that SOCS1 drives M2 polarization by increasing the ratio of Arg1/iNOS [50].

While extracellular S1P from apoptotic tumor cells mainly induces the release of anti‐inflammatory cytokines and promotes M2 macrophage polarization, intracellular S1P leads macrophages to display traits linked to proinflammatory cytokine production in a cancerous environment. For example, S1P1 signaling within macrophages associated with the lymphatics triggers both the expression of NLRP3 and the generation of IL‐1β in breast cancer [51]. Moreover, intracellular S1P activates NF‐κB signaling, resulting in the transcription of IL‐6 and TNF‐α, which enhances STAT3 expression, facilitating the transition from chronic inflammation to cancer [52]. Significantly, the seemingly paradoxical regulatory impact of S1P signaling on macrophage functions is largely contingent upon the compartmentalized distribution of S1P. More specifically, extracellular S1P primarily induces M2‐type polarization and immunosuppressive effects, while intracellular S1P tends to trigger an M1‐style proinflammatory reaction. This compartmentalization mechanism allows S1P to achieve functional diversity on the same molecular basis, thus adapting to various disease microenvironments. Consequently, upcoming studies ought to concentrate on clarifying how S1P's compartmentalized distribution is dynamically regulated within various tumor environments, creating instruments for precise interference with internal or external S1P signaling, and investigating integrated therapeutic approaches centered on targeting specific S1P compartments.

#### 2.1.3 Cx3cl1

In the tumor microenvironment, apoptotic cells release the “find me” signal CX3CL1 to recruit macrophages for phagocytosis. Further studies have shown that CX3CL1 upregulates the expression of the bridging molecule MFG‐E8 through the CX3CR1 receptor. MFG‐E8 enhances the macrophage‐mediated clearance of apoptotic cells through dual binding to phosphatidylserine (PS) on the targets and αvβ3/αvβ5 integrins on the phagocytes [53, 54]. The process also facilitates the polarization of macrophages into the M2‐type, shaping an immune‐suppressed environment that fosters tumor growth. In glioblastoma, CX3CL1 significantly upregulates the expression of the M2 macrophage marker PPAR‐γ, while downregulating the expression of the M1 macrophage marker IL1‐β [55]. In HCC, CX3CL1 facilitates macrophage M2 polarization through the activation of the Src/PTK2 signaling pathway and the enhancement of PIK3CA/AKT1 and RHOA/ROCK2 signaling, characterized by a significant increase in the M2 markers MRC1 and CD163 [56]. Moreover, in vitro studies show that CX3CL1 released by apoptotic cells interacts with the CX3CR1 receptor, activating the classical JAK2/STAT3 signaling pathway and promoting macrophage polarization toward the M2 phenotype [57]. Deficiency of CX3CR1 leads to increased infiltration of inflammatory monocytes/macrophages and enhances the proinflammatory M1‐like phenotype of macrophages. Generally, CX3CL1 from apoptotic cells promotes efferocytosis and M2 polarization in the tumor microenvironment, facilitating tumor immune evasion and metastasis.

In the inflammatory microenvironment, CX3CL1 significantly regulates macrophage polarization in a bidirectional and disease‐dependent manner. In a mouse model of intervertebral disc degeneration, apoptotic cells release CX3CL1, which activates the STAT3 pathway through CX3CR1, enhancing the expression of M2 markers IL‐10 and Arg‐1 [57]. However, in ankylosing spondylitis, CX3CL1 facilitates M1 polarization through the NF‐κB pathway, and inhibiting this pathway can prevent CX3CL1‐induced M1 polarization [58]. In summary, these evidence demonstrate that CX3CL1 is capable of exerting opposite transcriptional influences on macrophage polarization in different contexts. This difference stems from variations in the disease microenvironment that alter the signaling pathways, resulting in contrasting tissue‐specific effects of CX3CL1. In intervertebral disc degeneration, the tissue often shows a coexistence of chronic low‐grade inflammation and repair, along with increased STAT3 activity in the microenvironment. CX3CL1 mainly promotes M2 macrophage polarization through the STAT3 pathway, leading to anti‐inflammatory and tissue‐repair outcomes. However, in ankylosing spondylitis, chronic inflammation leads to sustained activation of the immune system, with the NF‐κB pathway prevailing in the microenvironment. In most disease contexts, the bidirectional adjustment of macrophage function is essential to achieving tissue homeostasis and resolving inflammatory issues.

#### 2.1.4 Lpc

In the early stages of apoptosis, Caspase‐3 activates calcium‐independent phospholipase A2 in apoptotic cells, leading to the hydrolysis of phosphatidylcholine (PC) in the cell membrane, resulting in the generation and extracellular release of LPC. By activating the G2 accumulation receptor (G2A)Phosphatidylserine on the surface of macrophages, LPC induces these cells to migrate and recognize targets, completing the initial recruitment step in efferocytosis [59, 60]. LPC serves as a primary lipid regulator once recruited, using multifaceted mechanisms to further adjust how macrophages polarize. It is worth noting that various research efforts have shown a substantial reduction in LPC concentrations within the majority of tumor microenvironments. For example, in breast cancer, the levels of LPC acyltransferase 1, a crucial enzyme for PC biosynthesis, are markedly increased and associated with early recurrence and unfavorable prognosis [61]. In solid cancer tissues, LPC levels are notably lower, whereas PC levels are higher [62]. Lower levels of LPC within the tumor's surroundings diminish its capacity to trigger M1 polarization; consequently, macrophages become more vulnerable to immunosuppressive cues, leading to M2 polarization and an overall environment that supports cancer development. Interestingly, the remaining low concentration of LPC may still play a role in promoting the polarization of tumor‐associated macrophages toward the M2 state through PPARγ. For example, in the tumor microenvironment, LPC facilitates macrophage polarization toward the M2 phenotype, exhibiting anti‐inflammatory and protumor properties through PPARγ activation, accompanied by significant upregulation of markers such as Arg‐1, MR1, CHI3L3, IL‐10, and TGF‐β [63]. In melanoma, LPC promotes M2‐type macrophage polarization by binding to the G protein‐coupled receptor G2A on the surface of macrophages, whereas reducing LPC production or G2A activity can enhance antitumor immunity and suppress metastasis [64]. In conclusion, while LPC levels are usually downregulated in the tumor microenvironment, remaining LPC can still promote macrophage polarization toward the M2 phenotype via the G2A or PPARγ signaling pathway.

It is important to note that elevated levels of LPC in the inflammatory microenvironment are linked to M1 macrophage polarization. Research indicates that LPC can promote the release of proinflammatory cytokines like COX‐2, TNF‐α, and IL‐6 by activating the Toll‐like receptor signaling pathways [65, 66]. Moreover, high levels of LPC can facilitate the differentiation of M1‐type macrophages by binding to the G2A receptor, increasing their secretion of proinflammatory cytokines like IL‐1β, IL‐6, and TNF‐α [66]. Inhibition of G2A receptor expression on macrophages using siRNA markedly reduces LPC‐induced proinflammatory cytokine production. In contrast, other research has suggested that exogenous LPC enhances the polarization of M2 macrophages through PPARγ activation [67, 68]. These differences likely stem from the difference in LPC concentration. It is probable that these variations result from differing LPC concentrations. While in vitro experiments often use low doses of external LPC to reflect normal physiological conditions, endogenous LPC levels become much higher in an inflammatory environment.

In conclusion, the influence of LPC on macrophage phenotypes varies, primarily depending on the LPC concentration in the surrounding environment. In the tumor microenvironment, residual low levels of LPC mainly promote M2 polarization via mechanisms like PPARγ, forming an immunosuppressive environment and facilitating tumor growth. In the inflammatory microenvironment, elevated levels of LPC mainly promote M1 polarization via pathways like TLR/G2A, intensifying the inflammatory response and promoting immune cell infiltration.

#### 2.1.5 Icam3

During efferocytosis, apoptotic cells recruit macrophages by emitting microparticles containing ICAM3 as a “find me” signal, while remaining ICAM3 on the cell surface triggers phagocytosis and clearance as an “eat me” signal [69]. Besides acting as a recognition signal for apoptotic cells during efferocytosis, the role of ICAM3 in macrophage polarization is gaining increasing interest. In contrast to efferocytosis signals that serve as temporary intercellular communication, the latter plays a more crucial role in shaping the tumor immune microenvironment. In non‐small cell lung cancer (NSCLC), ICAM3 expression levels in tumor tissues are significantly elevated compared with adjacent tissues, and it has been recognized as a marker for the M1 macrophage subpopulation [70]. In research on targeted therapy for colorectal cancer, the level of ICAM3 protein in M1 macrophage‐derived biomimetic nanovesicles (M1‐LNVs) was found to be significantly higher than in unpolarized monocytes [71]. It was further identified by Shen et al. [72] that ICAM3's recruitment of Src kinase through the YLPL motif activates the PI3K–AKT signaling pathway, encouraging NF‐κB (p50) activation. It is noteworthy that the ICAM3 promoter region can be bound by activated p50, establishing a positive feedback cycle that promotes the polarization of M1 macrophages. In vitro studies additionally demonstrated that M1‐type macrophages exhibited higher levels of ICAM3 expression than M2‐type macrophages, and that the downregulation of ICAM3 was inversely related to the upregulation of RUNX3 expression [73]. These results indicate that RUNX family transcription factors might play a role in regulating ICAM3 expression. Conversely, ICAM3‐negative macrophages exhibit stronger M2 polarization features, with high expression of M2 markers such as CD163 and CD206 [74]. Hashimoto et al. used a drug to upregulate the transcription factor C/EBPα, which significantly reduced the number of M2‐type TAMs in advanced hepatocellular carcinoma, accompanied by a significant upregulation of ICAM3 at both the gene and protein levels [75].

In conclusion, while ICAM3 has been identified as a signal originating from apoptotic cells during efferocytosis, the potential to manipulate ICAM3 for reshaping the tumor immunosuppressive microenvironment predominantly relies on its regulation of macrophage polarization. Consequently, focusing on the ICAM3 molecule involved in efferocytosis might offer a way to influence the polarization of tumor‐associated macrophages and strengthen the immune system's reaction against tumors.

### 2.2 The Regulatory Role of the “Eat Me” Phase in Macrophage Polarization

Currently, the “eat me” signals are broadly classified into phosphatidylserine (PS)‐related and non‐PS signals. Among these, PS is the best‐characterized “eat me” signal exposed on the surface of apoptotic cells and is crucial for phagocytic recognition and engulfment. During apoptosis, XK‐related protein 8 is activated via caspase‐mediated cleavage, which disrupts phospholipid asymmetry within the plasma membrane and promotes the externalization of PS from the inner to the outer membrane leaflet [76, 77]. PS subsequently regulates efferocytosis and macrophage polarization primarily through the activation of PS receptors (PSRs), with specific ones like brain angiogenesis inhibitor 1 (BAI1), T‐cell immunoglobulin and mucin domain‐containing protein 4 (Tim‐4), and Stabilin‐2 directly interacting with PS [78]. In contrast, the interaction of PS with Tyro3‐Axl‐Mer (TAM) family receptors (Tyro3, AXL, and MERTK) and alpha‐v beta‐5 (αvβ5) integrins is mediated by Growth arrest‐specific protein 6 (Gas6) and Protein S (ProS) acting as bridging molecules. Apart from PS, researchers have discovered various other “eat me” indicators, such as Annexin A1(ANXA1), calreticulin (CRT), and thrombospondin‐1 (TSP‐1) [79]. This section concentrates on the key “eat me” signals responsible for regulating macrophage polarization, as shown in Figure 3.

**FIGURE 3:**
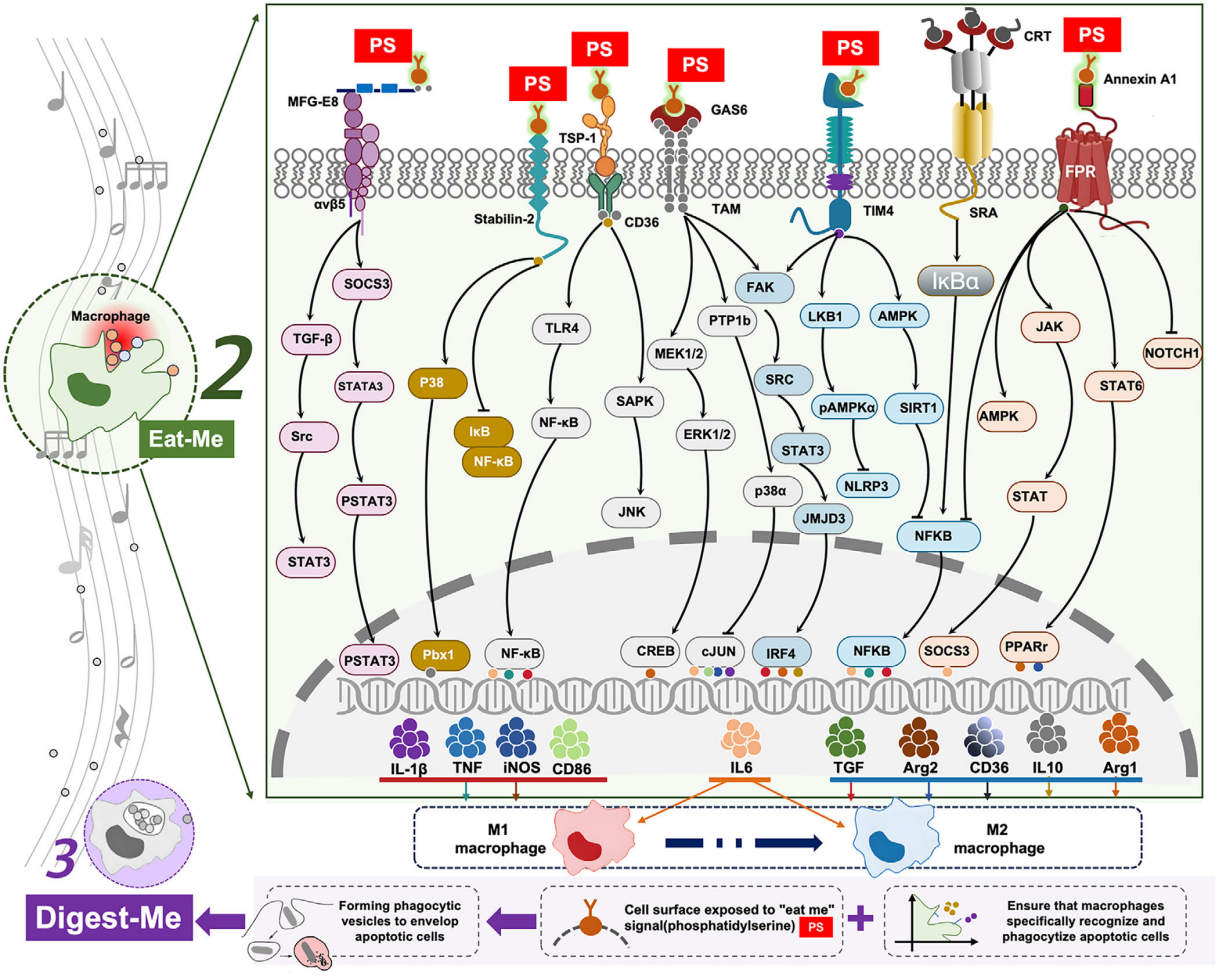
The regulatory effect of the “eat me” signal on macrophage polarization. Arrows (→) indicate promotion, activation, or positive regulation, while T‐shaped bars (—|) indicate inhibition or blockade. Apoptotic cells interact with macrophage surface receptors directly or indirectly through externalized PS, thereby regulating macrophage polarization. αvβ5 integrin recognizes PS in an MFG‐E8‐dependent manner and promotes STAT3 activation through upregulation of TGF‐β and SOCS3 expression. Stabilin‐2 directly binds PS and suppresses NF‐κB activation via increased Pbx1 transcription. The TAM receptor family recognizes PS through the bridging molecule GAS6, leading to increased CREB and IRF4 expression and reduced c‐JUN transcription. Annexin A1 acts as another bridging molecule between PS and FPR, activating AMPK signaling, enhancing SOCS3 and PPARγ transcription, and suppressing the NOTCH1 and NF‐κB pathways. These signaling pathways associated with apoptotic cell clearance generally promote M2 polarization. By contrast, TSP‐1 and CRT primarily promote M1 polarization. TSP‐1 bridges CD36 and PS to activate NF‐κB and JNK signaling, whereas CRT promotes M1 polarization through NF‐κB activation.

#### 2.2.1 PS‐Related Receptors

##### 2.2.1.1 TAM Receptors

In the tumor microenvironment, radiotherapy and chemotherapy produce a significant number of apoptotic tumor cells, whose surfaces evert to reveal the “eat me” signal PS. Gas6 and ProS, the ligands of the TAM receptor, can bind to PS via their γ‐carboxyglutamic acid‐rich domains while also activating TAM receptors (Tyro3, Axl, and MerTK) on macrophages through their other ends. This process not only facilitates the clearance of apoptotic cells by macrophages but also alters the tumor immunosuppressive microenvironment by modulating macrophage polarization. Numerous studies have shown that M1‐like macrophages express significantly lower levels of TAM receptors than M2‐like macrophages [80, 81, 82, 83]. For example, in M‐CSF‐polarized and dexamethasone‐treated M2‐like human monocyte‐derived macrophages, AXL mRNA levels are higher compared with M1‐like macrophages [82, 83]. Findings from Shibata et al. [84] indicate that the suppression of Axl leads to a marked drop in Arg1 within macrophages undergoing M2 differentiation, alongside a rise in M1 macrophage levels. Similarly, MerTK expression is higher in M2‐like macrophages than in either unstimulated or M1‐polarized macrophages. For example, in classical Hodgkin lymphoma, there is a rise in M2 macrophage levels along with a higher expression of the MerTK gene [85]. In murine bone marrow‐derived macrophages, levels of Mertk mRNA are elevated in the M2‐like phenotype stimulated by IL‐4/IL‐13 compared with the M1‐like phenotype induced by LPS/IFN‐γ [80]. In human monocyte‐derived macrophages, MerTK protein levels increase after stimulation with IL‐4 and IL‐10 compared with unstimulated or LPS‐treated cells [86, 87].

The TAM receptor family induces immunosuppression in the tumor microenvironment by downregulating M1 activation‐related genes and facilitating macrophage polarization toward a protumor phenotype. Many studies have shown that activating TAM receptors greatly decreases M1 marker expression like TNF‐α, IL‐1β, IL‐6, and CD86, while increasing M2 marker expression such as CD206, IL‐10, Arg1, and Arg2 [88, 89, 90, 91]. In colorectal cancer, apoptotic cells activate MERTK and AXL receptors on the macrophage surface via PS, upregulating the STAB1 protein and promoting M2 polarization characterized by increased Arg1 and CD206 expression [92]. The activated Axl/MerTK receptor induces GSK3β phosphorylation and inhibits NF‐κB nuclear translocation through the PI3K/Akt pathway. At the same time, Gas6‐mediated signaling enhances STAT6 activation, both of which together promote M2 polarization [93]. Furthermore, activated TAM receptors promote SOCS1/3 expression via the IFNAR–STAT1 signaling pathway, inhibit TLR pathway signal transduction, and facilitate macrophage polarization toward the M2 phenotype [94]. In the coculture system of apoptotic tumor cells and macrophages, PS from apoptotic cells activates the MERTK–FAK–SRC–STAT3–JMJD3 pathway, leading to Irf4 activation and decreased H3K27me3 levels. This process enhances the expression of M2 macrophage marker genes, including Arg1, Mgl1, TGF‐β, CD206, and IL‐10 [95]. These results further indicate that TAM receptors promote the M2 polarization of macrophages while inhibiting M1 polarization. Conversely, inhibiting TAM receptors through drugs curtails M2 polarization and brings back M1‐type inflammatory programs [96, 97].

In addition to TAM receptors, their ligands Gas6 and ProS also play regulatory roles in macrophage polarization. In acute myeloid leukemia (AML), Gas6 significantly promotes the phosphorylation of proteins including ERK1/2, CREB, p38, MSK1, ATF1, AKT, and STAT6 [98]. Significantly, research has verified that M2 macrophage polarization is intimately connected to the activation of the aforementioned proteins. For example, ATF1 binds to the promoter regions of the HO‐1 and LXRβ genes, increases LXR‐dependent cholesterol efflux, and facilitates M2 macrophage polarization [99, 100]. In tumor‐derived macrophages, ProS suppresses M1 polarization triggered by LPS and IFN‐γ via the Mer/Tyro3/PTP1b/p38α/cJun signaling pathway, leading to decreased expression of IL‐1β, IL‐6, CD86, and TNF‐α [101]. Meanwhile, the binding of Gas6/ProS ligands to MerTK induces receptor dimerization and autophosphorylation, which inhibits the NF‐κB pathway and increases PD‐L1 levels via the p38/STAT3 pathway. This process inhibits M1 polarization and maintains the M2 phenotype. In mouse bone marrow‐derived macrophages, Gas6 and ProS activate TAM receptors in an autocrine manner, effectively reducing the release of proinflammatory cytokines, including TNF‐α, IL‐6, and IL‐1β [88]. Additionally, Gas6 activates the PI3K/Akt/STAT1/LXR/Arg2 signaling pathway via MerTK, prompting the polarization of M2‐like macrophages, which shows increased levels of Gas6.

In summary, during efferocytosis, TAM receptors and their ligands GAS6 and PROS1 promote M2 macrophage polarization and inhibit M1 polarization by activating multiple downstream signaling pathways. This process transforms the tumor immune microenvironment into a state that suppresses immunity, promoting tumor growth and resistance to therapy. These results indicate that inhibiting TAM receptor signaling during endocytosis may effectively address tumor‐induced immunosuppression and improve antitumor immunity.

##### 2.2.1.2 TIM4

As a PSR on macrophages, TIM4 is responsible for the specific recognition of PS on apoptotic tumor cell membranes [102]. Following chemotherapy‐induced apoptosis of tumor cells, macrophages increase TIM4 expression, leading to Rac1 signaling activation via the LRP1/GULP pathway, which facilitates cytoskeletal remodeling and phagocytic cup formation during efferocytosis [59]. Inhibition of TIM4 on macrophages with antibodies enhances the STING‐mediated Type I interferon response in tumor‐associated macrophages and decreases their ability to clear apoptotic cells [103, 104]. Notably, excessive efferocytosis in the tumor microenvironment may lead to the breakdown of tumor antigens, hindering macrophage antigen presentation and restricting CD8+ cytotoxic T cell activation. This process also shifts macrophages toward an immunosuppressive M2‐like phenotype, ultimately diminishing antitumor immunity. M2‐polarized macrophages exhibit significantly higher Tim4 expression, compared with M1‐polarized macrophages [105]. In ovarian cancer, TIM4+ tumor‐associated macrophages show elevated levels of oxidative phosphorylation (OXPHOS) and Arg1 expression [106]. These macrophages also show increased mitochondrial fission, which may lead to lower oxidative stress. Additionally, TIM4 promotes metabolic reprogramming through lysosomal activation, reinforcing the metabolic characteristics of M2 macrophages. These results emphasize the key role of TIM4 in establishing an immunosuppressive macrophage environment and facilitating peritoneal metastasis in ovarian cancer. Liang et al. [95] discovered that within the tumor microenvironment, the PS released from apoptotic tumor cells binds to the surface of macrophages through TIM4, leading to the activation of the FAK–SRC–STAT3 pathway. This ultimately results in an increase in JMJD3 and IRF4 expression, promoting the polarization of M2 macrophages. Anti‐TIM4 antibody effectively inhibited the activation of FAK and STAT3, blocking the polarization of M2‐like macrophages and tumor progression. The findings indicate a significant connection between TIM4 signaling and the polarization of M2 macrophages in the tumor microenvironment.

Unlike its function in tumors, TIM4 regulates macrophage polarization in inflammatory settings mainly by inhibiting M1‐like polarization. In BMDMs from Tim‐4 knockout mice, lipopolysaccharide stimulation significantly increased the phosphorylation of IKKα/β, IκBα, and NF‐κB p65, accompanied by an upregulation of M1‐related genes like TNF‐α, IL‐1β, and iNOS [107]. In a septic shock mouse model, Tim‐4 significantly reduced the levels of iNOS, IL‐6, and TNF‐α by inhibiting NF‐κB activation [108]. Ding et al. [107] utilized the NF‐κB pathway inhibitor BAY 11–7082, which decreased the percentage of M1 macrophages, indicating that Tim‐4 primarily restricts M1 polarization by blocking the NF‐κB signaling pathway.

In summary, the way by which TIM4 directs macrophage polarization shows distinct differences between tumor‐associated and inflammatory microenvironments. In the tumor microenvironment, TIM4 promotes M2‐like macrophage polarization through pathways such as OXPHOS, lysosomal activation, and metabolic reprogramming, ultimately leading to tumor immunosuppression. However, in the inflammatory microenvironment, TIM4 primarily limits M1‐like macrophage polarization by downregulating the NF‐κB inflammatory pathway, promoting inflammation resolution and maintaining tissue homeostasis. Although progress has been made, the molecular mechanisms behind TIM4's functional switching in different microenvironments are still not well understood and require more research.

##### 2.2.1.3 αvβ5

αvβ5 integrin is a key receptor on the surface of macrophages and acts as a bridging molecule during the “find me” and “eat me” phases of efferocytosis. Research indicates that it does not directly bind to apoptotic cells, but facilitates the clearance of PS‐expressing apoptotic cells mainly by binding to the RGD motif of MFG‐E8 [109]. Crucially, αvβ5‐mediated endocytosis facilitates not only the clearance of apoptotic cells in tissues but also the polarization of M2 macrophages. In gastric cancer, the excessive expression of ITGB5, the β subunit forming αvβ5, shows a strong positive correlation with the infiltration of M2 macrophages, which implies a connection between αvβ5 levels and M2 polarization [110]. Zhang et al. [111] further confirmed through in vitro studies that αvβ5 integrin promotes macrophage polarization toward the M2 phenotype by enhancing PPARγ transcription, along with upregulation of IL‐10 expression. In prostate cancer, efferocytosis mediated by αvβ5/MFG‐E8 activates STAT3 to promote M2 macrophage polarization through the suppression of SOCS3 expression, as demonstrated by the up‐regulation of CD206, Ym‐1, IL‐10, TGF‐β, arginase, IL‐6, and CCL2 [112]. Notably, αvβ5 can further regulate macrophage polarization by responding to cytokines secreted by tumor cells or macrophages. In gliomas, αvβ5 on M2‐type TAMs facilitates autocrine TGF‐β‐induced activation of the Src/STAT3 pathway, promoting glioma stem cell maintenance and tumor growth [113]. Moreover, osteopontin secreted by glioma stem cells also maintains the M2 gene signature and phenotype of macrophages by binding to the αvβ5 integrin receptor [114]. While the previously mentioned evidence indicates that αvβ5 mainly facilitates M2 macrophage polarization via pathways like STAT3 and PPARγ, current research on the direct regulatory mechanisms is still quite limited. In particular, the functional heterogeneity of αvβ5 in different tumors requires more thorough exploration.

##### 2.2.1.4 Stabilin‐2

Stabilin‐2 is a receptor located on the surface of macrophages that directly recognizes PS. It is composed of repetitive extracellular fasciclin and EGF‐like domains, along with a single C‐type lectin‐like X‐link domain located near its transmembrane region [115]. After recognizing apoptotic cells, Stabilin‐2 activates Rac1 through intracellular Gulp1 and works with integrin αvβ5 to facilitate the cytoskeletal changes necessary for efferocytosis [18]. Efferocytosis mediated by Stabilin‐2 has also been associated with macrophage polarization toward an M2‐like phenotype [116]. Park et al. [117] found that the combination of Stabilin‐2 and PS on the surface of apoptotic cells stimulates macrophages to release the anti‐inflammatory cytokine TGF‐β. Moreover, in the coculture system of apoptotic cells and macrophages, Stabilin‐2 enhanced the expression of the Pbx1 transcription factor via the p38 MAPK signaling pathway, leading to increased IL‐10 expression [118]. This signaling pathway also inhibits M1 polarization by blocking IκB and NF‐κB phosphorylation.

In the tumor microenvironment, TGF‐β and IL‐10 jointly induce the polarization of macrophages toward the M2 phenotype, which possesses immunosuppressive and protumorigenic properties, and in turn, these macrophages synthesize and secrete more TGF‐β and IL‐10 [119]. Overall, the existing evidence connecting Stabilin‐2 to M2 macrophage polarization is still sparse and mostly indirect. Existing studies suggest that Stabilin‐2 primarily regulates macrophage immunosuppressive functions by enhancing efferocytosis and promoting TGF‐β and IL‐10 secretion. Nonetheless, it remains uncertain whether Stabilin‐2 directly induces M2 polarization in vivo. Notably, current findings mainly stem from basic experimental studies, and the underlying regulatory mechanisms remain to be fully clarified. Additional studies in tumor animal models and clinical samples will be necessary to clarify the specific function of Stabilin‐2 in macrophage polarization and tumor immune regulation.

#### 2.2.2 Non‐PS Signals

##### 2.2.2.1 ANXA1

ANXA1 is a member of the annexin superfamily, with a molecular weight of 37 kDa [120]. During efferocytosis, the activation of caspases and the release of intracellular calcium ions promote the recruitment of ANXA1 to the surface of apoptotic cells that are rich in PS. ANXA1 binds to exposed PS and facilitates the recognition and clearance of apoptotic cells by macrophages [121]. It is noteworthy that ANXA1, as a key immune regulatory protein, plays an important role in regulating macrophage polarization and reshaping the tumor immune microenvironment [122]. Growing evidence indicates that high ANXA1 levels in the tumor microenvironment correlate with increased M2 macrophage infiltration and immunosuppressive changes [123]. ANXA1 facilitates the transition of macrophages to the M2 phenotype by utilizing its receptor, FPR2, whether it operates as an intracellular signaling component or a secreted factor [124, 125]. Later research established that the ANXA1–FPR2 pathway promotes M2 polarization by synchronizing the activation of AMPK‐related signaling mechanisms [126]. In hepatocellular carcinoma, the N‐terminal domain of ANXA1 binds to FPR2, leading to enhanced macrophage infiltration and M2 polarization by inhibiting NF‐κB and NOTCH1 signaling pathways while simultaneously activating protumorigenic pathways such as JAK/STAT, AKT, and ERK [127]. Moreover, both intracellular and secreted ANXA1 have been demonstrated to enhance PPARγ and modulate CD36 expression through the activation of the FPR2/STAT6 signaling pathway [128]. In the tumor microenvironment, CD36 contributes to M2 macrophage polarization by binding to fatty acids released from tumor cells [129]. In opposition, ANXA1 knockdown significantly increases the expression of M1‐related markers like IL‐1β, IL‐6, and iNOS, while suppressing M2‐related genes such as Arg1, IL‐10, and Ym1 [130]. Recent research further suggests that the immunomodulatory function of ANXA1 may vary significantly depending on the stage of tumorigenesis [131]. In precancerous lesions, ANXA1 downregulation activates NF‐κB/MAPK‐mediated glycolytic reprogramming and promotes M1 polarization. Yet, in the course of malignancy, the overexpression of ANXA1 stimulates M2 macrophage polarization and cancer advancement by activating signaling related to AKT and ERK [131]. Together, these results establish ANXA1 as a key regulator of macrophage adaptability in the tumor microenvironment and indicate that targeted modulation of ANXA1 at different stages may offer a viable immunotherapeutic approach. Boosting ANXA1 function in early cancer stages may enhance M1‐driven antitumor immunity, while blocking ANXA1 signaling in late‐stage tumors could alleviate M2‐induced immunosuppression.

In conclusion, ANXA1 inhibits M1 polarization mainly by blocking inflammatory signaling pathways such as NF‐κB, NOTCH1, and IL‐6. At the same time, it facilitates M2 macrophage polarization via the activation of JAK/STAT, AKT/ERK, and STAT6‐related signaling pathways, thus creating an immunosuppressive tumor microenvironment that aids tumor progression.

##### 2.2.2.2 CRT

CRT is a 46‐kDa protein located within the endoplasmic reticulum lumen that performs various biological roles, such as acting as a molecular chaperone, acting as a molecular chaperone, regulating intracellular calcium levels, and contributing to cellular apoptosis. CRT forms a bridging complex with C1q and low‐density lipoprotein receptor‐related protein 1 (LRP1 or CD91) expressed on phagocytes to facilitate phagocytosis and clearance of apoptotic cells [132, 133]. Beyond acting as an “eat me” signal for clearing apoptotic cells, CRT significantly impacts the function and polarization of macrophages through signal transduction mechanisms. For instance, in 4T1 breast cancer cells, exposure to CRT on apoptotic cells caused 11.4% of macrophages to undergo M1 polarization, while γ‐Fe administration lowered this rate to 3.7% [134]. Duo et al. [135] showed that macrophages identify and internalize CRT through the surface receptor SRA, activating the IκBα/NF‐κB pathway, which results in the expression of M1‐associated factors (TNF‐α and IL‐6). Interestingly, Osman et al. [136] found that CRT‐exposed monocyte‐derived macrophages exhibit a distinct polarization state, notably characterized by the downregulation of Class II MHC molecules—an observation that questions the traditional M1/M2 classification. Research on CRT and macrophage polarization is currently limited, necessitating further investigation into its mechanisms and potential applications in the tumor microenvironment to enhance our understanding of its role in tumor biology and therapeutic modulation.

##### 2.2.2.3 TSP‐1

During efferocytosis, TSP‐1 functions as a bridging molecule linking PS on apoptotic cells to phagocytic receptors CD36 or CD47 on macrophages, facilitating apoptotic cell recognition and engulfment [18]. Accumulating evidence indicates that TSP‐1 primarily facilitates M1‐type macrophage polarization, which stands in opposition to other pathways mediated by efferocytosis signals. In oral squamous cell carcinoma, TSP‐1 upregulates the transcriptional levels of M1‐associated cytokines (TNFα, IL‐1β, IL‐6) by activating the p38 MAPK, Akt, and SAPK/JNK signaling pathways [137]. In a mouse model of obesity induced by a high‐fat diet, TSP‐1 promotes the formation of M1 macrophages through the activation of the CD36/TLR4/NF‐κB signaling pathway [138]. Conversely, in a model of lung injury and inflammation triggered by LPS, the binding of TSP‐1 to CD36 facilitates the synthesis of IL‐10, which is a hallmark of macrophages of the M2 phenotype [139]. This indicates that the impact of TSP‐1 on macrophage polarization may differ based on the specific disease model or microenvironment, even when the same receptor is involved. Notably, an in vitro study revealed that physiological concentrations of TSP‐1, upon binding to CD47 through its C‐terminal domain, led to the dissociation of CD47 from CD14, thus inhibiting the transcription of M1 macrophage‐related genes NF‐κB and AP‐1 [140]. These varying effects may indicate that regulation is dependent on concentration and specific microenvironments, where elevated local levels of TSP‐1 in inflammatory or tumor microenvironments promote M1 polarization, while lower external concentrations inhibit M1‐related gene expression. Nonetheless, the function of TSP‐1 in influencing macrophage polarization in the tumor microenvironment is still not fully understood. Future studies should focus on elucidating the specific molecular pathways by which TSP‐1 affects macrophage polarization in various microenvironments, particularly regarding its different roles in distinct tumor environments.

### 2.3 The Regulatory Role of the Digestion Phase in Macrophage Polarization

When macrophages engulf apoptotic cells, a series of intracellular events is triggered. Inside the lysosome, numerous enzymes activate to break down the engulfed apoptotic cells and release a variety of metabolites. These metabolites consist of amino acids, lipids, cholesterol, carbohydrates, nucleic acids, and fat‐soluble vitamins [141, 142]. These metabolites not only serve as degradation by‐products but also have the capacity to regulate macrophage phenotypes [143]. Recently, the expanding field of immunology has seen increased interest in how these metabolites influence macrophage polarization. Recent findings have shed light on the complex relationship between metabolites from apoptotic cells and macrophage polarization, bringing this topic to the forefront of immunological studies. This part of the paper provides an extensive overview of existing studies, focusing on the impact of apoptotic cell metabolites on the polarization of macrophages.

#### 2.3.1 Amino Acid Metabolism

In the tumor microenvironment, metabolites from apoptotic cells promote sustained efferocytosis through amino acid metabolism. Simultaneously, efferocytosis also reshapes macrophage metabolism, especially the arginine and tryptophan pathways. This process not only promotes macrophage polarization toward an immunosuppressive M2‐like phenotype but also aids in creating an immunosuppressive tumor microenvironment, ultimately promoting tumor initiation and progression (Figure 4).

**FIGURE 4:**
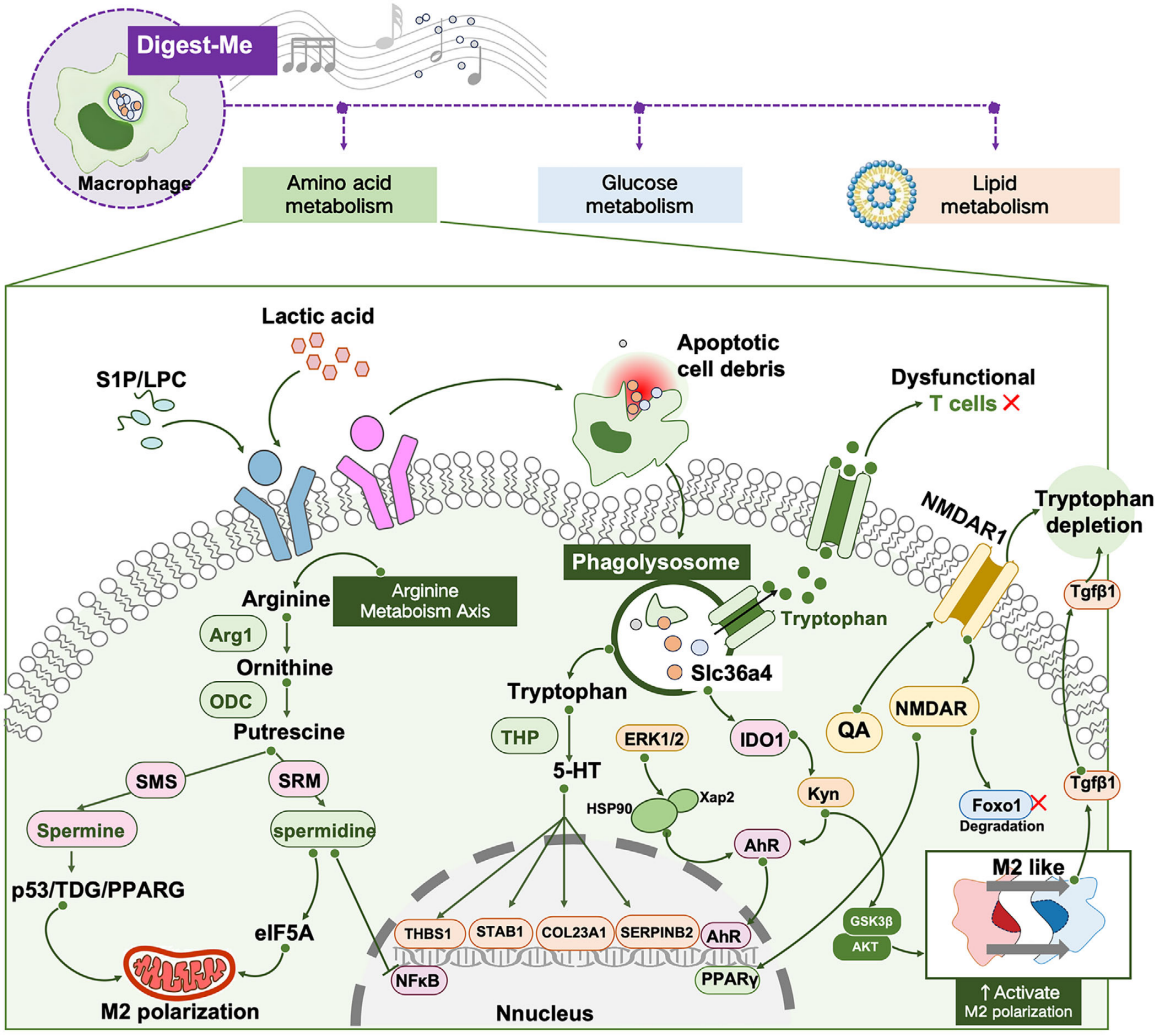
Effect of amino acid metabolism on macrophage polarization. In the tumor microenvironment, efferocytosis reprograms arginine and tryptophan metabolism in macrophages, driving their polarization toward an immunosuppressive M2 phenotype. In arginine metabolism, efferocytosis‐associated signals, including S1P and LPC, induce aberrant activation of the ARG1/2 pathway, redirecting arginine catabolism toward ornithine production and polyamine synthesis, including putrescine, spermidine, and spermine. Polyamine accumulation promotes M2 polarization through suppression of p65/NF‐κB signaling and regulation of eIF5A hypusination. In tryptophan metabolism, efferocytosis upregulates the SLC36A4 transporter and IDO1 expression, promoting the conversion of tryptophan into kynurenine (Kyn) and activation of the AhR signaling, thereby facilitating M2 polarization. Kyn can further drive M2 polarization via the quinolinic acid (QA)/NMDAR axis. In addition, tryptophan depletion impairs T cell function and promotes immune evasion.

##### 2.3.1.1 Arginine Metabolism

After macrophages engulf apoptotic cells, arginine from these cells is repurposed within macrophages. Macrophages process arginine via the NO synthesis pathway and the arginase pathway, with the regulatory influence of arginine on M1/M2 polarization being contingent on the differing activities of iNOS and Arg1 in these pathways [144]. In the “find me” phase, signals like S1P and LPC from apoptotic tumor cells enhance Arg1 expression in macrophages, leading them to shift from an iNOS‐dependent to an Arg1‐dependent metabolic pathway [145, 146, 147]. Through the aberrant activation of the Arg1 pathway, arginine is processed into ornithine and then further broken down into putrescine by the action of ornithine decarboxylase. Putrescine is further converted into spermidine and spermine by spermidine synthase and spermine synthase, respectively. In the tumor microenvironment, enhanced Arg1 activity increases substrate availability for polyamine biosynthesis, leading to higher levels of putrescine, spermidine, and spermine. Excessive polyamines not only enhance tumor cell proliferation and metabolic but also promote macrophages to polarize toward an M2‐like phenotype, fostering an immunosuppressive tumor microenvironment. Concurrently, the depletion of arginine mediated by Arg1 decreases the availability of substrates for iNOS, leading to a reduced production of NO and the impairment of effector T cell and natural killer (NK) cell function, ultimately strengthening immune suppression [148]. In breast cancer, Arg1/2 increases spermine production and facilitates M2‐like macrophage polarization through the p53/TDG/PPARG signaling pathway, resulting in the inhibition of CD8^+^ T cell activation via the release of IL‐10, PD‐L1, and TGFB1 [149]. In colon cancer, ODC depletion in macrophages directly reduced putrescine synthesis while also activating histone modification markers (H3K4me1 and H3K9ac) and upregulating M1 markers (NOS2, TNF‐α, IL‐1α, and IL‐1β) [150].

Consistently, Li et al. [151] reported that macrophages experience metabolic reprogramming after engulfing chemotherapy‐induced apoptotic tumor cells, resulting in elevated intracellular putrescine levels. In vitro studies show that spermidine inhibits p65/NF‐κB signaling in macrophages, facilitating their transition from the M1 to the M2 subtype [152]. Puleston and colleagues [153] showed that the hypusination of eIF5A, triggered by spermidine, alters mitochondrial metabolic processes and affects how macrophages polarize in tumor environment. Conversely, inhibiting the Arg1 activity can effectively reprogram macrophages into an antitumor phenotype. Wang et al. [154] demonstrated that the natural compound physcion‐3‐O‐β‐d‐glucopyranoside in combination with indocyanine green inhibits Arg1 activity, increases NO production, and promotes M1‐like polarization. However, arginine metabolism in the tumor microenvironment is regulated not only by Arg1 and iNOS but also by the concentration‐dependent availability of local substrates. In melanoma, arginine supplementation activates the iNOS/NO signaling pathway and increases M1‐type markers IL‐12 and TNF‐α [155]. In the glioma microenvironment with sufficient arginine, ADI‐PEG20 triggers metabolic changes (downregulation of Arg1 and upregulation of NOS2) through arginine depletion, while maintaining NO production via the citrulline regeneration pathway, thereby enhancing M1 macrophage polarization [156].

In summary, arginine is a key substrate for macrophage metabolism, while also serving as a common substrate for inducible iNOS and Arg1/2. In the tumor microenvironment, the competition for arginine by these enzymes significantly influences macrophage polarization. Tumor‐associated macrophages usually show high Arg1 levels, which channel arginine into polyamine production, thus sustaining M2 polarization and inhibiting effector immune cell activity. Conversely, arginine supplementation increases substrate availability, which partially reduces competition mediated by Arg1 and restores the activity of iNOS pathway to boost NO production. This process enhances the expression of M1‐type‐associated immune factors, alters the immunosuppressive microenvironment, and promotes antitumor immunity. Therefore, targeting arginine metabolism to restore the iNOS/ARG1 balance could mitigate the protumor effects of tumor‐associated macrophages and suppress tumor growth.

##### 2.3.1.2 Tryptophan Metabolism

During efferocytosis, apoptotic cells that are engulfed by macrophages are hydrolyzed in the phagolysosome, releasing a rich source of amino acids, including tryptophan. Tryptophan is transported from phagolysosomes to the cytosol via posttranscriptionally upregulated SLC36A4 and is subsequently converted into kynurenine (Kyn) by the upregulated IDO1, which activates the aryl hydrocarbon receptor (AhR) [157]. Meanwhile, ERK1/2 signaling activated throughout the “find me” and “eat me” stages promotes AhR nuclear translocation by upregulating HSP90/XAP2 [157]. These processes also promote the polarization of macrophages toward the M2‐type, forming an immunosuppressive microenvironment and facilitating tumor immune escape. In vivo and in vitro research has shown that efferocytosis in the tumor microenvironment enhances macrophage intracellular tryptophan and Kyn levels through lysosomal hydrolysis and improved tryptophan transport [158]. Tryptophan‐induced 5‐HT enhances the activation of M2‐related genes, such as SERPINB2, THBS1, STAB1, and COL23A1, while suppressing the expression of M1‐related genes, including INHBA, CCR2, MMP12, and PAI‐1 [159, 160]. Increasing evidence suggests that the IDO1–Kyn pathway plays a significant part in macrophage polarization. In patients with advanced oral squamous cell carcinoma, the abundance of IDO1^+^ macrophages in the intratumoral area is higher than that in the peritumoral area [161]. Similarly, advanced soft tissue sarcoma is marked by a higher plasma Kyn‐to‐tryptophan ratio and increased IDO1 expression in M2 macrophages [162]. Wang et al. [163] discovered that YKL‐40 upregulates IDO1 expression, activating the Trp/Kyn metabolic pathway, which supports M2‐like polarization and promotes tumor progression in gallbladder cancer. Conversely, inhibition of IDO1 effectively reprograms macrophage polarization and enhances the antitumor activity. Wen et al. [164] utilized H‐MOF nanomedicine to specifically target and inactivate the active site of IDO1 through sonodynamic biobromination, which polarized macrophages toward the M1 phenotype and induced a robust antitumor response. Moreover, knockout of the IDO1 gene significantly increases the expression of M1‐associated markers such as TNF‐α, IL‐6, and IL‐12 [165]. Likewise, lowering Kyn levels inhibits M2‐like TAM polarization and boosts antitumor immune responses [166]. In glioblastoma, macrophages metabolize Kyn to quinolinic acid (QA) through KYNU, which then activates the NMDAR/Foxo1/PPARγ pathway, resulting in Foxo1 degradation and increased PPARγ levels, ultimately promoting M2 polarization [167]. Some studies have found that AhR activation attenuates M1 macrophage polarization and promotes M2 macrophage polarization [45]. In esophageal squamous cells, Kyn activates AhR signaling and induces an immunosuppressive phenotype in TAMs through the AKT/GSK3β/IL‐8 pathway [168]. When oxygen is scarce, the competition between AhR and HIF‐1α for ARNT binding restricts HIF‐1α‐induced glycolytic activity and dampens M1‐associated inflammatory responses [169]. It should be mentioned that how tryptophan metabolism affects macrophage polarization varies greatly between different tumor types. In breast cancer, there is a positive correlation between tryptophan metabolic activity and M1 macrophage polarization [170]. This apparent discrepancy might be attributed to the unique metabolic microenvironments found in tumors across different anatomical sites. The immunological outcomes resulting from tryptophan metabolism are potentially determined by the integrated influence of metabolic enzymes, metabolites, and receptor expression on macrophages.

Besides its role in regulating macrophage polarization, tryptophan is also crucial for T cell function, and its metabolic disorders can result in T cell dysfunction and apoptosis. Tumor cells also competitively consume tryptophan, thereby establishing a metabolic environment that promotes immune suppression. Jiang et al. [171] found that the tryptophan transporter SLC7A5 and IDO are significantly upregulated in colon cancer cells, allowing these tumor cells to compete with T cells for tryptophan, thus hindering CD8^+^ T cell differentiation and function. Conversely, the deletion of IDO1 leads to decreased PD‐1/PD‐L1 expression and enhanced infiltration of CD8^+^ T cells [172]. In conclusion, the accumulation of tryptophan metabolites in the TME is a key factor influencing M2‐like TAM polarization and may act as a significant biomarker for assessing tumor immunotherapy effectiveness.

#### 2.3.2 Glucose Metabolism—Lactate

How are glucose uptake and glycolysis enhanced in macrophages that clear apoptotic cells? Research indicates that the three phases of efferocytosis systematically regulate the glucose metabolic reprogramming of macrophages to enhance sustained phagocytic activity and remodel the tissue microenvironment [173]. During the “find me” phase, apoptotic cells release soluble factors like ATP that activate the kinase SGK1 in macrophages, which subsequently phosphorylates GLUT1 to enhance its localization in the plasma membrane, facilitating glucose uptake. In the “eat me” phase, macrophages recognize PS on apoptotic cells through specific receptors, triggering SLC2A1 transcription and increasing glucose uptake and glycolytic activity, which promotes lactate production. During the “digest me” stage, macrophages increase SLC16A3‐mediated lactate release after engulfing apoptotic cells. Besides macrophages undergoing efferocytosis, tumor cells exposed to hypoxia and increased glycolysis also excrete substantial quantities of lactate into the tumor microenvironment [174]. These observations raise an intriguing question: whether lactate regulates macrophage polarization, reshaping the immunosuppressive microenvironment?

Growing evidence suggests that lactate functions as a signaling molecule that promotes M2‐like macrophage polarization and alters the tumor microenvironment to an immunosuppressive state (Figure 5). In lung adenocarcinoma, macrophages expressing high levels of LDHA activate the PI3K/AKT and MAPK pathways through SPP1–CD44, facilitating the polarization of tumor‐associated macrophages toward the M2 phenotype [175]. In pancreatic cancer, LDHA deficiency enhances CD8^+^ T cell antitumor immunity and promotes polarization toward antitumor M1 macrophages, ultimately improving immunotherapeutic efficacy [176]. Similarly, in mouse‐derived BMDMs, LDHA knockout or glycolytic inhibition reduces lactate production, the expression of the M2 macrophage marker Arg1, and histone lactylation on its promoter [177]. In breast cancer, the expression of M2‐specific markers, such as MRC‐1 and Arg‐1, is stimulated by lactate via the activation of ERK/STAT3 signaling [178]. In gliomas, GPR65 on macrophages senses lactate stimulation and promotes HMGB1 secretion through the cAMP–CREB signaling pathway, resulting in polarization toward the protumor M2 phenotype [179]. Additionally, in breast cancer, lactate binds to GPR132 in macrophages, triggering intracellular calcium release, activating the HIF‐1α signaling pathway, and increasing Arg‐1 expression, which in turn enhances the expression of M2 markers like CD206, Arg‐1, GM‐CSF, and CCL17 [180]. Consistently, Chen et al. [181] showed that inhibiting lactate efflux in the tumor microenvironment reduces M2‐type tumor‐associated macrophage polarization and effectively inhibits breast cancer growth and metastasis.

**FIGURE 5:**
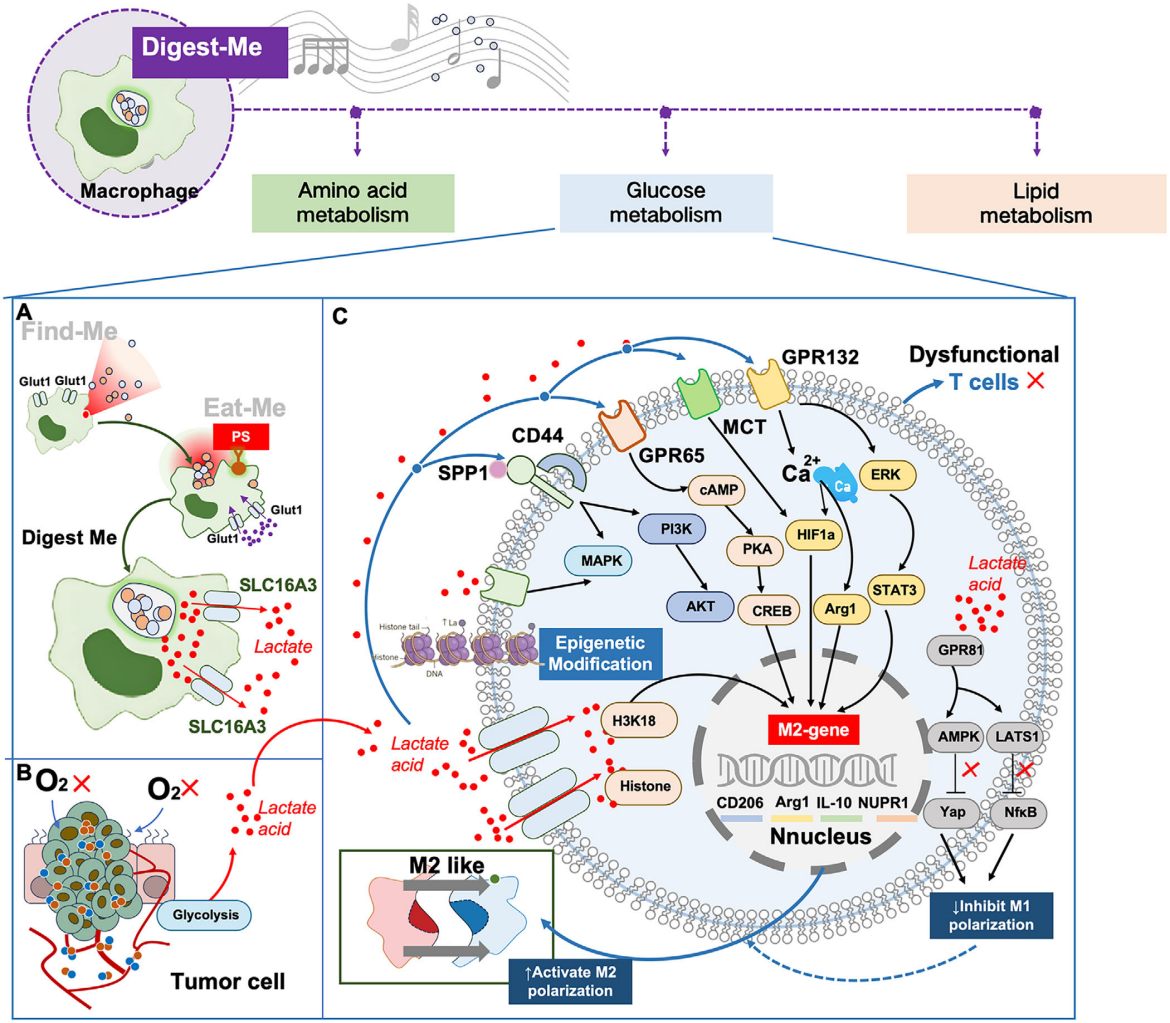
Regulatory role of lactate derived from glycolysis on macrophage polarization. (A) The three stages of efferocytosis sequentially reprogram the glucose uptake and glycolysis processes in macrophages. In the “find me” stage, factors such as ATP released by apoptotic cells upregulate the kinase SGK1, which facilitates the plasma membrane localization of GLUT1 through phosphorylation. Upon entering the “eat me” stage, recognition of PS receptors induces the transcription of SLC2A1, leading to a significant increase in glucose uptake and glycolysis. During the “digest me” stage, the upregulation of SLC16A3 mediates lactate efflux. (B) Tumor cells also contribute to the release of lactate in the tumor microenvironment. (C) The lactate produced by efferocytosis synergizes with lactate derived from tumor cells to promote macrophage polarization toward the M2‐like phenotype through multiple mechanisms: (1) The receptor‐mediated signaling pathway—lactate activates the cAMP/CREB, Ca2+/HIF1α, and ERK/STAT3 pathways via receptors such as GPR65 and GPR132, leading to the upregulation of M2 markers like Arg1 and CD206. (2) Metabolic enzyme‐dependent regulation—high expression of LDHA promotes M2 polarization through histone lactylation. (3) Epigenetic modification—lactate enhances the transcription of M2‐related genes via H3K18 lactylation, while simultaneously inhibiting M1‐type inflammatory functions through the GPR81/AMPK/LATS1 pathway.

It is worth mentioning that lactate alters the characteristics of macrophages via epigenetic modification. In hepatocellular carcinoma, the factor SRSF10 maintains MYB transcript stability, which leads to the elevation of glycolytic enzymes such as GLUT1, HK1, and LDHA, significantly increasing lactate levels within the tumor microenvironment [182]. Increased lactate levels induce histone H3K18 lactylation, which promotes the expression of M2 macrophage‐related genes such as CD206, ARG1, IL10, and ADM. These M2 macrophages additionally enhance immunosuppression and resistance to anti‐PD‐1 treatment by inhibiting the activation of CD8^+^ T cells [182]. Moreover, lactate induces the expression of NUPR1 through histone lactylation, inhibits the JNK signaling pathway in macrophages, promotes M2 polarization, and leads to the failure of HCC immunotherapy [183]. In Lewis lung carcinoma and melanoma, macrophages absorb lactate via MCT1‐4 transporters under acidic conditions, which activates HIF1α and leads to the upregulation of M2‐type markers like Arg1, Vegf, Fizz1, and Mgl1/2 [184]. Liao et al. [185] successfully repolarized M2 macrophages to an M1 phenotype by eliminating lactate from the tumor microenvironment with hydrogel‐released lactate oxidase.

Importantly, lactate not only promotes M2 polarization but also suppresses the inflammatory activities of M1 macrophages. In HCC, lactate inhibits immune‐like activation of M1 macrophages through downregulation of Zhx2 expression [186]. In bone marrow‐derived macrophages, lactate activates AMPK and LATS1 through the GPR81 receptor, inhibiting YAP and NF‐κB activities and reducing M1‐associated inflammatory mediators [187]. Collectively, lactate acts as a key metabolic and signaling mediator that enhances the immunosuppressive tumor microenvironment by facilitating M2 macrophage polarization and inhibiting M1‐related inflammatory responses. These findings indicate that targeting lactate metabolism could be an effective approach for modulating macrophage polarization and addressing resistance to cancer immunotherapy.

#### 2.3.3 Lipid Metabolism

In the digestion phase, macrophages absorb a significant quantity of fatty acids from apoptotic cells, which are then transported into mitochondria by carnitine palmitoyl transferase 1 (CPT1), leading to a marked increase in fatty acid β‐oxidation (FAO) and subsequent metabolic reprogramming. The study by Zhang et al. [188] further confirmed that efferocytosis promotes macrophage uptake of fatty acids and oxygen. Moreover, in the tumor microenvironment, increased lactate elevates METTL14 expression through H3K18la, raises VSIG4 levels via m6A–YTHDC1, activates the JAK2–STAT3–PPAR pathway, thereby enhancing FAO [189]. Growing evidence suggests that FAO activation in the tumor microenvironment is linked to M2 macrophage polarization and tumor progression (Figure 6). In HCC, FAO‐related gene expression is increased, leading to the activation of the PPAR–STAT3 signaling pathway, which promotes the differentiation of TAMs into M2 macrophages and raises the M2/M1 macrophage ratio in tumors [190]. Conversely, lowering fatty acid levels in the tumor microenvironment or inhibiting fatty acid oxidation can effectively reverse M2 macrophage polarization and improve tumor immunotherapy effectiveness. Albakri et al. [191] demonstrated that the removal of fatty acids from tumor cell supernatant or the use of FAO inhibitors reversed the phenotypic changes in macrophages induced by fatty acids. Similarly, in colitis‐associated colorectal cancer, the Fabp2 inhibitor effectively blocks STAT6 phosphorylation, reduces fatty acid oxidation and lipid uptake, and shift macrophages toward the M2 phenotype [192]. Besides the classical FAO metabolic pathway, lipids also promote the polarization of M2 macrophages through other signaling pathways. In the gastric cancer, lipids facilitate M2 macrophage polarization via PI3K‐γ activation, while PI3K‐γ‐targeting drugs inhibit the tumor‐supporting role of M2 macrophages [193].

**FIGURE 6:**
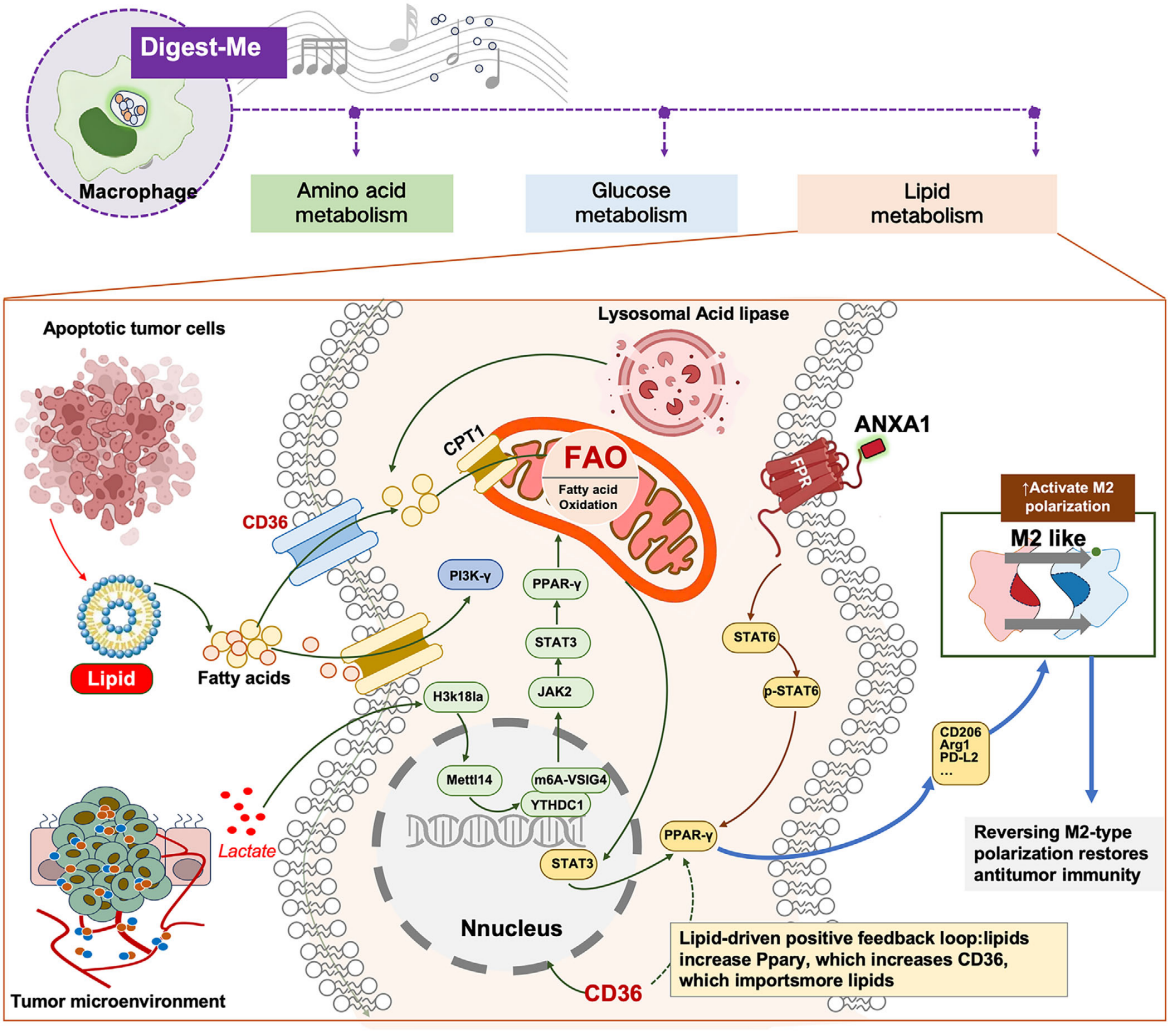
Efferocytosis‐driven lipid metabolic reprogramming orchestrates M2 polarization in the TME. Upon the efferocytosis of apoptotic tumor cells, macrophages internalize exogenous fatty acids (FAs) via CD36. These FAs are hydrolyzed by lysosomal acid lipase (LAL) and transported into mitochondria through carnitine palmitoyltransferase 1 (CPT1) to fuel fatty acid β‐oxidation (FAO), establishing a metabolic signature characterized by lipid accumulation and enhanced FAO. Concurrently, lactate in the tumor microenvironment upregulates METTL14 through histone lactylation (H3K18la), which enhances VSIG4 transcription via the m6A–YTHDC1 axis, subsequently activating the JAK2/STAT3–PPARγ cascade to further potentiate FAO and M2 polarization. ANXA1 activates the FPR2–STAT6–PPARγ axis, which transcriptionally upregulates CD36, forming a lipid‐driven positive feedback loop: lipids upregulate PPARγ, PPARγ increases CD36, and CD36 imports additional lipids. The convergence of these pathways, including PPARγ, STAT3, STAT6, and PI3K‐γ, promotes M2‐like polarization, upregulates M2 markers (CD206, Arg1, PD‐L2), and reinforces immunosuppression and tumor progression.

Importantly, the three stages of efferocytosis in the regulation of macrophage polarization are not isolated from each other. During efferocytosis, signals from the “find me” and “eat me” stages upregulate the transcription and expression of PPAR, CD36, AnxA1, AMPK, and others. PPAR serves as a key transcription factor for FAO and is essential for M2‐type activation [194]. AnxA1 induces STAT6 phosphorylation and PPARγ signaling activation by activating FPR2, thereby upregulating the transcriptional level of CD36 [195]. In the mouse tumor transplantation model, the upregulation of PPAR‐γ and its target gene CD36, a fatty acid transporter, reprogrammed the mitochondrial metabolism of macrophages, increased fatty acid oxidation, and effectively promoted M2 phenotype polarization [196]. Wu et al. [197] demonstrated that PPAR‐γ agonists promote a transition from lipid‐induced M1 polarized macrophages to the M2 phenotype, whereas PPAR‐γ antagonists produce the opposite effect. In turn, lipid metabolism products further activate PPAR signaling, forming a positive feedback regulatory mechanism. In hepatocellular carcinoma, high‐fat metabolism activates the PPAR‐γ signaling in macrophages, leading to M2 macrophage polarization and enhancing the invasion and migration of tumor cells [198]. Huang et al. [199] demonstrated that CD36 facilitated the uptake of exogenous lipids by macrophages in vitro, increased FAO following hydrolysis by lysosomal acid lipase, and stimulated the expression of M2 marker genes (CD206, CD301, PD‐L2, and RELMα). Knockout of fatty acid translocase CD36 impairs the ability of macrophages to promote M2 phenotype polarization [200]. Xu et al. [201] found that AMPK activation enhances fatty acid oxidation, reduces M1 markers by 49%, and increases M2 markers by 3.3 times, further supporting the view that reprogramming lipid metabolism promotes M2 polarization.

In summary, fatty acids from apoptotic cells via efferocytosis, lactate from glycolysis, and signaling factors during the efferocytosis phases together promote macrophage polarization to an immunosuppressive M2 phenotype through FAO‐related metabolic reprogramming. This process leads to the development of a tumor microenvironment that suppresses immune responses and causes resistance to immunotherapy. Thus, targeting FAO during efferocytosis may be a valuable approach to reshape the tumor microenvironment and enhance antitumor immune responses.

### 2.4 M2 Macrophages Remodel the Microenvironment and Promote Angiogenesis via Tumor Drug Resistance

In the tumor microenvironment, the engulfment of apoptotic tumor cells by macrophages triggers the transformation of macrophages into an immunosuppressive M2‐like phenotype. M2‐like macrophages, as a crucial functional phenotype of tumor‐associated macrophages, play a pivotal role in creating an immunosuppressive environment by dampening antitumor immunity, recruiting immunosuppressive cells, promoting angiogenesis, and using other mechanisms to drive tumor progression. They are also closely associated with drug resistance. Therefore, investigating the specific mechanisms through which M2 macrophages mediate the tumor immunosuppressive environment and drug resistance is vitally important for reshaping the immune microenvironment, addressing tumor drug resistance, and improving patient survival outcomes.

#### 2.4.1 Remodeling the Immunosuppressive Tumor Microenvironment

M2‐like macrophages are capable of shaping the immunosuppressive tumor microenvironment through multiple mechanisms, facilitating tumor immune evasion and contributing to the development of therapeutic resistance. Studies have indicated a significant increase in the expression of M2‐type macrophage‐related genes in drug‐resistant tumors, suggesting a potential association between the enrichment of M2‐type macrophages, immunosuppression, and resistance to tumor therapy [202]. M2 macrophages directly inhibit T cell activation and proliferation by expressing immune checkpoint ligands and releasing cytokines. Within the tumor microenvironment, M2‐type macrophages exhibit higher levels of PD‐L1 expression compared with M0 and M1‐type macrophages [203]. Their surface PD‐L1 can bind to PD‐1 on T cells, leading to the inhibition of T cell activation and effector functions, weakening antitumor immunity. Upregulating PD‐L1 expression in M2‐type macrophages has been found to effectively enhance tumor immunosuppression and reduce the therapeutic efficacy of anti‐PD‐1/PD‐L1 treatment [204]. Additionally, M2‐like TAMs can enhance immunosuppression by modulating Tregs through the secretion of chemokines such as CCL17, CCL20, and CCL22, which specifically recruit regulatory T cells expressing CCR4 or CCR6 to accumulate in tumor tissues [205]. Furthermore, TGF‐β and IL‐10 secreted by M2 macrophages contribute to the formation of an immunosuppressive environment associated with Tregs, promoting the generation and enhancing the immunosuppressive function of Foxp3 Tregs [206]. TGF‐β and IL‐10 can also directly suppress the functions of cytotoxic T lymphocytes, as well as Th1 and Th2 CD4+ T cells [207, 208]. Additionally, M2‐type macrophages can impact innate antitumor immunity by inducing an exhausted NK cell phenotype characterized by CD27low/CD11bhigh, leading to reduced NK cell activation and cytotoxic function [209].

Furthermore, M2‐like macrophages are capable of inhibiting antitumor immune responses through metabolic alteration. These macrophages express elevated levels of Arg1, which results in the depletion of L‐arginine in the local environment, leading to reduced TCR/CD3 ζ chain expression and hindering T cell proliferation and effector functions [205]. Additionally, IDO‐mediated tryptophan–Kyn metabolism can lead to local tryptophan depletion and accumulation of Kyn metabolites, further suppressing T cell and NK cell functions [205]. These conclusions suggest that M2‐like TAMs can concurrently restrict T cell‐ and NK cell‐mediated antitumor immunity through interconnected mechanisms, including immune checkpoint inhibition, Treg recruitment and functional regulation, immunosuppressive cytokines, and metabolic regulation, thereby reshaping the immunosuppressive tumor microenvironment. Together, these effects collectively contribute to tumor immune evasion and may decrease tumor sensitivity to treatment, thus contributing to the development of therapeutic resistance.

#### 2.4.2 Secreted Growth Factors Mediate Vascular Remodeling and Therapeutic Resistance

M2‐like macrophages are involved in conferring resistance to tumor therapy through mechanisms such as promoting tumor vascular remodeling and enhancing DNA damage repair by secreting various growth factors. Among these, VEGF plays a crucial role in mediating tumor angiogenesis and therapeutic resistance. In lung adenocarcinoma, M2 macrophages induce resistance to docetaxel in tumor cells by increasing the expression levels of VEGF‐C and VEGFR3 [210]. Similarly, Stockmann et al. [211] demonstrated that specific knockout of macrophage‐derived VEGF‐A improved tumor vascular structure and enhanced the sensitivity of Lewis lung carcinoma to cyclophosphamide and cisplatin. Moreover, platelet‐derived growth factor BB (PDGF‐BB) derived from M2‐like macrophages can also promote tumor angiogenesis. In colorectal cancer, PDGF‐BB specifically secreted by M2‐like macrophages has been shown to significantly promote angiogenesis in vivo and in vitro [212]. Growth factors derived from M2‐like macrophages can influence therapeutic response through nonangiogenic mechanisms as well. Fu et al. [213] found that M2‐like macrophages activate EGFR by releasing HB‐EGF, which enhances the nonhomologous end‐joining repair pathway and induces radioresistance in HPV‐negative head and neck squamous cell carcinoma. Additionally, insulin‐like growth factor (IGF) is also closely associated with resistance to various treatments. Elevated plasma IGF‐1 levels or high expression of IGF‐1R in tumors was significantly correlated with resistance to PD‐1 immunotherapy in NSCLC patients [214]. In esophageal squamous cell carcinoma, the IGF1R inhibitor linsitinib enhances the sensitivity of cisplatin chemotherapy [215]. Furthermore, the IGF‐1R inhibitor figitumumab combined with paclitaxel and carboplatin has shown potential in improving treatment outcomes for advanced solid tumors [216]. The above studies illustrate that M2‐like macrophages can modulate the tumor vascular microenvironment and DNA damage repair by secreting growth factors, thereby creating a microenvironment conducive to tumor cell survival, reducing their sensitivity to antitumor therapy, and promoting therapeutic resistance.

## 3 Targeting the Efferocytosis Signaling is a Therapeutic Strategy

In tumor therapy, with a deeper understanding of the tumor microenvironment and immune regulatory mechanisms, antitumor strategies targeting efferocytosis‐related signaling receptors have emerged as a promising research direction. In recent years, an increasing number of studies have highlighted the unique advantages and therapeutic potential of targeting efferocytosis signaling receptors in cancer therapy, providing new targets and strategies for drug development. Numerous studies indicate that current antitumor approaches targeting efferocytosis primarily focus on the “find me” and “eat me” signals. In this section, we aim to systematically review the progress in the development of antitumor drugs targeting efferocytosis‐related signaling pathways. We further analyze the current status of clinical research and discuss the opportunities and challenges of this emerging field, aiming to provide useful references for researchers and clinicians and to advance the development of this innovative area (Table 1).

**TABLE 1:** Antitumor efficacy of efferocytosis‐targeted strategies in clinical studies.

Phase	Drug/target	Combined regimens	Cancer	Sample size	primary endpoint	Efficacy	Status	NCT number/source
Find me/chemotaxis	Suramin/S1PRs	Paclitaxel and carboplatin	Advanced NSCLC	58	ORR	ORR was 36% in the chemotherapy‐naïve group and 0% in the chemotherapy‐resistant group.	Completed	[217]
	Suramin/S1PRs	Docetaxel	Advanced NSCLC	80	PFS	mPFS was 2.8 months in the docetaxel group versus 1.6 months in the docetaxel plus suramin group.	Completed	NCT01671332
	Suramin/S1PRs	Mitomycin C	Hormone‐resistant/refractory prostate cancer	32	Response rate	The overall benefit rate was 68.5%, and the objective response rate was 21.8%.	Completed	[218]
	Sonepcizumab/S1PRs	Monotherapy	Metastatic renal cell carcinoma	40	PFS	The ITT population had a median PFS of only 2.2 months.	Terminated	[219]
	ABC294640/SphK‐2	Monotherapy	Relapsed/refractory multiple myeloma	13	Maximum tolerated dose	The maximum tolerated dose was not reached in the study.	Terminated	NCT02757326
	ABC294640/SphK‐2	/	Advanced solid tumors	22	Maximum tolerated dose	500 mg bid	Completed	NCT01488513
Eat me/engulfment	Bavituximab/PS	Radiation and temozolomide	Newly‐diagnosed glioblastoma	36	Overall survival at 12 months	Unclear	Completed	NCT03139916
	Bavituximab/PS	Docetaxel	Advanced NSCLC	597	OS	Bavituximab plus docetaxel did not improve OS (10.5 vs. 10.9 months).	Completed	NCT01999673
	Bavituximab/PS	Pembrolizumab	HCC	35	ORR	32.1%	Active, not recruiting	NCT03519997
	Bavituximab/PS	Radiotherapy + temozolomide	Newly diagnosed glioblastoma	36	OS	The 12‐month OS rate was 72.7%, and the median OS was 15.4 months.	Completed	NCT03139916
	Sitravatinib/TAM	Nivolumab	Nonsquamous NSCLC	156	ORR	CPI‐naive patients had the highest ORR (25.0%), followed by those with prior clinical benefit (16.9%), and those without prior clinical benefit had the lowest (11.4%).	Terminated	NCT02954991
	Sitravatinib/TAM	Tislelizumab	Advanced biliary tract cancer	43	Disease control rate	65.1% (95% CI 50.3–78.0)	Active, not recruiting	NCT04727996
	Sitravatinib/TAM	Nivolumab/ipilimumab	Advanced ccRCC	22	Safety	86.4%	Completed	NCT04518046
	Cabozantinib/TAM	Atezolizumab	RCC	522	OS/PFS	No significant differences in median PFS (10.6 vs. 10.8 months; HR = 1.03, *p* = 0.78) or OS (HR = 0.94, *p* = 0.69) between the combination and monotherapy groups	Terminated	NCT04338269
	Cabozantinib/TAM	Nivolumab/ipilimumab	Advanced RCC	855	PFS	The 12‐month PFS rate was 0.57 in the experimental group versus 0.49 in the control group.	Active, not recruiting	NCT03937219
	Cabozantinib/TAM	Monotherapy	Advanced and progressing HCC	707	OS	The median OS was 10.2 months in the cabozantinib group versus 8.0 months in the placebo group.	Completed	NCT01908426
	Batiraxcept/GAS6	Nivolumab/cabozantinib	Advanced ccRCC	72	Safety/ORR	The ORR was 43% in the two‐drug combination group, 55% in the three‐drug combination group, and 0% in the monotherapy group.	Terminated	NCT04300140
	Bemcentinib/Axl	Cytarabine	AML or MDS	72	ORR	The overall ORR was 25%, and the mOS was 8.4 months.	Completed	NCT02488408
	Bemcentinib/Axl	Monotherapy	HR‐MDS or AML	45	Overall hematologic response	The overall response rate was 24%.	Completed	NCT03824080
	Tamnorzatinib/Axl	Venetoclax	AML or MDS	42	ORR	No complete response or partial response was observed.	Terminated	NCT03176277
Do not eat me	Hu5F9‐G4/CD47	Rituximab	Non‐Hodgkin's lymphoma	22	ORR	ORR was 50%, and CR was 36%.	Terminated	NCT02953509
	Magrolimab/CD47	Azacitidine	AML	87	Safety/tolerability/CR	The CR rate was 32.6%, and the ORR was 74.7%.	Terminated	NCT03248479
	Hu5F9‐G4/CD47	Monotherapy	Advanced solid tumors and lymphomas	62	Safety/tolerability/MTD	The drug demonstrated favorable safety and tolerability, and the maximum tolerated dose was not reached.	Completed	NCT02216409
	Magrolimab/CD47	Azacitidine	TP53‐mutated AML	257	OS	The median OS was inferior to the control group in both the nonintensive and intensive groups (4.4 vs. 6.6 months; 7.3 vs. 11.1 months).	Terminated	NCT04778397
	Evorpacept/CD47	Pembrolizumab/trastuzumab	Advanced or metastatic solid tumor	110	MTD	Neither group reached the maximum tolerated dose.	Completed	NCT03013218
	Evorpacept/CD47	Lenalidomide/rituximab	Relapsed refractory B‐NHL	20	Safety/tolerability	The best ORR was 90%, and the best CR was 80%.	Active, not recruiting	NCT05025800
	Hu5F9‐G4/CD47	Venetoclax/azacitidine	AML	378	OS	The addition of magrolimab to venetoclax plus azacitidine did not improve efficacy, with a median OS of 10.7 versus 14.1 months.	Terminated	NCT05079230
Digestion/degradation	INCB001158/Arg1	Retifanlimab	Advanced or metastatic solid tumors	18	Safety/tolerability/MTD	No response or disease stabilization was observed.	Completed	NCT03910530

During the “find me” phase, current clinical research mainly focuses on the S1P and SphK‐2 signaling pathways. Representative drugs such as suramin [220, 221, 222], sonepcizumab [219], and ABC294640 [223, 224, 225] are currently at various stages of clinical evaluation, aiming to block the recruitment and activation of protumor macrophages at their source. However, existing clinical studies show that the therapeutic effects of drugs targeting the “find me” phase are unsatisfactory. For example, there are significant differences in the efficacy of suramin among various NSCLC patients [217]. In patients with advanced NSCLC receiving initial chemotherapy, the objective response rate (ORR) is 36%, indicating limited efficacy. However, it was completely ineffective in the chemotherapy‐resistant population, where the ORR was 0%. Furthermore, the combination of suramin and docetaxel shortened the progression‐free survival (PFS) to 1.6 months compared with 2.8 months. This may be related to its broad target profile and narrow therapeutic window. In newly diagnosed patients, tumors exhibit sensitivity to chemotherapy and a strong dependence on growth factors. Suramin, as a broad‐spectrum antagonist, can exert antitumor effects when used in combination with chemotherapy. However, in populations with drug resistance, the tumor microenvironment undergoes remodeling the immunosuppression, while mechanisms of multidrug resistance lead to increased drug efflux. Additionally, the off‐target toxicity of suramin, along with its interference with docetaxel in the recruitment of beneficial immune cells, may diminish efficacy and even produce antagonistic effects. The median PFS for sonepcizumab monotherapy in metastatic renal cell carcinoma was merely 2.2 months, leading to the eventual termination of this study [219]. This outcome suggests that solely blocking the S1P signal is insufficient to reverse the immunosuppressive tumor microenvironment.

During the “eat me” phase, the combination of PSR inhibitors with anti‐PD‐1/anti‐PD‐L1 drugs or chemotherapeutic agents has gained significant interest. In unresectable hepatocellular carcinoma, the combination of bavituximab (a PS‐targeting agent) and pembrolizumab (an anti‐PD‐1 antibody) has shown synergistic effects without increasing toxic effects [226]. Similarly, in patients with advanced nonsquamous NSCLC who have undergone prior treatment with checkpoint inhibitors (CPIs) or chemotherapy, the combination of xitrovatinib (a tyrosine kinase inhibitor) and nivolumab (an anti‐PD‐1 antibody) has exhibited antitumor activity and improved survival outcomes [227]. Collectively, these findings suggest that combinations of PSR inhibitors and PD‐1/PD‐L1 inhibitors may boost antitumor immune responses and enhance treatment effectiveness. However, such benefits have not been consistently observed when PSR inhibitors are combined with chemotherapeutic agents. In the SUNRISE trial, docetaxel combined with bavituximab showed inferior efficacy than docetaxel monotherapy, with no improvement in clinical outcomes was observed for patients with advanced NSCLC [228]. The key reason for this discrepancy may stem from their distinct mechanisms of synergistic action. A synergistic impact against tumors is achieved by combining PSR inhibitors with anti‐PD‐1/anti‐PD‐L1 drugs, fundamentally due to their ability to simultaneously engage both innate and adaptive immune. PSR inhibitors improve the effectiveness of PD‐1/PD‐L1 inhibitors by preventing PS recognition by AXL/TYRO3/MERTK receptors on tumor‐associated macrophages, reducing M2 polarization, facilitating M1‐like phenotype transformation, and increasing T cell infiltration. By contrast, radiotherapy and chemotherapy induce extensive tumor cell apoptosis, generating large amounts of apoptotic cells. These apoptotic cells engage PS‐dependent efferocytosis pathways and promote M2‐like macrophage polarization, contributing to an immunosuppressive tumour microenvironment that may counteract the effects of PSR inhibition. Consequently, future approaches should focus on combining PSR inhibitors with immune CPIs. If chemotherapy is necessary, optimized approaches like sequential treatment or low‐dose protocols may reduce antagonism.

In clinical studies focusing on TAM receptors and their ligand GAS6 for antitumor therapy, research has mainly concentrated on AXL inhibitors. Preclinical research indicates that targeting AXL and MERTK may offer several mechanistic benefits that enhance their therapeutic potential. These mechanisms include modulation of postphagocytic signaling to prevent secondary necrosis, inhibition of immunosuppressive signaling via the AXL/GAS6/PS pathway, promotion of macrophage polarization toward an antitumor M1‐like phenotype, reduction of PD‐L1 expression, and attenuation of resistance to EGFR or FLT3 targeted therapies. For example, batiraxcept (AXL inhibitor) combined with cabozantinib is significantly superior to monotherapy in ccRCC patients [229]. Nonetheless, it is important to recognize that the true effectiveness of most clinical studies is significantly lower than anticipated. Recent studies have shown that the combination of AVB‐S6‐500 (an AXL inhibitor) and durvalumab failed to demonstrate antitumor activity in patients with platinum‐resistant ovarian cancer [230]. In addition, in patients with high‐risk myelodysplastic syndromes (MDS) or AML, the ORR for bemcentinib monotherapy is merely 24%. The ORR for bemcentinib in combination with cytarabine is 25%, showing no synergistic effect. In contrast, the combination of tamnorzatinib with venetoclax showed completely ineffective, with an ORR of 0% [231, 232]. These findings suggest that the clinical efficacy of AXL‐targeted therapies may vary substantially across tumor types. This variability may indicate differences in how tumors rely on AXL signaling and the broader biological variations among individuals with cancer. For example, clear cell renal cell carcinoma appears to exhibit substantial reliance on AXL‐associated signaling pathways, potentially contributing to the favorable activity observed with batiraxcept plus cabozantinib. In contrast, the limited effectiveness observed in platinum‐resistant ovarian cancer may suggest that AXL signaling is not a key oncogenic factor in this background and may be insufficient to counteract the immunosuppressive tumor microenvironment. In blood cancers like AML and MDS, both intrinsic and adaptive resistance mechanisms may further reduce the effectiveness of AXL inhibition. Previous studies have indicated that inhibition of AXL signaling may lead to compensatory activation of alternative TAM family receptors, such as MERTK and TYRO3, or a shift toward reliance on antiapoptotic proteins like MCL‐1 and BCL‐xL, thus decreasing the effectiveness of therapy [233]. Consequently, the clinical benefit of AXL‐targeted approaches is likely to depend on specific tumor biological characteristics and the identification of appropriate combination strategies. Currently, most drugs targeting TAM receptors remain in the basic and clinical research phases [234]. Future studies should focus on identifying predictive biomarkers, determining sensitive tumor types for TAM‐targeted therapy, and developing effective combination therapy strategies to enhance clinical efficacy.

In clinical trials of medications targeting CD47, effectiveness varies significantly across different tumors and patient groups. By expressing high levels of CD47, tumor cells send “don't eat me” signals to macrophages, allowing them to escape immune surveillance and clearance, which makes CD47 an attractive therapeutic target in cancer immunotherapy. Nonetheless, numerous studies have shown that the efficacy of CD47 antibody monotherapy is very limited. This limitation may arise from the fact that CD47 is expressed not only on tumor cells but also widely expressed on healthy cells. This results in fewer binding sites for antibodies on tumor cells after they attach to healthy cells, potentially leading to serious side effects like anemia and thrombocytopenia, thus restricting the therapeutic dosage and effectiveness [235, 236, 237]. It is noteworthy that the advent of novel CD47 antibodies has somewhat mitigated these limitations. By optimizing antibody design, these agents have overcome several of the aforementioned challenges and shown promising clinical efficacy in trials. For example, in lymphoma, the ORR of Hu5F9‐G4 combined with rituximab for the treatment of non‐Hodgkin lymphoma is 50%, with a complete response (CR) rate of 36% [238]. In patients with relapsed or refractory B‐cell non‐Hodgkin lymphoma (B‐NHL), the optimal overall response rate (ORR) of Evorpacept in combination with lenalidomide and rituximab was 90%, with a CR rate of 80% [239]. These results indicate that combination therapies may display significant therapeutic advantages in specific lymphoma populations. However, in AML, multiple combination therapy studies have not achieved positive results. In summary, although the novel CD47 antibody overcomes the dose‐limiting toxicity of single‐agent therapy to a certain extent by optimizing the design, the efficacy of CD47‐targeted drugs still shows obvious program and population heterogeneity. Combined immunochemotherapy showed a high remission rate in lymphoma, but has not yet translated into survival benefit in AML, and most related studies have been terminated.

In addition to the above reported clinical studies, most targeted therapy clinical trials for refractory, recurrent, or drug‐resistant tumors have not published their results due to factors like poor safety, unsatisfactory efficacy, or incomplete research (Table 2). These findings collectively reveal the clinical translation challenges of efferocytosis‐related targets in cancer therapy, including limited efficacy, safety concerns, and low research completion, while also reflecting significant heterogeneity in treatment responses across different targets and tumor types.

**TABLE 2:** Clinical trials with unknown outcomes for recurrent or drug‐resistant cancer treatment.

Target	Drug	Combined regimens	Cancer	Sample size	Status	NCT number/source
PS	Bavituximab	/Pembrolizumab	Refractory advanced solid tumor malignancies	28	Completed	NCT00129337
		/Pembrolizumab	Recurrent/metastatic squamous cell head and neck cancer	7	Active, not recruiting	NCT04150900
	BXQ‐350	/	Relapsed solid tumors	9	Terminated	NCT03967093
TIM	TSR‐022	Dostarlimab	Metastatic or recurrent cervical cancer	66 (estimated)	Recruiting	NCT06238635
	LY3321367	LY3300054	Advanced relapsed/refractory solid tumors	209	Completed	NCT03099109
	Sym023	Sym021/Sym022/irinotecan hydrochloride	Recurrent advanced selected solid tumor malignancies	78	Completed	NCT04641871
	TQB2618	Pempulimab/cisplatin/gemcitabine hydrochloride	Recurrent/metastatic nasopharyngeal carcinoma	47	Unknown status	NCT05563480
		Penpulimab injection	Relapsed and refractory lymphoma	92 (estimated)	Unknown status	NCT05400876
		Penpulimab, paclitaxel, cisplatin or carboplatin	Recurrent/metastatic head and neck squamous cell carcinoma	60 (estimated)	Recruiting	NCT05783921
	INCAGN02390	Retifanlimab/INCAGN02385	PD‐L1‐positive recurrent/metastatic squamous cell carcinoma of the head and neck	176	Active, not recruiting	NCT05287113
Axl	BA3011	Durvalumab	Platinum‐resistant high grade serous ovarian cancer	60 (estimated)	Recruiting	NCT04918186
	Bemcentinib	Surgery	Recurrent glioblastoma	10	Terminated	NCT03965494
		Rucaparib/abemaciclib/pembrolizumab	Recurrence malignant mesothelioma	186	Unknown status	NCT03654833
	CCT301‐38	/	AXL positive recurrent or refractory sarcoma	9	Terminated	NCT05128786
		/CCT301‐59	Recurrent or refractory Stage IV renal cell carcinoma	66	Terminated	NCT03393936
	Tilvestamab	/	Relapsed, platinum‐resistant, high‐grade serous ovarian cancer	16	Terminated	NCT04893551
	Amuvatinib	Platinum–etoposide chemotherapy	Nonresponsive or recurrent small cell lung cancer	23	Completed	NCT01357395
	Cabozantinib	/	VEGFR TKI or PD‐1 inhibitors unresponsive renal cell carcinoma	201 (estimated)	Unknown status	NCT04714697
		/	Relapsed or refractory myeloma	11	Completed	NCT01866293
		/	Recurrent liver cancer posttransplant	20 (estimated)	Recruiting	NCT04204850
		/	Advanced or metastatic cervical carcinoma after platinum treatment failure	57	Completed	NCT04205799
	Batiraxcept	Paclitaxel	Recurrent high grade uterine cancer	Unclear	Withdrawn	NCT05826015
		Doxorubicin or paclitaxel	Platinum‐resistant recurrent ovarian cancer	53	Completed	NCT03639246
		AVB‐S6‐500 + durvalumab	Platinum‐resistant or recurrent ovarian, fallopian tube, or primary peritoneal cancer	12	Completed	NCT04019288
		Paclitaxel	Platinum‐resistant recurrent ovarian cancer	366	Terminated	NCT04729608
		Cabozantinib	Advanced or metastatic renal cell carcinoma	49	Completed	NCT03463681
MERTK	MRX2843	/	Relapsed/refractory acute myeloid leukemia	104 (estimated)	Unknown status	NCT04946890
		/	Relapsed/refractory advanced and/or metastatic solid tumors	42	Active, not recruiting	NCT03510104
		/	Relapsed/refractory AML, ALL, or MPAL	50 (estimated)	Recruiting	NCT04872478
	ONO‐7475	Venetoclax	Relapsed or refractory acute myeloid leukemia or relapsed or refractory myelodysplastic syndromes	42	Terminated	NCT03176277
AXL, MERTK, CSF1R	Q702	Azacitidine and venetoclax	Relapsed or refractory acute myeloid leukemia	32 (estimated)	Recruiting	NCT06445907
AXL, MER, TYRO3, VEGF, c‐MET	Sitravatinib	Tislelizumab	Recurrent/metastatic cervical cancer	Unclear	Withdrawn	NCT05614453
		Pembrolizumab	Recurrent endometrial cancer and other solid tumors	Unclear	Withdrawn	NCT05419817
		Tislelizumab/Nab‐paclitaxel	Locally recurrent or metastatic triple negative breast cancer	98	Active, not recruiting	NCT04734262
CD47	Magrolimab	Lemzoparlimab	Relapsed and refractory non‐Hodgkin's lymphoma	98	Completed	NCT03934814

The data were from ClinicalTrials.gov (https://clinicaltrials.gov/).

## 4 Challenges and Future Prospects

In the tumor microenvironment, macrophages that have phagocytosed apoptotic cancer cells polarize into the M2 phenotype. Polarized M2 macrophages inhibit effector T cell activity by secreting immunosuppressive factors, reducing antitumor immunity and promoting angiogenesis and tumor metastasis. By inhibiting efferocytosis, macrophages within the tumor microenvironment can be shifted from M2 to M1 phenotype, which disrupts immune suppression and boosts antitumor responses. While this approach shows clear therapeutic potential, current research predominantly concentrates on basic studies or preliminary clinical trials that target a single stage of the efferocytosis process. This situation largely reflects the multifaceted challenges faced in the development of drugs targeting efferocytosis. Preclinical research results indicate that targeting efferocytosis receptors to reverse immunosuppression may lead to the release of self‐antigens, triggering potential autoimmune inflammatory diseases like age‐dependent systemic lupus erythematosus [240]. Nonetheless, there is no clinical research to definitively assess whether systemic anti‐PS therapy may aggravate the autoimmune response in individuals predisposed to lupus or patients with antiphospholipid antibody syndrome. Moreover, the high structural homology among the TAM receptor family implies that the double knockout or triple knockout of Axl and MerTK may result in systemic autoimmune diseases [241], platelet dysfunction [242], lymphocyte proliferation [243], infertility [244], and other related complications. Most importantly, the majority of existing inhibitors can result in the secondary necrosis of apoptotic cells and the release of their contents following the nonspecific blockade of normal efferocytosis functions through systemic administration. This, in turn, leads to systemic inflammation and tissue damage.

It is worth noting that, in addition to the above challenges, it is also related to the highly coupled nature of efferocytosis. The three phases of efferocytosis do not occur in isolation but instead constitute a tightly orchestrated and dynamically ongoing process of clearing apoptotic cells. During the “find me” phase, apoptotic cells release various soluble signaling molecules, including nucleotides (ATP, UTP), lipids (S1P, LPC), and chemokines (CX3CL1). These signals not only provide spatial guidance for phagocytes but also upregulate the expression of “eat me” receptors and intracellular digestion‐related molecules in advance. Poon et al. [245] demonstrated that S1P prompts macrophages to produce erythropoietin, upregulating MerTK, Gas6, and CD36 to facilitate subsequent phagocytosis and digestion. During the “eat me” phase, phagocytes quickly activate the ELMO1/DOCK complex and GTPases like Rac1 upon recognizing signals such as PS, leading to actin cytoskeleton rearrangement and phagosome formation [246]. Under the regulation of the Rab GTPase family, the phagosome rapidly matures and fuses with the lysosome, entering a highly acidic (pH 4.5–5.0) digestive stage [247, 248]. In professional phagocytes such as macrophages, the process of phagocytosis and internalization occurs very rapidly. Internalization takes only a few minutes, while intracellular digestion can be completed in 1–2 min. During the “digestion” phase, phagocytes consistently eliminate numerous apoptotic cells, and the byproducts from the digested apoptotic cells can serve as energy to facilitate the subsequent cycle of efferocytosis. This process necessitates a significant overlap of time and energy between the digestion phase and the new round of efferocytosis. Yurdagul et al. [249] demonstrated that phagocytes utilize arginine from digested apoptotic cells to enhance polyamine production and activate Rac1, thus facilitating cytoskeletal rearrangements and promoting the uptake of apoptotic cells in subsequent phases. Moreover, fatty acids ingested during digestion regulate the metabolic reprogramming of macrophages and promote the secretion of anti‐inflammatory factors, thus maintaining the microenvironment homeostasis necessary for efferocytosis. This efficient coupling allows for the rapid and safe removal of apoptotic tumor cells before they undergo secondary necrosis and release harmful substance. The three stages of efferocytosis are interconnected, and disrupting any of these connections will affect the other stages due to the feedback mechanism. This results in the inability of short‐term remission rates to translate into long‐term survival benefits, which may be the underlying reason for the shortcomings in most current antitumor drug development.

Single‐cell sequencing studies have expanded our understanding beyond the traditional M1/M2 binary polarization model, revealing a high degree of functional heterogeneity within the tumor microenvironment of TAMs. While classification schemes for TAM subsets vary across studies, several representative subpopulations have been identified, including SPP1 TAMs, C1Q TAMs, TREM2 TAMs, FOLR2 TAMs, and MARCO TAMs. Integration of single‐cell analysis and machine learning features has identified efferocytosis‐related genes, providing a new reference for analyzing the functional heterogeneity of TAMs. In colorectal cancer, C1QC TAMs highly express C1Q, TREM2, and CXCL10, and exhibit strong efferocytic activity and features that promote T cell activation [250]. In contrast, SPP1 TAMs exhibit high expression of SPP1 and VEGFA, which is correlated with tumor growth and metastasis [250]. In triple‐negative breast cancer, proresolving macrophages with high TREM2 and C1q expression can promote efferocytosis while impairing CD8 T cell metabolic function through C1q [251]. The efferocytosis effect in different TAM subsets may produce different or even opposite immunological effects, indicating that the functional status of TAMs is a dynamic process regulated by tumor microenvironment signals.

In recent years, some researchers have integrated advanced targeting technologies like nanomaterials, cell engineering methods, and hydrogels to deliver efferocytosis‐related receptor inhibitors locally. These methods are anticipated to lower the dosage or shorten the duration of certain standard antitumor treatments, promoting local immune disinhibition of tumors while reducing the autoimmune effects of systemic treatment. Liu et al. [252] designed a single‐atom nanozyme that promotes antitumor immunity through the induction of cuproptosis in tumor cells and the suppression of efferocytosis in macrophages. Huang et al. [253] employed gene editing to permanently knock out MerTK in the B16 melanoma model, which increased X‐ray irradiation‐induced immunogenic cell death by inhibiting efferocytosis. On this basis, the combination of anti‐PtdSer antibody and radiotherapy stimulated a sustained in situ vaccination‐like immune response and achieved a lasting tumor control effect. Furthermore, Ren and colleagues designed a Mn‐Ce6 nanocomposite encapsulated within a hybrid membrane of red blood cells and liposomes, which was modified with PtdSer aptamer [254]. It induces tumor cell apoptosis through light irradiation and competitively binds to PtdSer, which hinders macrophage clearance of apoptotic cells and enhances their M1 polarization.

It is important to highlight that precise spatiotemporal control over efferocytosis regulation is as crucial as effective targeting. Excessive activation of efferocytosis can hinder the initial inflammatory response necessary for antitumor immunity, while prolonged or excessive inhibition may increase the risk of autoimmune diseases or persistent inflammation [255, 256, 257]. This study envisions leveraging single‐cell spatial omics technologies to accurately identify and target specific macrophage subsets involved in efferocytosis within distinct tumor microenvironments. On this basis, the potential pleiotropic consequences that may arise from efferocytosis regulation are proactively incorporated into the nanomedicine design framework. Simultaneously, the synergistic optimization of efficacy and safety can be realized through the implementation of safety mechanisms, including exposure time window restrictions, self‐destruction modules, and timed‐release inhibitors. Meanwhile, we can achieve targeted payload release specifically within the tumor microenvironment by incorporating stimuli‐responsive or gated nanomaterials with spatiotemporal regulation capabilities, minimizing the effects on healthy tissues. We also address its time‐dependent contradiction through a two‐phase progressive design that first boosts efferocytosis to effectively eliminate apoptotic cells and prevent secondary necrosis, and subsequently reverses macrophage polarization to stimulate antitumor immunity. This design not only fully considers the dual biological effects of efferocytosis in tumor progression but also provides new research ideas for achieving precise, safe, and regulatable efferocytosis‐targeted therapy. Furthermore, given that nanomedicines are more complex to produce than small molecules or traditional biologics, their unique physicochemical properties (particle size, morphology, surface properties, etc.) are susceptible to minor variations in processing parameters [257]. We should either streamline the design as much as possible or use biomaterials that can be produced more easily at scale, while clearly defining and managing essential quality attributes like particle size distribution, polydispersity, zeta potential, drug loading, encapsulation efficiency, and release characteristics. In order to maximize the therapeutic effectiveness, reliable biomarkers and advanced imaging tools are necessary to achieve dynamic monitoring and precise assessment of efferocytosis in the body. However, there remains an absence of standardized clinical biomarkers that have been thoroughly validated and widely recognized to specifically monitor efferocytosis activity. In the future, new monitoring tools that provide real‐time monitoring and regulation of efferocytosis within the tumor microenvironment should be created by combining single‐cell omics, spatial omics, nanotechnology, and molecular imaging techniques.

## Author Contributions

Q.C. drafted the manuscript. C.W. reviewed the manuscript and provided essential suggestions. C.H. participated in literature screening and analysis of relevant studies. H.Z. supervised the work. All authors have read and approved the final manuscript.

## Funding

This work was supported by the National Natural Science Foundation of China (82374314), the National Science and Technology Major Project (2024ZD0526203), and the Sichuan Provincial Natural Science Foundation (2026NSFSC2098).

## Ethics Statement

The authors have nothing to report.

## Conflicts of Interest

The authors declare no conflicts of interest.

## Data Availability

Data availability is not applicable to this review as no new data were created or analyzed in this study.

## References

[1] R. L. Siegel , K. D. Miller , H. E. Fuchs , and A. Jemal , “Cancer Statistics, 2021,” CA: A Cancer Journal for Clinicians 71, no. 1 (2021): 7–33.. 10.3322/caac.21654 33433946

[2] J. Zhang , Z. Wang , X. Zhang , et al., “Large‐Scale Single‐Cell and Bulk Sequencing Analyses Reveal the Prognostic Value and Immune Aspects of CD147 in Pan‐Cancer,” Frontiers in Immunology 13 (2022): 810471, 10.3389/fimmu.2022.810471. PMC9019465 35464411

[3] H. Sung , J. Ferlay , R. L. Siegel , et al., “Global Cancer Statistics 2020: GLOBOCAN Estimates of Incidence and Mortality Worldwide for 36 Cancers in 185 Countries,” CA: A Cancer Journal for Clinicians 71, no. 3 (2021): 209–249. 10.3322/caac.21660 33538338

[4] D. Li , Y. Wang , C. Li , et al., “Cancer‐Specific Calcium Nanoregulator Suppressing the Generation and Circulation of Circulating Tumor Cell Clusters for Enhanced Anti‐Metastasis Combinational Chemotherapy,” Acta Pharmaceutica Sinica B 11, no. 10 (2021): 3262–3271, 10.1016/j.apsb.2021.04.009. PMC8546850 34729314

[5] W. Yu , X. Zhang , W. Zhang , et al., “19‐Hydroxybufalin Inhibits Non‐Small Cell Lung Cancer Cell Proliferation and Promotes Cell Apoptosis via the Wnt/β‐Catenin Pathway,” Experimental Hematology & Oncology 10, no. 1 (2021): 48, 10.1186/s40164-021-00243-0. PMC8543904 34696818

[6] A. Tajbakhsh , S. M. Gheibi Hayat , A. Movahedpour , et al., “The Complex Roles of Efferocytosis in Cancer Development, Metastasis, and Treatment,” Biomedicine & Pharmacotherapy 140 (2021): 111776, 10.1016/j.biopha.2021.111776. 34062411

[7] P. Mehrotra and K. S. Ravichandran , “Drugging the Efferocytosis Process: Concepts and Opportunities,” Nature Reviews Drug Discovery 21, no. 8 (2022): 601–620, 10.1038/s41573-022-00470-y. PMC9157040 35650427

[8] E. Boada‐Romero , J. Martinez , B. L. Heckmann , and D. R. Green , “The Clearance of Dead Cells by Efferocytosis,” Nature Reviews Molecular Cell Biology 21, no. 7 (2020): 398–414, 10.1038/s41580-020-0232-1. PMC7392086 32251387

[9] Y. Astuti , M. Raymant , V. Quaranta , et al., “Efferocytosis Reprograms the Tumor Microenvironment to Promote Pancreatic Cancer Liver Metastasis,” Nature Cancer 5, no. 5 (2024): 774–790, 10.1038/s43018-024-00731-2. PMC11136665 38355776

[10] S. Roy , A. K. Bag , S. Dutta , et al., “Macrophage‐Derived Neuropilin‐2 Exhibits Novel Tumor‐Promoting Functions,” Cancer Research 78, no. 19 (2018): 5600–5617, 10.1158/0008-5472.CAN-18-0562. PMC6168405 30111533

[11] X. Wang , Q. Pan , Y. Li , et al., “Chemotherapy Triggers Immune Evasion by Fostering LEPR+ Kupffer Cell Differentiation in Liver Metastases,” Cancer Cell 44, no. 3 (2026): 658–675.e12, 10.1016/j.ccell.2026.01.010, S1535‐6108(26)00049‐8. 41687606

[12] Z. Li , B. Xu , P. Sharma , et al., “Exclusion of Notch From the Contact Site During Efferocytosis Restricts Anticancer Immunity,” Nature Immunology 27, no. 4 (2026): 750–761, 10.1038/s41590-026-02452-3. PMC13043306 41776100

[13] R. S. Cook , K. M. Jacobsen , A. M. Wofford , et al., “MerTK Inhibition in Tumor Leukocytes Decreases Tumor Growth and Metastasis,” Journal of Clinical Investigation 123, no. 8 (2013): 3231–3242, 10.1172/JCI67655. PMC3726162 23867499

[14] A. Tajbakhsh , N. Farahani , S. M. Gheibihayat , et al., “Autoantigen‐Specific Immune Tolerance in Pathological and Physiological Cell Death: Nanotechnology Comes Into View,” International Immunopharmacology 90 (2021): 107177, 10.1016/j.intimp.2020.107177. 33249046

[15] L. Cheng , B. Weng , C. Jia , et al., “The Expression and Significance of Efferocytosis and Immune Checkpoint Related Molecules in Pancancer Samples and the Correlation of Their Expression With Anticancer Drug Sensitivity,” Frontiers in Pharmacology 13 (2022): 977025, 10.3389/fphar.2022.977025. PMC9437300 36059952

[16] M. Cheng , S. Chen , K. Li , et al., “CD276‐Dependent Efferocytosis by Tumor‐Associated Macrophages Promotes Immune Evasion in Bladder Cancer,” Nature Communications 15, no. 1 (2024): 2818, 10.1038/s41467-024-46735-5. PMC10985117 38561369

[17] M. Yang , J. Liu , C. Piao , J. Shao , and J. Du , “ICAM‐1 Suppresses Tumor Metastasis by Inhibiting Macrophage M2 Polarization Through Blockade of Efferocytosis,” Cell Death & Disease 6, no. 6 (2015): e1780, 10.1038/cddis.2015.144. PMC4669827 26068788

[18] B. Moon , S. Yang , H. Moon , J. Lee , and D. Park , “After Cell Death: The Molecular Machinery of Efferocytosis,” Experimental & Molecular Medicine 55, no. 8 (2023): 1644–1651, 10.1038/s12276-023-01070-5. PMC10474042 37612408

[19] A. K. Narahari , A. J. Kreutzberger , P. S. Gaete , et al., “ATP and Large Signaling Metabolites Flux Through Caspase‐Activated Pannexin 1 Channels,” Elife 10 (2021): e64787, 10.7554/eLife.64787. PMC7806264 33410749

[20] X. Wang and D. Chen , “Purinergic Regulation of Neutrophil Function,” Frontiers in Immunology 9 (2018): 399, 10.3389/fimmu.2018.00399. PMC5837999 29545806

[21] É. Fige , J. Szendrei , L. Sós , et al., “Heme Oxygenase‐1 Contributes to Both the Engulfment and the Anti‐Inflammatory Program of Macrophages During Efferocytosis,” Cells 10, no. 3 (2021): 652, 10.3390/cells10030652. PMC8001822 33804125

[22] M. R. Elliott , F. B. Chekeni , P. C. Trampont , et al., “Nucleotides Released by Apoptotic Cells Act as a Find‐Me Signal to Promote Phagocytic Clearance,” Nature 461, no. 7261 (2009): 282–286, 10.1038/nature08296. PMC2851546 19741708

[23] K. K R Skényi , E. Duró , A. Pallai , et al., “Involvement of Adenosine A2A Receptors in Engulfment‐Dependent Apoptotic Cell Suppression of Inflammation,” Journal of Immunology (Baltimore, Md: 1950) 186, no. 12 (2011): 7144–7155. 10.4049/jimmunol.1002284 PMC3395167 21593381

[24] S. Daniele , L. Natali , C. Giacomelli , et al., “Osteogenesis Is Improved by Low Tumor Necrosis Factor Alpha Concentration Through the Modulation of GS‐Coupled Receptor Signals,” Molecular and Cellular Biology 37, no. 8 (2017): e00442‐16, 10.1128/MCB.00442-16. PMC5376638 28137910

[25] H. K. Buist , U. Luchowska‐Stańska , B. Van Basten , et al., “Identification and Characterization of an Affimer Affinity Reagent for the Detection of the cAMP Sensor, EPAC1,” Cells 10, no. 9 (2021): 2307, 10.3390/cells10092307. PMC8465552 34571955

[26] L. Sommerfeld , I. Knuth , F. Finkernagel , et al., “Prostacyclin Released by Cancer‐Associated Fibroblasts Promotes Immunosuppressive and Pro‐Metastatic Macrophage Polarization in the Ovarian Cancer Microenvironment,” Cancers 14, no. 24 (2022): 6154, 10.3390/cancers14246154. PMC9776493 36551640

[27] J. Chen , V. Y. Shin , J. C.‐W. Ho , M.‐T. Siu , I. W.‐Y. Cheuk , and A. Kwong , “Functional Implications of Cathelicidin Antimicrobial Protein in Breast Cancer and Tumor‐Associated Macrophage Microenvironment,” Biomolecules 10, no. 5 (2020): 688, 10.3390/biom10050688. PMC7277779 32365569

[28] Y. Liu , G. Liu , J. Mei , et al., “GJB2 Drives LUAD Progression in Part via cAMP‐Mediated M2 Macrophage Polarization Through the PKA‐CREB Pathway,” Respiratory Research 27, no. 1 (2026): 98, 10.1186/s12931-026-03537-1. PMC12934008 41618295

[29] C. H. Serezani , M. N. Ballinger , D. M. Aronoff , and M. Peters‐Golden , “Cyclic AMP,” American Journal of Respiratory Cell and Molecular Biology 39, no. 2 (2008): 127–132, 10.1165/rcmb.2008-0091TR. PMC2720142 18323530

[30] S. Widodo , M. Dinevska , L. Cook , S. Stylli , and T. Mantamadiotis , “P02.17.B CREB Regulates Pro‐Tumor Macrophage Polarization in GBM and Can be Targeted to Reverse Immunosuppression,” Neuro‐Oncology 27, no. Sup3 (2010): 2.

[31] D. Ruffell , F. Mourkioti , A. Gambardella , et al., “A CREB‐C/EBPβ Cascade Induces M2 Macrophage‐Specific Gene Expression and Promotes Muscle Injury Repair,” Proceedings of the National Academy of Sciences of the United States of America 106, no. 41 (2009): 17475–17480, 10.1073/pnas.0908641106. PMC2762675 19805133

[32] T. Zhao , R. Li , M. Qian , et al., “Whole‐Transcriptome Analysis Highlights Adenylyl Cyclase Toxins‐Derived Modulation of NF‐κB and ERK1/2 Pathways in Macrophages,” Toxins 15, no. 2 (2023): 139, 10.3390/toxins15020139. PMC9967024 36828453

[33] L. P. Tavares , G. L. Negreiros‐Lima , K. M. Lima , et al., “Blame the Signaling: Role of cAMP for the Resolution of Inflammation,” Pharmacological Research 159 (2020): 105030, 10.1016/j.phrs.2020.105030. 32562817

[34] X. Wu , Z. Wang , J. Shi , et al., “Macrophage Polarization Toward M1 Phenotype Through NF‐κB Signaling in Patients With Behçet's Disease,” Arthritis Research & Therapy 24, no. 1 (2022): 249, 10.1186/s13075-022-02938-z. PMC9635113 36333776

[35] J. Xie , X. Wu , S. Zheng , K. Lin , and J. Su , “Aligned Electrospun Poly(l‐Lactide) Nanofibers Facilitate Wound Healing by Inhibiting Macrophage M1 Polarization via the JAK‐STAT and NF‐κB Pathways,” Journal of Nanobiotechnology 20, no. 1 (2022): 342, 10.1186/s12951-022-01549-9. PMC9327399 35883095

[36] M. Dai , L. Wu , Z. He , et al., “Epoxyeicosatrienoic Acids Regulate Macrophage Polarization and Prevent LPS‐Induced Cardiac Dysfunction,” Journal of Cellular Physiology 230, no. 9 (2015): 2108–2119, 10.1002/jcp.24939. PMC4467528 25626689

[37] J. Chen , N. Goyal , L. Dai , et al., “Developing New Ceramide Analogs and Identifying Novel Sphingolipid‐Controlled Genes Against a Virus‐Associated Lymphoma,” Blood 136, no. 19 (2020): 2175–2187, 10.1182/blood.2020005569. PMC7645984 32518949

[38] A. L. Lam and B. Heit , “Having an Old Friend for Dinner: The Interplay Between Apoptotic Cells and Efferocytes,” Cells 10, no. 5 (2021): 1265, 10.3390/cells10051265. PMC8161178 34065321

[39] A. Weigert , C. Olesch , and B. Brüne , “Sphingosine‐1‐Phosphate and Macrophage Biology—How the Sphinx Tames the Big Eater,” Frontiers in Immunology 10 (2019): 1706, 10.3389/fimmu.2019.01706. PMC6658986 31379883

[40] N. J. Pyne , F. Tonelli , K. G. Lim , J. S. Long , J. Edwards , and S. Pyne , “Sphingosine 1‐Phosphate Signalling in Cancer,” Biochemical Society Transactions 40, no. 1 (2012): 94–100, 10.1042/BST20110602. 22260672

[41] S. F. Spampinato , G. Costantino , S. Merlo , P. L. Canonico , and M. A. Sortino , “Microglia Contributes to BAF‐312 Effects on Blood–Brain Barrier Stability,” Biomolecules 12, no. 9 (2022): 1174, 10.3390/biom12091174. PMC9496388 36139013

[42] A. Weigert , N. Tzieply , A. Von Knethen , et al., “Tumor Cell Apoptosis Polarizes Macrophages—Role of Sphingosine‐1‐Phosphate,” Molecular Biology of the Cell 18, no. 10 (2007): 3810–3819, 10.1091/mbc.e06-12-1096. PMC1995721 17652460

[43] V. Barra , A.‐M. Kuhn , A. Von Knethen , A. Weigert , and B. Brüne , “Apoptotic Cell‐Derived Factors Induce Arginase II Expression in Murine Macrophages by Activating ERK5/CREB,” Cellular and Molecular Life Sciences 68, no. 10 (2011): 1815–1827, 10.1007/s00018-010-0537-x. PMC11115119 20949368

[44] J. E. Hughes , S. Srinivasan , K. R. Lynch , R. L. Proia , P. Ferdek , and C. C. Hedrick , “Sphingosine‐1‐Phosphate Induces an Antiinflammatory Phenotype in Macrophages,” Circulation Research 102, no. 8 (2008): 950–958, 10.1161/CIRCRESAHA.107.170779. PMC2875063 18323526

[45] X. Yang , H. Liu , T. Ye , et al., “AhR Activation Attenuates Calcium Oxalate Nephrocalcinosis by Diminishing M1 Macrophage Polarization and Promoting M2 Macrophage Polarization,” Theranostics 10, no. 26 (2020): 12011–12025, 10.7150/thno.51144. PMC7667681 33204326

[46] L. Arseni , R. Sharma , N. Mack , et al., “Sphingosine‐1‐Phosphate Recruits Macrophages and Microglia and Induces a Pro‐Tumorigenic Phenotype That Favors Glioma Progression,” Cancers 15, no. 2 (2023): 479, 10.3390/cancers15020479. PMC9856301 36672428

[47] M. Mrad , C. Imbert , V. Garcia , et al., “Downregulation of Sphingosine Kinase‐1 Induces Protective Tumor Immunity by Promoting M1 Macrophage Response in Melanoma,” Oncotarget 7, no. 44 (2016): 71873–71886, 10.18632/oncotarget.12380. PMC5342129 27708249

[48] R. Feng , Z. Cui , L. Yang , and Z. Liu , “Sphingosine 1‐Phosphate Derived From Tumor‐Educated Hepatic Stellate Cells Combining With S1PR4 Promotes Tumor Associated Macrophages Differentiation Through FAO Modulation,” Scientific Reports 15, no. 1 (2025): 20507, 10.1038/s41598-025-02588-6. PMC12218931 40592997

[49] S.‐J. Park , K.‐P. Lee , S. Kang , et al., “Sphingosine 1‐Phosphate Induced Anti‐Atherogenic and Atheroprotective M2 Macrophage Polarization Through IL‐4,” Cellular Signalling 26, no. 10 (2014): 2249–2258, 10.1016/j.cellsig.2014.07.009. 25035231

[50] C. S. Whyte , E. T. Bishop , D. Rückerl , et al., “Suppressor of Cytokine Signaling (SOCS)1 Is a Key Determinant of Differential Macrophage Activation and Function,” Journal of Leukocyte Biology 90, no. 5 (2011): 845–854, 10.1189/jlb.1110644. 21628332

[51] B. Weichand , R. Popp , S. Dziumbla , et al., “S1PR1 on Tumor‐Associated Macrophages Promotes Lymphangiogenesis and Metastasis via NLRP3/IL‐1β,” Journal of Experimental Medicine 214, no. 9 (2017): 2695–2713, 10.1084/jem.20160392. PMC5584110 28739604

[52] J. Liang , M. Nagahashi , E. Y. Kim , et al., “Sphingosine‐1‐Phosphate Links Persistent STAT3 Activation, Chronic Intestinal Inflammation, and Development of Colitis‐Associated Cancer,” Cancer Cell 23, no. 1 (2013): 107–120, 10.1016/j.ccr.2012.11.013. PMC3578577 23273921

[53] W. Tsai , S. Chang , Y. Lin , and H. Hsu , “CX3CL1(+) Microparticles‐Induced MFG‐E8 Enhances Apoptotic Cell Clearance by Alveolar Macrophages,” Cells 10, no. 10 (2021): 2583, 10.3390/cells10102583. PMC8533702 34685562

[54] M.‐J. Brissette , S. Lepage , A.‐S. Lamonde , et al., “MFG‐E8 Released by Apoptotic Endothelial Cells Triggers Anti‐Inflammatory Macrophage Reprogramming,” PLoS ONE 7, no. 4 (2012): e36368, 10.1371/journal.pone.0036368. PMC3340380 22558449

[55] L. Huang , Z. Wang , C. Liao , et al., “PVT1 Promotes Proliferation and Macrophage Immunosuppressive Polarization Through STAT1 and CX3CL1 Regulation in Glioblastoma Multiforme,” CNS Neuroscience & Therapeutics 30, no. 1 (2024): e14566, 10.1111/cns.14566. PMC10805395 38287522

[56] C. Sun , A. Hu , S. Wang , et al., “ADAM17‐Regulated CX3CL1 Expression Produced by Bone Marrow Endothelial Cells Promotes Spinal Metastasis From Hepatocellular Carcinoma,” International Journal of Oncology 57, no. 1 (2020): 249–263. 10.3892/ijo.2020.5045 PMC7252465 32319605

[57] X.‐W. Gao , H.‐L. Hu , M.‐H. Xie , C.‐X. Tang , J. Ou , and Z.‐H. Lu , “CX3CL1/CX3CR1 Axis Alleviates Inflammation and Apoptosis in Human Nucleus Pulpous Cells via M2 Macrophage Polarization,” Experimental and Therapeutic Medicine 26, no. 1 (2023): 359, 10.3892/etm.2023.12058. PMC10265713 37324510

[58] X. Feng , S. Zhu , J. Qiao , Z. Ji , B. Zhou , and W. Xu , “CX3CL1 Promotes M1 Macrophage Polarization and Osteoclast Differentiation Through NF‐κB Signaling Pathway in Ankylosing Spondylitis in Vitro,” Journal of Translational Medicine 21, no. 1 (2023): 573, 10.1186/s12967-023-04449-0. PMC10463543 37626378

[59] Y. He , J. Hu , A. Ma , et al., “The Crosstalk Between Efferocytosis and Macrophage Polarization in Diabetic Wounds: A Comprehensive Review,” Journal of Inflammation Research 19 (2026): 1–18, 10.2147/JIR.S566523. PMC13033258 41913789

[60] V. Palafox‐Sánchez , Z. Ying , L. F. F. Royes , and F. Gomez‐Pinilla , “The Interaction Between Brain and Liver Regulates Lipid Metabolism in the TBI Pathology,” Biochimica et Biophysica Acta (BBA)—Molecular Basis of Disease 1867, no. 4 (2021): 166078, 10.1016/j.bbadis.2021.166078. PMC7889047 33444711

[61] E. Abdelzaher and M. F. Mostafa , “Lysophosphatidylcholine Acyltransferase 1 (LPCAT1) Upregulation in Breast Carcinoma Contributes to Tumor Progression and Predicts Early Tumor Recurrence,” Tumor Biology 36, no. 7 (2015): 5473–5483, 10.1007/s13277-015-3214-8. 25683484

[62] Y. Morita , T. Sakaguchi , K. Ikegami , et al., “Lysophosphatidylcholine Acyltransferase 1 Altered Phospholipid Composition and Regulated Hepatoma Progression,” Journal of Hepatology 59, no. 2 (2013): 292–299, 10.1016/j.jhep.2013.02.030. 23567080

[63] C. Ye , X. Ru , and Y. Guo , “Scientometric Analysis of Lipid Metabolism in Macrophage Polarization: 2013‐2023,” Frontiers in Oncology 15 (2025): 1532862, 10.3389/fonc.2025.1532862. PMC12222308 40612347

[64] H. Li , J. Lyu , Y. Sun , et al., “S‐Acyl Transferase ZDHHC13 Modulates Tumor Microenvironment Interactions to Suppress Metastasis in Melanoma Models,” Journal of Clinical Investigation 135, no. 23 (2025): e188249, 10.1172/JCI188249. PMC12646662 41321310

[65] V. Ruipérez , J. Casas , M. A. Balboa , and J. Balsinde , “Group V Phospholipase A2‐Derived Lysophosphatidylcholine Mediates Cyclooxygenase‐2 Induction in Lipopolysaccharide‐Stimulated Macrophages,” Journal of Immunology (Baltimore, Md: 1950) 179, no. 1 (2007): 631–638.. 10.4049/jimmunol.179.1.631 17579085

[66] X. Qin , C. Qiu , and L. Zhao , “Lysophosphatidylcholine Perpetuates Macrophage Polarization Toward Classically Activated Phenotype in Inflammation,” Cellular Immunology 289, no. 1–2 (2014): 185–190, 10.1016/j.cellimm.2014.04.010. 24841857

[67] J. Wang , B. Wang , and Y. Zhang , “Agonism Activities of Lyso‐Phosphatidylcholines (LPC) Ligands Binding to Peroxisome Proliferator‐Activated Receptor Gamma (PPARγ),” Journal of Biomolecular Structure and Dynamics 38, no. 2 (2020): 398–409, 10.1080/07391102.2019.1577175. 31025599

[68] L. S. Assunção , K. G. Magalhães , A. B. Carneiro , et al., “Schistosomal‐Derived Lysophosphatidylcholine Triggers M2 Polarization of Macrophages Through PPARγ Dependent Mechanisms,” Biochimica et Biophysica Acta (BBA)—Molecular and Cell Biology of Lipids 1862, no. 2 (2017): 246–254, 10.1016/j.bbalip.2016.11.006. 27871882

[69] E. E. Torr , D. H. Gardner , L. Thomas , et al., “Apoptotic Cell‐Derived ICAM‐3 Promotes Both Macrophage Chemoattraction to and Tethering of Apoptotic Cells,” Cell Death & Differentiation 19, no. 4 (2012): 671–679, 10.1038/cdd.2011.167. PMC3307987 22117198

[70] L. Zhe , L. Fang , P. Olutomilayo Olayemi , et al., “M1 Macrophage‐Related Prognostic Model by Combining Bulk and Single‐Cell Transcriptomic Data in NSCLC,” Exploration of Medicine 5 (2024): 750–773.

[71] E. D'Angelo , R. Rampado , F. Sensi , et al., “Tumor Microenvironment‐Mimicking Macrophage Nanovesicles as a Targeted Therapy Platform for Colorectal Cancer,” International Journal of Pharmaceutics 670 (2025): 125169, 10.1016/j.ijpharm.2025.125169. 39756598

[72] W. Shen , J. Xie , S. Zhao , et al., “ICAM3 Mediates Inflammatory Signaling to Promote Cancer Cell Stemness,” Cancer Letters 422 (2018): 29–43, 10.1016/j.canlet.2018.02.034. PMC5928798 29477378

[73] A. Estecha , N. Aguilera‐Montilla , P. Sánchez‐Mateos , and A. Puig‐Kröger , “RUNX3 Regulates Intercellular Adhesion Molecule 3 (ICAM‐3) Expression During Macrophage Differentiation and Monocyte Extravasation,” PLoS ONE 7, no. 3 (2012): e33313, 10.1371/journal.pone.0033313. PMC3315569 22479382

[74] H. Liu and L. Zhang , “Decidual Macrophage Subsets and Polarization Puzzle During the Human Early Pregnancy,” Frontiers in Immunology 16 (2025): 1610891, 10.3389/fimmu.2025.1610891. PMC12301210 40726980

[75] A. Hashimoto , D. Sarker , V. Reebye , et al., “Upregulation of C/EBPα Inhibits Suppressive Activity of Myeloid Cells and Potentiates Antitumor Response in Mice and Patients With Cancer,” Clinical Cancer Research 27, no. 21 (2021): 5961–5978, 10.1158/1078-0432.CCR-21-0986. PMC8756351 34407972

[76] A. A. Baxter , M. D. Hulett , and I. K. Poon , “The Phospholipid Code: A Key Component of Dying Cell Recognition, Tumor Progression and Host–Microbe Interactions,” Cell Death & Differentiation 22, no. 12 (2015): 1893–1905, 10.1038/cdd.2015.122. PMC4816099 26450453

[77] J. Suzuki , D. P. Denning , E. Imanishi , H. R. Horvitz , and S. Nagata , “Xk‐Related Protein 8 and CED‐8 Promote Phosphatidylserine Exposure in Apoptotic Cells,” Science 341, no. 6144 (2013): 403–406, 10.1126/science.1236758. 23845944

[78] J. Lee , B. Park , B. Moon , et al., “A Scaffold for Signaling of Tim‐4‐Mediated Efferocytosis Is Formed by Fibronectin,” Cell Death & Differentiation 26, no. 9 (2019): 1646–1655, 10.1038/s41418-018-0238-9. PMC6748117 30451988

[79] S. Morioka , C. Maueröder , and K. S. Ravichandran , “Living on the Edge: Efferocytosis at the Interface of Homeostasis and Pathology,” Immunity 50, no. 5 (2019): 1149–1162, 10.1016/j.immuni.2019.04.018. PMC6721617 31117011

[80] T. Shibata , D. M. Habiel , A. L. Coelho , S. L. Kunkel , N. W. Lukacs , and C. M. Hogaboam , “Axl Receptor Blockade Ameliorates Pulmonary Pathology Resulting From Primary Viral Infection and Viral Exacerbation of Asthma,” The Journal of Immunology 192, no. 8 (2014): 3569–3581, 10.4049/jimmunol.1302766. PMC4509623 24659691

[81] S. Nepal , C. Tiruppathi , Y. Tsukasaki , et al., “STAT6 Induces Expression of Gas6 in Macrophages to Clear Apoptotic Neutrophils and Resolve Inflammation,” Proceedings of the National Academy of Sciences of the United States of America 116, no. 33 (2019): 16513–16518, 10.1073/pnas.1821601116. PMC6697797 31363052

[82] C. E. J. Waterborg , M. G. A. Broeren , E. N. Blaney Davidson , et al., “The Level of Synovial AXL Expression Determines the Outcome of Inflammatory Arthritis, Possibly Depending on the Upstream Role of TGF‐β1,” Rheumatology 58, no. 3 (2019): 536–546, 10.1093/rheumatology/key337. 30508140

[83] G. Zahuczky , E. Kristóf , G. N. Majai , and L. Fésüs , “Differentiation and Glucocorticoid Regulated Apopto‐Phagocytic Gene Expression Patterns in Human Macrophages. Role of Mertk in Enhanced Phagocytosis,” PLoS ONE 6, no. 6 (2011): e21349, 10.1371/journal.pone.0021349. PMC3123306 21731712

[84] T. Shibata , D. M. Habiel , A. L. Coelho , and C. M. Hogaboam , “Axl Receptor Blockade Protects From Invasive Pulmonary Aspergillosis in Mice,” The Journal of Immunology 193, no. 7 (2014): 3559–3565, 10.4049/jimmunol.1401258. 25156363

[85] J. Q. Braghini , M. C. Cabral Lorenzo , L. Kornblihtt , M. S. De Lorenzo , S. M. Ranuncolo , and G. Blanco , “Efferocytosis and M2 Macrophage Polarization Gene Expression Correlates With Relapsed and Refractory Classical Hodgkin Lymphoma Patients: A Multi‐Omic Analysis,” Hematological Oncology 43, no. 5 (2025): e70135, 10.1002/hon.70135. 40976706

[86] A. M. Grabiec , A. Goenka , M. E. Fife , T. Fujimori , and T. Hussell , “Axl and MerTK Receptor Tyrosine Kinases Maintain Human Macrophage Efferocytic Capacity in the Presence of Viral Triggers,” European Journal of Immunology 48, no. 5 (2018): 855–860, 10.1002/eji.201747283. PMC6001567 29400409

[87] L. Sanjurjo , G. Aran , É. Téllez , et al., “CD5L Promotes M2 Macrophage Polarization Through Autophagy‐Mediated Upregulation of ID3,” Frontiers in Immunology 9 (2018): 480, 10.3389/fimmu.2018.00480. PMC5858086 29593730

[88] T. Deng , Y. Zhang , Q. Chen , K. Yan , and D. Han , “Toll‐like Receptor‐Mediated Inhibition of Gas6 and ProS Expression Facilitates Inflammatory Cytokine Production in Mouse Macrophages,” Immunology 135, no. 1 (2012): 40–50, 10.1111/j.1365-2567.2011.03511.x. PMC3246651 22043818

[89] Y. Shen , X. Cui , Y. Rong , et al., “Exogenous Gas6 Attenuates Silica‐Induced Inflammation on Differentiated THP‐1 Macrophages,” Environmental Toxicology and Pharmacology 45 (2016): 222–226, 10.1016/j.etap.2016.05.029. 27327525

[90] S. H. Nam , D. Kim , D. Lee , et al., “Lysyl‐tRNA Synthetase–Expressing Colon Spheroids Induce M2 Macrophage Polarization to Promote Metastasis,” Journal of Clinical Investigation 128, no. 11 (2018): 5034–5055, 10.1172/JCI99806. PMC6205414 30188867

[91] S.‐Y. Kim , E.‐J. Lim , Y.‐S. Yoon , et al., “Liver X Receptor and STAT1 Cooperate Downstream of Gas6/Mer to Induce Anti‐Inflammatory Arginase 2 Expression in Macrophages,” Scientific Reports 6 (2016): 29673, 10.1038/srep29673. PMC4942780 27406916

[92] Z. Chang , C. Zhong , S. Xu , et al., “Multi‐Modal Integration of Histopathology and Transcriptomics Reveals STAB1+ Macrophage‐Associated Efferocytosis as a Suppressive Immune Mechanism in Colon Adenocarcinoma,” Journal of Translational Medicine 23, no. 1 (2025): 1386, 10.1186/s12967-025-07348-8. PMC12683853 41361444

[93] K. V. Myers , S. R. Amend , and K. J. Pienta , “Targeting Tyro3, Axl and MerTK (TAM Receptors): Implications for Macrophages in the Tumor Microenvironment,” Molecular Cancer 18, no. 1 (2019): 94, 10.1186/s12943-019-1022-2. PMC6515593 31088471

[94] Y. T. Akalu , C. V. Rothlin , and S. Ghosh , “TAM Receptor Tyrosine Kinases as Emerging Targets of Innate Immune Checkpoint Blockade for Cancer Therapy,” Immunological Reviews 276, no. 1 (2017): 165–177, 10.1111/imr.12522. PMC5381815 28258690

[95] X. Liang , M. Luo , B. Shao , et al., “Phosphatidylserine Released From Apoptotic Cells in Tumor Induces M2‐like Macrophage Polarization Through the PSR‐STAT3‐JMJD3 Axis,” Cancer Communications 42, no. 3 (2022): 205–222, 10.1002/cac2.12272. PMC8923121 35191227

[96] H. Wu , J. Zheng , S. Xu , et al., “Mer Regulates Microglial/Macrophage M1/M2 Polarization and Alleviates Neuroinflammation Following Traumatic Brain Injury,” Journal of Neuroinflammation 18, no. 1 (2021): 2, 10.1186/s12974-020-02041-7. PMC7787000 33402181

[97] B. Zhang , L. Fang , H.‐M. Wu , P.‐S. Ding , K. Xu , and R.‐Y. Liu , “Mer Receptor Tyrosine Kinase Negatively Regulates Lipoteichoic Acid‐Induced Inflammatory Response via PI3K/Akt and SOCS3,” Molecular Immunology 76 (2016): 98–107, 10.1016/j.molimm.2016.06.016. 27419619

[98] A. B. Lee‐Sherick , K. M. Eisenman , S. Sather , et al., “Aberrant Mer Receptor Tyrosine Kinase Expression Contributes to Leukemogenesis in Acute Myeloid Leukemia,” Oncogene 32, no. 46 (2013): 5359–5368, 10.1038/onc.2013.40. PMC3898106 23474756

[99] J. J. Boyle , “Heme and Haemoglobin Direct Macrophage Mhem Phenotype and Counter Foam Cell Formation in Areas of Intraplaque Haemorrhage,” Current Opinion in Lipidology 23, no. 5 (2012): 453–461, 10.1097/MOL.0b013e328356b145. 22777293

[100] A. Kadl , A. K. Meher , P. R. Sharma , et al., “Identification of a Novel Macrophage Phenotype That Develops in Response to Atherogenic Phospholipids via Nrf2,” Circulation Research 107, no. 6 (2010): 737–746, 10.1161/CIRCRESAHA.109.215715. PMC2941538 20651288

[101] E. Ubil , L. Caskey , A. Holtzhausen , D. Hunter , C. Story , and H. S. Earp , “Tumor‐Secreted Pros1 Inhibits Macrophage M1 Polarization to Reduce Antitumor Immune Response,” Journal of Clinical Investigation 128, no. 6 (2018): 2356–2369, 10.1172/JCI97354. PMC5983338 29708510

[102] G. T. Tietjen , Z. Gong , C.‐H. Chen , et al., “Molecular Mechanism for Differential Recognition of Membrane Phosphatidylserine by the Immune Regulatory Receptor Tim4,” Proceedings of the National Academy of Sciences of the United States of America 111, no. 15 (2014): E1463–E1472, 10.1073/pnas.1320174111. PMC3992656 24706780

[103] N. Kobayashi , P. Karisola , V. Peña‐Cruz , et al., “TIM‐1 and TIM‐4 Glycoproteins Bind Phosphatidylserine and Mediate Uptake of Apoptotic Cells,” Immunity 27, no. 6 (2007): 927–940, 10.1016/j.immuni.2007.11.011. PMC2757006 18082433

[104] H. Tan , M. Cai , J. Wang , et al., “Harnessing Macrophages in Cancer Therapy: From Immune Modulators to Therapeutic Targets,” International Journal of Biological Sciences 21, no. 5 (2025): 2235–2257, 10.7150/ijbs.106275. PMC11900799 40083710

[105] T. B. Thornley , Z. Fang , S. Balasubramanian , et al., “Fragile TIM‐4–Expressing Tissue Resident Macrophages Are Migratory and Immunoregulatory,” Journal of Clinical Investigation 124, no. 8 (2014): 3443–3454, 10.1172/JCI73527. PMC4109530 24983317

[106] H. Xia , S. Li , X. Li , et al., “Autophagic Adaptation to Oxidative Stress Alters Peritoneal Residential Macrophage Survival and Ovarian Cancer Metastasis,” JCI Insight 5, no. 18 (2020): e141115, 10.1172/jci.insight.141115. PMC7526547 32780724

[107] L. Ding , Y. Liang , Y. Wang , et al., “T‐Cell Immunoglobulin‐ and Mucin‐Domain‐Containing Molecule‐4 Maintains Adipose Tissue Homeostasis by Orchestrating M2 Macrophage Polarization via Nuclear Factor Kappa B Pathway,” Immunology 168, no. 1 (2023): 49–62, 10.1111/imm.13555. 35908188

[108] L. Xu , P. Zhao , Y. Xu , et al., “Tim‐4 Protects Mice Against Lipopolysaccharide‐Induced Endotoxic Shock by Suppressing the NF‐κB Signaling Pathway,” Laboratory Investigation 96, no. 11 (2016): 1189–1197, 10.1038/labinvest.2016.94. 27617399

[109] Z. Pan , E. K. Dumas , C. Lawrence , et al., “Bacillus Anthracis Edema Toxin Inhibits Efferocytosis in Human Macrophages and Alters Efferocytic Receptor Signaling,” International Journal of Molecular Sciences 20, no. 5 (2019): 1167, 10.3390/ijms20051167. PMC6429438 30866434

[110] Y. Cheng , X. Lin , H. Xu , et al., “Integrin β5, a Noninvasive Diagnostic Biomarker, Is Associated With Unfavorable Prognosis and Immunotherapy Efficacy in Gastric Cancer,” BMC Gastroenterology 24, no. 1 (2024): 362, 10.1186/s12876-024-03447-w. PMC11470723 39394072

[111] Y. Zhang , X. Shi , J. Han , et al., “Convallatoxin Promotes M2 Macrophage Polarization to Attenuate Atherosclerosis Through PPARγ‐Integrin αvβ5 Signaling Pathway,” Drug Design, Development and Therapy 15 (2021): 803–812, 10.2147/DDDT.S288728. PMC7914072 33654384

[112] F. N. Soki , A. J. Koh , J. D. Jones , et al., “Polarization of Prostate Cancer‐Associated Macrophages Is Induced by Milk Fat Globule‐EGF Factor 8 (MFG‐E8)‐Mediated Efferocytosis,” Journal of Biological Chemistry 289, no. 35 (2014): 24560–24572, 10.1074/jbc.M114.571620. PMC4148880 25006249

[113] P. Peng , H. Zhu , D. Liu , et al., “TGFBI Secreted by Tumor‐Associated Macrophages Promotes Glioblastoma Stem Cell‐Driven Tumor Growth via Integrin αvβ5‐Src‐Stat3 Signaling,” Theranostics 12, no. 9 (2022): 4221–4236, 10.7150/thno.69605. PMC9169371 35673564

[114] J. Ren , B. Xu , J. Ren , et al., “The Importance of M1‐and M2‐Polarized Macrophages in Glioma and as Potential Treatment Targets,” Brain Sciences 13, no. 9 (2023): 1269, 10.3390/brainsci13091269. PMC10526262 37759870

[115] L. L. Swystun , J. D. Lai , C. Notley , et al., “The Endothelial Cell Receptor Stabilin‐2 Regulates VWF‐FVIII Complex Half‐Life and Immunogenicity,” Journal of Clinical Investigation 128, no. 9 (2018): 4057–4073, 10.1172/JCI96400. PMC6118640 30124466

[116] Z. Szondy , Z. Sarang , B. Kiss , É. Garabuczi , and K. Köröskényi , “Anti‐Inflammatory Mechanisms Triggered by Apoptotic Cells During Their Clearance,” Frontiers in Immunology 8 (2017): 909, 10.3389/fimmu.2017.00909. PMC5539239 28824635

[117] S.‐Y. Park , M.‐Y. Jung , H.‐J. Kim , et al., “Rapid Cell Corpse Clearance by Stabilin‐2, a Membrane Phosphatidylserine Receptor,” Cell Death & Differentiation 15, no. 1 (2008): 192–201, 10.1038/sj.cdd.4402242. 17962816

[118] H. Jo and H. Kim , “Stabilin‐2 Mediated Apoptotic Cell Phagocytosis Induces Interleukin‐10 Expression by p38 and Pbx1 Signaling,” Cell Biochemistry and Biophysics 82, no. 2 (2024): 919–925, 10.1007/s12013-024-01243-7. PMC11344723 38480573

[119] A. Mantovani , S. Sozzani , M. Locati , P. Allavena , and A. Sica , “Macrophage Polarization: Tumor‐Associated Macrophages as a Paradigm for Polarized M2 Mononuclear Phagocytes,” Trends in Immunology 23, no. 11 (2002): 549–555, 10.1016/S1471-4906(02)02302-5. 12401408

[120] J. Ansari , G. Kaur , and F. Gavins , “Therapeutic Potential of Annexin A1 in Ischemia Reperfusion Injury,” International Journal of Molecular Sciences 19, no. 4 (2018): 1211, 10.3390/ijms19041211. PMC5979321 29659553

[121] B. Zhang , Y. Zou , Z. Yuan , et al., “Efferocytosis: The Resolution of Inflammation in Cardiovascular and Cerebrovascular Disease,” Frontiers in Immunology 15 (2024): 1485222, 10.3389/fimmu.2024.1485222. PMC11628373 39660125

[122] B. Cioni , S. Ratti , A. Piva , et al., “JMJD6 Shapes a Pro‐Tumor Microenvironment via ANXA1‐Dependent Macrophage Polarization in Breast Cancer,” Molecular Cancer Research 21, no. 6 (2023): 614–627, 10.1158/1541-7786.MCR-22-0370. PMC10233359 36867680

[123] Z. Qian , W. Fan , F. Meng , et al., “Molecular Characterization and Clinical Relevance of ANXA1 in Gliomas via 1,018 Chinese Cohort Patients,” Frontiers in Cell and Developmental Biology 9 (2021): 777182, 10.3389/fcell.2021.777182. PMC8667664 34912807

[124] L. A. Moraes , S. Kar , S. L. Foo , et al., “Annexin‐A1 Enhances Breast Cancer Growth and Migration by Promoting Alternative Macrophage Polarization in the Tumour Microenvironment,” Scientific Reports 7, no. 1 (2017): 17925, 10.1038/s41598-017-17622-5. PMC5738423 29263330

[125] N. Novizio , R. Belvedere , E. Pessolano , et al., “ANXA1 Contained in EVs Regulates Macrophage Polarization in Tumor Microenvironment and Promotes Pancreatic Cancer Progression and Metastasis,” International Journal of Molecular Sciences 22, no. 20 (2021): 11018, 10.3390/ijms222011018. PMC8538745 34681678

[126] S. Mcarthur , G. Juban , T. Gobbetti , et al., “Annexin A1 Drives Macrophage Skewing to Accelerate Muscle Regeneration Through AMPK Activation,” Journal of Clinical Investigation 130, no. 3 (2020): 1156–1167, 10.1172/JCI124635. PMC7269594 32015229

[127] Z. Song , X. Wang , X. Liu , et al., “Targeting of Annexin A1 in Tumor‐Associated Macrophages as a Therapeutic Strategy for Hepatocellular Carcinoma,” Biochemical Pharmacology 213 (2023): 115612, 10.1016/j.bcp.2023.115612. 37209858

[128] R. G. Da , R. A. Loiola , L. Pantaleao , et al., “Control of Expression and Activity of Peroxisome Proliferated‐Activated Receptor Gamma by Annexin A1 on Microglia During Efferocytosis,” Cell Biochemistry and Function 37, no. 7 (2019): 560–568. 10.1002/cbf.3433 31479167

[129] P. Yang , H. Qin , Y. Li , et al., “CD36‐Mediated Metabolic Crosstalk Between Tumor Cells and Macrophages Affects Liver Metastasis,” Nature Communications 13, no. 1 (2022): 5782, 10.1038/s41467-022-33349-y. PMC9527239 36184646

[130] Z. Hou , F. Lu , J. Lin , et al., “Loss of Annexin A1 in Macrophages Restrains Efferocytosis and Remodels Immune Microenvironment in Pancreatic Cancer by Activating the cGAS/STING Pathway,” Journal for ImmunoTherapy of Cancer 12, no. 9 (2024): e009318, 10.1136/jitc-2024-009318. PMC11381726 39237260

[131] R. Luo , J. Liu , T. Wang , et al., “The Landscape of Malignant Transition: Unraveling Cancer Cell‐of‐Origin and Heterogeneous Tissue Microenvironment,” Cancer Letters 621 (2025): 217591, 10.1016/j.canlet.2025.217591. PMC12040592 40054660

[132] S. Basu , R. J. Binder , T. Ramalingam , and P. K. Srivastava , “CD91 is a Common Receptor for Heat Shock Proteins gp96, hsp90, hsp70, and Calreticulin,” Immunity 14, no. 3 (2001): 303–313, 10.1016/S1074-7613(01)00111-X. 11290339

[133] R. W. Vandivier , C. A. Ogden , V. A. Fadok , et al., “Role of Surfactant Proteins A, D, and C1q in the Clearance of Apoptotic Cells in Vivo and in Vitro: Calreticulin and CD91 as a Common Collectin Receptor Complex,” The Journal of Immunology 169, no. 7 (2002): 3978–3986, 10.4049/jimmunol.169.7.3978. 12244199

[134] X. Liu , B. Yan , Y. Li , et al., “Graphene Oxide‐Grafted Magnetic Nanorings Mediated Magnetothermodynamic Therapy Favoring Reactive Oxygen Species‐Related Immune Response for Enhanced Antitumor Efficacy,” ACS Nano 14, no. 2 (2020): 1936–1950, 10.1021/acsnano.9b08320. 31961656

[135] C.‐C. Duo , F.‐Y. Gong , X.‐Y. He , et al., “Soluble Calreticulin Induces Tumor Necrosis Factor‐α (TNF‐α) and Interleukin (IL)‐6 Production by Macrophages Through Mitogen‐Activated Protein Kinase (MAPK) and NFκB Signaling Pathways,” International Journal of Molecular Sciences 15, no. 2 (2014): 2916–2928, 10.3390/ijms15022916. PMC3958890 24566135

[136] R. Osman , P. Tacnet‐Delorme , J. P. Kleman , A. Millet , and P. Frachet , “Calreticulin Release at an Early Stage of Death Modulates the Clearance by Macrophages of Apoptotic Cells,” Frontiers in Immunology 8 (2017): 1034, 10.3389/fimmu.2017.01034. PMC5572343 28878781

[137] M. Xiao , J. Zhang , W. Chen , and W. Chen , “M1‐Like Tumor‐Associated Macrophages Activated by Exosome‐Transferred THBS1 Promote Malignant Migration in Oral Squamous Cell Carcinoma,” Journal of Experimental & Clinical Cancer Research 37, no. 1 (2018): 143, 10.1186/s13046-018-0815-2. PMC6038304 29986759

[138] Y. Li , X. Qi , X. Tong , and S. Wang , “Thrombospondin 1 Activates the Macrophage Toll‐Like Receptor 4 Pathway,” Cellular & Molecular Immunology 10, no. 6 (2013): 506–512, 10.1038/cmi.2013.32. PMC3818315 23954950

[139] Y. Zhao , Z. Xiong , E. J. Lechner , et al., “Thrombospondin‐1 Triggers Macrophage IL‐10 Production and Promotes Resolution of Experimental Lung Injury,” Mucosal Immunology 7, no. 2 (2014): 440–448, 10.1038/mi.2013.63. PMC3945733 24045574

[140] E. V. Stein , T. W. Miller , K. Ivins‐O'Keefe , S. Kaur , and D. D. Roberts , “Secreted Thrombospondin‐1 Regulates Macrophage Interleukin‐1β Production and Activation Through CD47,” Scientific Reports 6 (2016): 19684, 10.1038/srep19684. PMC4728557 26813769

[141] A. C. Doran , A. J. Yurdagul , and I. Tabas , “Efferocytosis in Health and Disease,” Nature Reviews Immunology 20, no. 4 (2020): 254–267, 10.1038/s41577-019-0240-6. PMC7667664 31822793

[142] A. Trzeciak , Y. T. Wang , and J. Perry , “First We Eat, Then We Do Everything Else: The Dynamic Metabolic Regulation of Efferocytosis,” Cell Metabolism 33, no. 11 (2021): 2126–2141, 10.1016/j.cmet.2021.08.001. PMC8568659 34433074

[143] L. A. J. O'Neill and E. J. Pearce , “Immunometabolism Governs Dendritic Cell and Macrophage Function,” Journal of Experimental Medicine 213, no. 1 (2016): 15–23, 10.1084/jem.20151570. PMC4710204 26694970

[144] L. A. J. O'Neill , R. J. Kishton , and J. Rathmell , “A Guide to Immunometabolism for Immunologists,” Nature Reviews Immunology 16, no. 9 (2016): 553–565, 10.1038/nri.2016.70. PMC5001910 27396447

[145] P. C. Rodriguez , A. H. Zea , K. S. Culotta , J. Zabaleta , J. B. Ochoa , and A. C. Ochoa , “Regulation of T Cell Receptor CD3ζ Chain Expression Byl‐Arginine,” Journal of Biological Chemistry 277, no. 24 (2002): 21123–21129, 10.1074/jbc.M110675200. 11950832

[146] V. Bronte , P. Serafini , A. Mazzoni , D. M. Segal , and P. Zanovello , “L‐Arginine Metabolism in Myeloid Cells Controls T‐Lymphocyte Functions,” Trends in Immunology 24, no. 6 (2003): 301–305, 10.1016/S1471-4906(03)00132-7. 12810105

[147] I. Vitale , G. Manic , L. M. Coussens , G. Kroemer , and L. Galluzzi , “Macrophages and Metabolism in the Tumor Microenvironment,” Cell Metabolism 30, no. 1 (2019): 36–50, 10.1016/j.cmet.2019.06.001. 31269428

[148] R. E. Menjivar , Z. C. Nwosu , W. Du , et al., “Arginase 1 Is a Key Driver of Immune Suppression in Pancreatic Cancer,” Elife 12 (2023): e80721, 10.7554/eLife.80721. PMC10260021 36727849

[149] Y. Zhu , Z. Zhou , X. Du , et al., “Cancer Cell‐Derived Arginine Fuels Polyamine Biosynthesis in Tumor‐Associated Macrophages to Promote Immune Evasion,” Cancer Cell 43, no. 6 (2025): 1045–1060.e7, 10.1016/j.ccell.2025.03.015. 40185095

[150] K. Singh , L. A. Coburn , M. Asim , et al., “Ornithine Decarboxylase in Macrophages Exacerbates Colitis and Promotes Colitis‐Associated Colon Carcinogenesis by Impairing M1 Immune Responses,” Cancer Research 78, no. 15 (2018): 4303–4315, 10.1158/0008-5472.CAN-18-0116. PMC6072585 29853605

[151] W. Li , F. Ying , X. Pang , et al., “Efferocytosis‐Driven Polyamine Metabolism in Macrophages Enhances Cancer Stem Cell Enrichment After Chemotherapy in Ovarian Cancer,” Advanced Science (Weinheim, Baden‐Wurttemberg, Germany) 13, no. 8 (2026): e12508. 10.1002/advs.202512508 PMC12884752 41271556

[152] Q. Ou , S. Tang , J. Zhu , et al., “Spermidine Ameliorates Osteoarthritis via Altering Macrophage Polarization,” Biochimica et Biophysica Acta (BBA)—Molecular Basis of Disease 1870, no. 4 (2024): 167083, 10.1016/j.bbadis.2024.167083. 38367900

[153] D. J. Puleston , M. D. Buck , R. I. Klein Geltink , et al., “Polyamines and eIF5A Hypusination Modulate Mitochondrial Respiration and Macrophage Activation,” Cell Metabolism 30, no. 2 (2019): 352–363.e8, 10.1016/j.cmet.2019.05.003. PMC6688828 31130465

[154] J. Wang , S. Deng , D. Cheng , et al., “Engineered Microparticles Modulate Arginine Metabolism to Repolarize Tumor‐Associated Macrophages for Refractory Colorectal Cancer Treatment,” Journal of Translational Medicine 22, no. 1 (2024): 908, 10.1186/s12967-024-05652-3. PMC11457421 39375706

[155] J. Zhang , S. Wang , X. Guo , et al., “Arginine Supplementation Targeting Tumor‐Killing Immune Cells Reconstructs the Tumor Microenvironment and Enhances the Antitumor Immune Response,” ACS Nano 16, no. 8 (2022): 12964–12978, 10.1021/acsnano.2c05408. 35968927

[156] N. Hajji , J. Garcia‐Revilla , M. S. Soto , et al., “Arginine Deprivation Alters Microglial Polarity and Synergizes With Radiation to Eradicate Non‐Arginine‐Auxotrophic Glioblastoma Tumors,” Journal of Clinical Investigation 132, no. 6 (2022): e142137, 10.1172/JCI142137. PMC8920336 35113813

[157] S. R. Sukka , P. B. Ampomah , L. N. F. Darville , et al., “Efferocytosis Drives a Tryptophan Metabolism Pathway in Macrophages to Promote Tissue Resolution,” Nature Metabolism 6, no. 9 (2024): 1736–1755, 10.1038/s42255-024-01115-7. PMC11734744 39242914

[158] A. Fiore and P. J. Murray , “Tryptophan and Indole Metabolism in Immune Regulation,” Current Opinion in Immunology 70 (2021): 7–14, 10.1016/j.coi.2020.12.001. 33418116

[159] M. de Las Casas‐Engel , A. Domínguez‐Soto , E. Sierra‐Filardi , et al., “Serotonin Skews Human Macrophage Polarization Through HTR2B and HTR7,” Journal of Immunology (Baltimore, Md: 1950) 190, no. 5 (2013): 2301–2310. 10.4049/jimmunol.1201133 23355731

[160] F. Fall , E. Lamy , M. Brollo , et al., “Metabolic Reprograming of LPS‐Stimulated Human Lung Macrophages Involves Tryptophan Metabolism and the Aspartate‐Arginosuccinate Shunt,” PLoS ONE 15, no. 4 (2020): e0230813, 10.1371/journal.pone.0230813. PMC7141605 32267860

[161] A.‐K. Struckmeier , A. Radermacher , M. Fehrenz , et al., “IDO1 is Highly Expressed in Macrophages of Patients in Advanced Tumour Stages of Oral Squamous Cell Carcinoma,” Journal of Cancer Research and Clinical Oncology 149, no. 7 (2023): 3623–3635, 10.1007/s00432-022-04277-7. PMC10314853 35963900

[162] M. Toulmonde , N. Penel , J. Adam , et al., “Use of PD‐1 Targeting, Macrophage Infiltration, and IDO Pathway Activation in Sarcomas,” JAMA Oncology 4, no. 1 (2018): 93, 10.1001/jamaoncol.2017.1617. PMC5833654 28662235

[163] Z. Wang , S. Wang , Z. Jia , et al., “YKL‐40 Derived From Infiltrating Macrophages Cooperates With GDF15 to Establish an Immune Suppressive Microenvironment in Gallbladder Cancer,” Cancer Letters 563 (2023): 216184, 10.1016/j.canlet.2023.216184. 37088328

[164] X. Wen , J. Fu , X. Zhang , et al., “Achieving Immune Activation by Suppressing the IDO1 Checkpoint With Sono‐Targeted Biobromination for Antitumor Combination Immunotherapy,” Journal of the American Chemical Society 146, no. 35 (2024): 24580–24590, 10.1021/jacs.4c07993. 39165059

[165] B. Ravishankar , H. Liu , R. Shinde , et al., “Tolerance to Apoptotic Cells Is Regulated by Indoleamine 2,3‐Dioxygenase,” Proceedings of the National Academy of Sciences of the United States of America 109, no. 10 (2012): 3909–3914, 10.1073/pnas.1117736109. PMC3309765 22355111

[166] J. Xu , C. Weng , Y. Zhang , et al., “GPX4 Knockdown Suppresses M2 Macrophage Polarization in Gastric Cancer by Modulating Kynurenine Metabolism,” Theranostics 15, no. 12 (2025): 5826–5845, 10.7150/thno.108817. PMC12068284 40365295

[167] P. Kesarwani , S. Kant , Y. Zhao , et al., “Quinolinate Promotes Macrophage‐Induced Immune Tolerance in Glioblastoma Through the NMDAR/PPARγ Signaling Axis,” Nature Communications 14, no. 1 (2023): 1459, 10.1038/s41467-023-37170-z. PMC10020159 36927729

[168] Y. Zhao , J. Sun , Y. Li , et al., “Tryptophan 2,3‐Dioxygenase 2 Controls M2 Macrophages Polarization to Promote Esophageal Squamous Cell Carcinoma Progression via AKT/GSK3β/IL‐8 Signaling Pathway,” Acta Pharmaceutica Sinica B 11, no. 9 (2021): 2835–2849, 10.1016/j.apsb.2021.03.009. PMC8463272 34589400

[169] Z. Dong , H. Dai , X. Zhang , et al., “The IDO1/AhR‐HIF‐1α Metabolic Axis: ARNT Competition as a Central Antagonistic Switch in Autoimmune Pathogenesis,” Frontiers in Immunology 17 (2026): 1772536, 10.3389/fimmu.2026.1772536. PMC13002855 41869303

[170] L. Xue , C. Wang , Y. Qian , et al., “Tryptophan Metabolism Regulates Inflammatory Macrophage Polarization as a Predictive Factor for Breast Cancer Immunotherapy,” International Immunopharmacology 125, no. Pt B (2023): 111196, 10.1016/j.intimp.2023.111196. 37972471

[171] Y. F. Jiang , Y. Q. Huang , H. Z. Lai , et al., “Study on the Effect and Mechanism of Banxia Xiexin Decoction on Remodeling Tryptophan Metabolism and Inhibiting the Progression of Colon Cancer,” Zhongguo Zhong Yao Za Zhi = Zhongguo Zhongyao Zazhi = China Journal of Chinese Materia Medica 50, no. 05 (2025): 1310–1320. 10.19540/j.cnki.cjcmm.20241014.702 40350914

[172] D. T. Nguyen , E. K. Kleczko , N. Dwivedi , et al., “The Tryptophan‐Metabolizing Enzyme Indoleamine 2,3‐Dioxygenase 1 Regulates Polycystic Kidney Disease Progression,” JCI Insight 8, no. 1 (2023): e154773, 10.1172/jci.insight.154773. PMC9870090 36422996

[173] S. Morioka , J. S. A. Perry , M. H. Raymond , et al., “Efferocytosis Induces a Novel SLC Program to Promote Glucose Uptake and Lactate Release,” Nature 563, no. 7733 (2018): 714–718, 10.1038/s41586-018-0735-5. PMC6331005 30464343

[174] T. Huang , X. Zhou , X. Mao , et al., “Lactate‐Fueled Oxidative Metabolism Drives DNA Methyltransferase 1‐Mediated Transcriptional co‐Activator With PDZ Binding Domain Protein Activation,” Cancer Science 111, no. 1 (2020): 186–199, 10.1111/cas.14246. PMC6942427 31746077

[175] W. Zhang , Q. Chen , Y. Li , et al., “Integrated Bulk and Single‐Cell Transcriptomics to Develop an Efferocytosis‐Related Prognostic Model for Lung Adenocarcinoma and Validate the Key Gene LDHA,” European Journal of Medical Research 31, no. 1 (2025): 49, 10.1186/s40001-025-03622-z. PMC12798036 41345972

[176] D. Chen , P. Liu , X. Lu , et al., “Pan‐Cancer Analysis Implicates Novel Insights of Lactate Metabolism Into Immunotherapy Response Prediction and Survival Prognostication,” Journal of Experimental & Clinical Cancer Research 43, no. 1 (2024): 125, 10.1186/s13046-024-03042-7. PMC11044366 38664705

[177] D. Zhang , Z. Tang , H. Huang , et al., “Metabolic Regulation of Gene Expression by Histone Lactylation,” Nature 574, no. 7779 (2019): 575–580, 10.1038/s41586-019-1678-1. PMC6818755 31645732

[178] X. Mu , W. Shi , Y. Xu , et al., “Tumor‐Derived Lactate Induces M2 Macrophage Polarization via the Activation of the ERK/STAT3 Signaling Pathway in Breast Cancer,” Cell Cycle 17, no. 4 (2018): 428–438, 10.1080/15384101.2018.1444305. PMC5927648 29468929

[179] C. Yan , Z. Yang , P. Chen , et al., “GPR65 Sensing Tumor‐Derived Lactate Induces HMGB1 Release From TAM via the cAMP/PKA/CREB Pathway to Promote Glioma Progression,” Journal of Experimental & Clinical Cancer Research 43, no. 1 (2024): 105, 10.1186/s13046-024-03025-8. PMC10993467 38576043

[180] P. Chen , H. Zuo , H. Xiong , et al., “Gpr132 Sensing of Lactate Mediates Tumor–Macrophage Interplay to Promote Breast Cancer Metastasis,” Proceedings of the National Academy of Sciences 114, no. 3 (2017): 580–585, 10.1073/pnas.1614035114. PMC5255630 28049847

[181] G. Chen , L. Lin , Z. Mai , et al., “Carrier‐Free Photodynamic Bioregulators Inhibiting Lactic Acid Efflux Combined with Immune Checkpoint Blockade for Triple‐Negative Breast Cancer Immunotherapy,” ACS Nano, published online July 21, 2024, 10.1021/acsnano.4c07213. 39034461

[182] J. Cai , L. Song , F. Zhang , et al., “Targeting SRSF10 Might Inhibit M2 Macrophage Polarization and Potentiate Anti‐PD‐1 Therapy in Hepatocellular Carcinoma,” Cancer Communications 44, no. 11 (2024): 1231–1260, 10.1002/cac2.12607. PMC11570766 39223929

[183] J. Cai , P. Zhang , Y. Cai , et al., “Lactylation‐Driven NUPR1 Promotes Immunosuppression of Tumor‐Infiltrating Macrophages in Hepatocellular Carcinoma,” Advanced Science 12, no. 20 (2025): e2413095, 10.1002/advs.202413095. PMC12120759 40305758

[184] O. R. Colegio , N.‐Q. Chu , A. L. Szabo , et al., “Functional Polarization of Tumour‐Associated Macrophages by Tumour‐Derived Lactic Acid,” Nature 513, no. 7519 (2014): 559–563, 10.1038/nature13490. PMC4301845 25043024

[185] Z. X. Liao , Y. C. Fa , I. M. Kempson , and S. J. Tseng , “Repolarization of M2 to M1 Macrophages Triggered by Lactate Oxidase Released From Methylcellulose Hydrogel,” Bioconjugate Chemistry 30, no. 10 (2019): 2697–2702, 10.1021/acs.bioconjchem.9b00618. 31532192

[186] S. Tan , Z. Wang , N. Li , et al., “Transcription Factor Zhx2 Is a Checkpoint That Programs Macrophage Polarization and Antitumor Response,” Cell Death & Differentiation 30, no. 9 (2023): 2104–2119, 10.1038/s41418-023-01202-4. PMC10482862 37582865

[187] K. Yang , J. Xu , M. Fan , et al., “Lactate Suppresses Macrophage Pro‐Inflammatory Response to LPS Stimulation by Inhibition of YAP and NF‐κB Activation via GPR81‐Mediated Signaling,” Frontiers in Immunology 11 (2020): 587913, 10.3389/fimmu.2020.587913. PMC7573489 33123172

[188] S. Zhang , S. Weinberg , M. Deberge , et al., “Efferocytosis Fuels Requirements of Fatty Acid Oxidation and the Electron Transport Chain to Polarize Macrophages for Tissue Repair,” Cell Metabolism 29, no. 2 (2019): 443–456.e5, 10.1016/j.cmet.2018.12.004. PMC6471613 30595481

[189] J. Liu , W. Zhang , L. Chen , et al., “VSIG4 Promotes Tumour‐Associated Macrophage M2 Polarization and Immune Escape in Colorectal Cancer via Fatty Acid Oxidation Pathway,” Clinical and Translational Medicine 15, no. 5 (2025): e70340, 10.1002/ctm2.70340. PMC12098961 40405491

[190] R. Zhu , J. Na , X. Tan , et al., “Mechanistic Research on the Crosstalk Between Macrophage Polarization and Energy Metabolic Reprogramming in Hepatocellular Carcinoma,” Journal of Hepatocellular Carcinoma 13 (2026): 1–24, 10.2147/JHC.S583017. PMC12973307 41816775

[191] M. M. Albakri , S. C. Huang , H. N. Tashkandi , and S. F. Sieg , “Fatty Acids Secreted From Head and Neck Cancer Induce M2‐Like Macrophages,” Journal of Leukocyte Biology 112, no. 4 (2022): 617–628, 10.1002/JLB.1A0521-251R. 35213745

[192] T. Liu , X. Zhang , X. Yan , et al., “Smad4 Deficiency in S100A4 + Macrophages Enhances Colitis‐Associated Tumorigenesis by Promoting Macrophage Lipid Metabolism Augmented M2 Polarization,” International Journal of Biological Sciences 20, no. 15 (2024): 6114–6129, 10.7150/ijbs.98529. PMC11628331 39664586

[193] Q. Luo , N. Zheng , L. Jiang , et al., “Lipid Accumulation in Macrophages Confers Protumorigenic Polarization and Immunity in Gastric Cancer,” Cancer Science 111, no. 11 (2020): 4000–4011, 10.1111/cas.14616. PMC7648032 32798273

[194] A. Chawla , “Control of Macrophage Activation and Function by PPARs,” Circulation Research 106, no. 10 (2010): 1559–1569, 10.1161/CIRCRESAHA.110.216523. PMC2897247 20508200

[195] G. H. O. Da Rocha , R. A. Loiola , L. D. N. Pantale , et al., “Control of Expression and Activity of Peroxisome Proliferated‐Activated Receptor γ by Annexin A1 on Microglia During Efferocytosis,” Cell Biochemistry and Function 37, no. 7 (2019): 560–568. 10.1002/cbf.3433 31479167

[196] S. Liu , H. Zhang , Y. Li , et al., “S100A4 Enhances Protumor Macrophage Polarization by Control of PPAR‐γ‐Dependent Induction of Fatty Acid Oxidation,” Journal for ImmunoTherapy of Cancer 9, no. 6 (2021): e002548, 10.1136/jitc-2021-002548. PMC8215236 34145030

[197] H.‐M. Wu , X.‐X. Ni , Q.‐Y. Xu , Q. Wang , X.‐Y. Li , and J. Hua , “Regulation of Lipid‐Induced Macrophage Polarization Through Modulating Peroxisome Proliferator‐Activated Receptor‐Gamma Activity Affects Hepatic Lipid Metabolism via a Toll‐like Receptor 4/NF‐κB Signaling Pathway,” Journal of Gastroenterology and Hepatology 35, no. 11 (2020): 1998–2008, 10.1111/jgh.15025. 32128893

[198] J. Huang , H. Pan , J. Sun , et al., “TMEM147 Aggravates the Progression of HCC by Modulating Cholesterol Homeostasis, Suppressing Ferroptosis, and Promoting the M2 Polarization of Tumor‐Associated Macrophages,” Journal of Experimental & Clinical Cancer Research 42, no. 1 (2023): 286, 10.1186/s13046-023-02865-0. PMC10612308 37891677

[199] S. C.‐C. Huang , B. Everts , Y. Ivanova , et al., “Cell‐Intrinsic Lysosomal Lipolysis Is Essential for Alternative Activation of Macrophages,” Nature Immunology 15, no. 9 (2014): 846–855, 10.1038/ni.2956. PMC4139419 25086775

[200] H. Qin , A. Xiao , Q. Lu , et al., “The Fatty Acid Receptor CD36 Promotes Macrophage Infiltration via p110γ Signaling to Stimulate Metastasis,” Journal of Advanced Research 74 (2025): 237–253, 10.1016/j.jare.2024.10.006. PMC12302359 39419288

[201] L. Xu , N. Nagata , M. Nagashimada , et al., “SGLT2 Inhibition by Empagliflozin Promotes Fat Utilization and Browning and Attenuates Inflammation and Insulin Resistance by Polarizing M2 Macrophages in Diet‐Induced Obese Mice,” EBioMedicine 20 (2017): 137–149, 10.1016/j.ebiom.2017.05.028. PMC5478253 28579299

[202] M. An , A. Mehta , B. H. Min , et al., “Early Immune Remodeling Steers Clinical Response to First‐Line Chemoimmunotherapy in Advanced Gastric Cancer,” Cancer Discovery 14, no. 5 (2024): 766–785, 10.1158/2159-8290.CD-23-0857. PMC11061611 38319303

[203] M. Larroquette , J.‐P. Guegan , B. Besse , et al., “Spatial Transcriptomics of Macrophage Infiltration in Non‐Small Cell Lung Cancer Reveals Determinants of Sensitivity and Resistance to Anti‐PD1/PD‐L1 Antibodies,” Journal for ImmunoTherapy of Cancer 10, no. 5 (2022): e003890, 10.1136/jitc-2021-003890. PMC9125754 35618288

[204] N. Liu , J. Zhang , M. Yin , et al., “Inhibition of xCT Suppresses the Efficacy of Anti‐PD‐1/L1 Melanoma Treatment Through Exosomal PD‐L1‐Induced Macrophage M2 Polarization,” Molecular Therapy 29, no. 7 (2021): 2321–2334, 10.1016/j.ymthe.2021.03.013. PMC8261162 33744468

[205] R. Noy and J. W. Pollard , “Tumor‐Associated Macrophages: From Mechanisms to Therapy,” Immunity 41, no. 1 (2014): 49–61, 10.1016/j.immuni.2014.06.010. PMC4137410 25035953

[206] D. O. Adeegbe and H. Nishikawa , “Natural and Induced T Regulatory Cells in Cancer,” Frontiers in Immunology 4 (2013): 190, 10.3389/fimmu.2013.00190. PMC3708155 23874336

[207] S. A. Oh and M. O. Li , “TGF‐β: Guardian of T Cell Function,” The Journal of Immunology 191, no. 8 (2013): 3973–3979, 10.4049/jimmunol.1301843. PMC3856438 24098055

[208] T. H. S. Ng , G. J. Britton , E. V. Hill , J. Verhagen , B. R. Burton , and D. C. Wraith , “Regulation of Adaptive Immunity; the Role of Interleukin‐10,” Frontiers in Immunology 4 (2013): 129, 10.3389/fimmu.2013.00129. PMC3668291 23755052

[209] T. Krneta , A. Gillgrass , S. Poznanski , et al., “M2‐Polarized and Tumor‐Associated Macrophages Alter NK Cell Phenotype and Function in a Contact‐Dependent Manner,” Journal of Leukocyte Biology 101, no. 1 (2017): 285–295, 10.1189/jlb.3A1215-552R. 27493241

[210] Y. Li , Y. Weng , L. Zhong , et al., “VEGFR3 Inhibition Chemosensitizes Lung Adenocarcinoma A549 Cells in the Tumor‐Associated Macrophage Microenvironment Through Upregulation of p53 and PTEN,” Oncology Reports 38, no. 5 (2017): 2761–2773, 10.3892/or.2017.5969. PMC5780029 29048623

[211] C. Stockmann , A. Doedens , A. Weidemann , et al., “Deletion of Vascular Endothelial Growth Factor in Myeloid Cells Accelerates Tumorigenesis,” Nature 456, no. 7223 (2008): 814–818, 10.1038/nature07445. PMC3103772 18997773

[212] X. Guo , H. Zhang , C. He , et al., “RUNX1 Promotes Angiogenesis in Colorectal Cancer by Regulating the Crosstalk Between Tumor Cells and Tumor Associated Macrophages,” Biomarker Research 12, no. 1 (2024): 29, 10.1186/s40364-024-00573-1. PMC10903076 38419056

[213] E. Fu , T. Liu , S. Yu , et al., “M2 Macrophages Reduce the Radiosensitivity of Head and Neck Cancer by Releasing HB‑EGF,” Oncology Reports 44, no. 2 (2020): 698–710, 10.3892/or.2020.7628. PMC7336513 32627036

[214] D. Ajona , S. Ortiz‐Espinosa , T. Lozano , et al., “Short‐Term Starvation Reduces IGF‐1 Levels to Sensitize Lung Tumors to PD‐1 Immune Checkpoint Blockade,” Nature Cancer 1, no. 1 (2020): 75–85, 10.1038/s43018-019-0007-9. 35121837

[215] K. Fang , M. Sun , Z. Leng , et al., “Targeting IGF1R Signaling Enhances the Sensitivity of Cisplatin by Inhibiting Proline and Arginine Metabolism in Oesophageal Squamous Cell Carcinoma Under Hypoxia,” Journal of Experimental & Clinical Cancer Research 42, no. 1 (2023): 73, 10.1186/s13046-023-02623-2. PMC10044411 36978187

[216] D. D. Karp , M. N. Pollak , R. B. Cohen , et al., “Safety, Pharmacokinetics, and Pharmacodynamics of the Insulin‐Like Growth Factor Type 1 Receptor Inhibitor Figitumumab (CP‐751,871) in Combination With Paclitaxel and Carboplatin,” Journal of Thoracic Oncology 4, no. 11 (2009): 1397–1403, 10.1097/JTO.0b013e3181ba2f1d. PMC2941876 19745765

[217] M. A. Villalona‐Calero , G. A. Otterson , M. G. Wientjes , et al., “Noncytotoxic Suramin as a Chemosensitizer in Patients With Advanced Non‐Small‐Cell Lung Cancer: A Phase II Study,” Annals of Oncology 19, no. 11 (2008): 1903–1909, 10.1093/annonc/mdn412. PMC2733080 18632723

[218] B. L. Rapoport , G. Falkson , J. I. Raats , M. De Wet , B. P. Lotz , and H. C. Potgieter , “Suramin in Combination With Mitomycin C in Hormone‐Resistant Prostate Cancer. A Phase II Clinical Study,” Annals of Oncology 4, no. 7 (1993): 567–573, 10.1093/oxfordjournals.annonc.a058590. 8363988

[219] S. K. Pal , H. A. Drabkin , J. A. Reeves , et al., “A Phase 2 Study of the Sphingosine‐1‐Phosphate Antibody Sonepcizumab in Patients With Metastatic Renal Cell Carcinoma,” Cancer 123, no. 4 (2017): 576–582, 10.1002/cncr.30393. 27727447

[220] E. J. Small , S. Halabi , M. J. Ratain , et al., “Randomized Study of Three Different Doses of Suramin Administered With a Fixed Dosing Schedule in Patients With Advanced Prostate Cancer: Results of Intergroup 0159, Cancer and Leukemia Group B 9480,” Journal of Clinical Oncology 20, no. 16 (2002): 3369–3375, 10.1200/JCO.2002.10.022. 12177096

[221] S. A. Grossman , S. Phuphanich , G. Lesser , et al., “Toxicity, Efficacy, and Pharmacology of Suramin in Adults With Recurrent High‐Grade Gliomas,” Journal of Clinical Oncology 19, no. 13 (2001): 3260–3266, 10.1200/JCO.2001.19.13.3260. 11432894

[222] E. J. Small , M. Meyer , M. E. Marshall , et al., “Suramin Therapy for Patients With Symptomatic Hormone‐Refractory Prostate Cancer: Results of a Randomized Phase III Trial Comparing Suramin Plus Hydrocortisone to Placebo Plus Hydrocortisone,” Journal of Clinical Oncology 18, no. 7 (2000): 1440–1450, 10.1200/JCO.2000.18.7.1440. 10735891

[223] J. Kummetha Venkata , N. An , R. Stuart , et al., “Inhibition of Sphingosine Kinase 2 Downregulates the Expression of c‐Myc and Mcl‐1 and Induces Apoptosis in Multiple Myeloma,” Blood 124, no. 12 (2014): 1915–1925, 10.1182/blood-2014-03-559385. PMC4168346 25122609

[224] C. D. Britten , E. Garrett‐Mayer , S. H. Chin , et al., “A Phase I Study of ABC294640, a First‐in‐Class Sphingosine Kinase‐2 Inhibitor, in Patients With Advanced Solid Tumors,” Clinical Cancer Research 23, no. 16 (2017): 4642–4650, 10.1158/1078-0432.CCR-16-2363. PMC5559328 28420720

[225] Y. Kang , P. Sundaramoorthy , C. Gasparetto , et al., “Phase I Study of Opaganib, an Oral Sphingosine Kinase 2‐Specific Inhibitor, in Relapsed and/or Refractory Multiple Myeloma,” Annals of Hematology 102, no. 2 (2023): 369–383, 10.1007/s00277-022-05056-7. 36460794

[226] D. Hsiehchen , M. S. Beg , R. Kainthla , et al., “The Phosphatidylserine Targeting Antibody Bavituximab Plus Pembrolizumab in Unresectable Hepatocellular Carcinoma: A Phase 2 Trial,” Nature Communications 15, no. 1 (2024): 2178, 10.1038/s41467-024-46542-y. PMC10928173 38467639

[227] K. He , D. Berz , S. M. Gadgeel , et al., “MRTX‐500 Phase 2 Trial: Sitravatinib With Nivolumab in Patients With Nonsquamous NSCLC Progressing on or After Checkpoint Inhibitor Therapy or Chemotherapy,” Journal of Thoracic Oncology 18, no. 7 (2023): 907–921, 10.1016/j.jtho.2023.02.016. PMC10330304 36842467

[228] D. E. Gerber , L. Horn , M. Boyer , et al., “Randomized Phase III Study of Docetaxel Plus Bavituximab in Previously Treated Advanced Non‐Squamous Non‐Small‐Cell Lung Cancer,” Annals of Oncology 29, no. 7 (2018): 1548–1553, 10.1093/annonc/mdy177. PMC6887726 29767677

[229] K. E. Beckermann , N. J. Shah , M. T. Campbell , et al., “Phase 1b/2 Study of Batiraxcept Alone and in Combination With Cabozantinib With or Without Nivolumab for Advanced Clear Cell Renal Cell Carcinoma,” The Oncologist 30, no. 6 (2025): oyaf138, 10.1093/oncolo/oyaf138. PMC12205982 40549043

[230] A. Knisely , E. M. Hinchcliff , E. Gardiner , et al., “Phase 1b Study of Batiraxcept in Combination With Durvalumab in Patients With Platinum‐Resistant Ovarian Cancer,” Iscience 27, no. 5 (2024): 109801, 10.1016/j.isci.2024.109801. PMC11079458 38726365

[231] S. Loges , M. Heuser , J. Chromik , et al., “Bemcentinib as Monotherapy and in Combination With Low‐Dose Cytarabine in Acute Myeloid Leukemia Patients Unfit for Intensive Chemotherapy: A Phase 1b/2a Trial,” Nature Communications 16, no. 1 (2025): 2846, 10.1038/s41467-025-58179-6. PMC11930985 40122885

[232] A. S. Kubasch , P. Peterlin , T. Cluzeau , et al., “Efficacy and Safety of Bemcentinib in Patients With Advanced Myelodysplastic Neoplasms or Acute Myeloid Leukemia Failing Hypomethylating Agents—The EMSCO Phase II BERGAMO Trial,” Leukemia 37, no. 11 (2023): 2309–2313, 10.1038/s41375-023-02029-1. PMC10624604 37735558

[233] P. Mazzeo , S. M. Penir , E. Shumilov , et al., “Clonal Evolution and Apoptosis Resistance in Myelodysplastic Neoplasms and Acute Myeloid Leukemia Under Treatment: Insights From Integrative Longitudinal Profiling,” Leukemia 39, no. 12 (2025): 3026–3036, 10.1038/s41375-025-02756-7. PMC12634422 40973765

[234] M. Park and K. W. Kang , “Phosphatidylserine Receptor‐Targeting Therapies for the Treatment of Cancer,” Archives of Pharmacal Research 42, no. 7 (2019): 617–628, 10.1007/s12272-019-01167-4. 31172440

[235] H. Chen , Y. Yang , Y. Deng , et al., “Delivery of CD47 Blocker SIRPα‐Fc by CAR‐T Cells Enhances Antitumor Efficacy,” Journal for ImmunoTherapy of Cancer 10, no. 2 (2022): e003737, 10.1136/jitc-2021-003737. PMC8811602 35110357

[236] Z.‐H. Wu , N. Li , X.‐F. Mei , et al., “Preclinical Characterization of the Novel Anti‐SIRPα Antibody BR105 That Targets the Myeloid Immune Checkpoint,” Journal for ImmunoTherapy of Cancer 10, no. 3 (2022): e004054, 10.1136/jitc-2021-004054. PMC8905892 35256517

[237] T. C. Kuo , A. Chen , O. Harrabi , et al., “Targeting the Myeloid Checkpoint Receptor SIRPα Potentiates Innate and Adaptive Immune Responses to Promote Anti‐Tumor Activity,” Journal of Hematology & Oncology 13, no. 1 (2020): 160, 10.1186/s13045-020-00989-w. PMC7706287 33256806

[238] R. Advani , I. Flinn , L. Popplewell , et al., “CD47 Blockade by Hu5F9‐G4 and Rituximab in Non‐Hodgkin's Lymphoma,” New England Journal of Medicine 379, no. 18 (2018): 1711–1721, 10.1056/NEJMoa1807315. PMC8058634 30380386

[239] P. Strati , L. Feng , A. Tyshevich , et al., “A Phase I Trial of Evorpacept, Lenalidomide, and Rituximab for Patients With B‐Cell Non‐Hodgkin Lymphoma,” Clinical Cancer Research 31, no. 19 (2025): 4079–4088, 10.1158/1078-0432.CCR-25-1826. PMC12370181 40729376

[240] S. G. Kimani , K. Geng , C. Kasikara , et al., “Contribution of Defective PS Recognition and Efferocytosis to Chronic Inflammation and Autoimmunity,” Frontiers in Immunology 5 (2014): 566, 10.3389/fimmu.2014.00566. PMC4226236 25426118

[241] A. Kosta , A. Mekhloufi , L. Lucantonio , et al., “GAS6/TAM Signaling Pathway Controls MICA Expression in Multiple Myeloma Cells,” Frontiers in Immunology 13 (2022): 942640, 10.3389/fimmu.2022.942640. PMC9368199 35967396

[242] S. M. Post , M. Andreeff , C. DiNardo , J. D. Khoury , and P. P. Ruvolo , “TAM Kinases as Regulators of Cell Death,” Biochimica et Biophysica Acta (BBA)—Molecular Cell Research 1868, no. 6 (2021): 118992, 10.1016/j.bbamcr.2021.118992. 33647320

[243] Q. Lu and G. Lemke , “Homeostatic Regulation of the Immune System by Receptor Tyrosine Kinases of the Tyro 3 Family,” Science 293, no. 5528 (2001): 306–311, 10.1126/science.1061663. 11452127

[244] S. Shiba , K. Ikeda , K. Horie‐Inoue , et al., “Vitamin K‐Dependent γ ‐Glutamyl Carboxylase in Sertoli Cells Is Essential for Male Fertility in Mice,” Molecular and Cellular Biology 41, no. 4 (2021): e00404–e00420, 10.1128/MCB.00404-20. PMC8088131 33526452

[245] I. K. H. Poon and K. S. Ravichandran , “Targeting Efferocytosis in Inflammaging,” Annual Review of Pharmacology and Toxicology 64 (2024): 339–357, 10.1146/annurev-pharmtox-032723-110507. 37585658

[246] A. J. Ridley , “Rho GTPases and Cell Migration,” Journal of Cell Science 114, no. Pt 15 (2001): 2713–2722, 10.1242/jcs.114.15.2713. 11683406

[247] J. D. Rotty , H. E. Brighton , S. L. Craig , et al., “Arp2/3 Complex Is Required for Macrophage Integrin Functions but Is Dispensable for FcR Phagocytosis and in Vivo Motility,” Developmental Cell 42, no. 5 (2017): 498–513.e6, 10.1016/j.devcel.2017.08.003. PMC5601320 28867487

[248] S. Rber , A. Junemann , C. Litschko , M. Winterhoff , and J. Faix , “Convergence of Ras‐ and Rac‐Regulated Formin Pathways Is Pivotal for Phagosome Formation and Particle Uptake in Dictyostelium,” Proceedings of the National Academy of Sciences of the United States of America 120, no. 11 (2023): e2220825120. 10.1073/pnas.2220825120 PMC10243128 36897976

[249] A. Yurdagul , M. Subramanian , X. Wang , et al., “Macrophage Metabolism of Apoptotic Cell‐Derived Arginine Promotes Continual Efferocytosis and Resolution of Injury,” Cell Metabolism 31, no. 3 (2020): 518–533.e10, 10.1016/j.cmet.2020.01.001. PMC7173557 32004476

[250] L. Zhang , Z. Li , K. M. Skrzypczynska , et al., “Single‐Cell Analyses Inform Mechanisms of Myeloid‐Targeted Therapies in Colon Cancer,” Cell 181, no. 2 (2020): 442–459.e29, 10.1016/j.cell.2020.03.048. 32302573

[251] L. Yu , C. Rivas , F. Liu , et al., “Inflammation‐ and Resolution‐Programmed Myeloid Circuits Govern Therapeutic Resistance in Epithelial and Mesenchymal Triple‐Negative Breast Cancer,” Journal of Clinical Investigation 136, no. 8 (2026): e198815, 10.1172/JCI198815. PMC13078875 41701526

[252] Y. Liu , H. Zhao , R. Niu , et al., “Defective Single‐Site Nanozymes With Exposed Unsaturated Cu–N 2 Sites for Antitumor Immunotherapy via Innate Immune‐Checkpoint Blockade,” Journal of the American Chemical Society 148, no. 9 (2026): 9277–9295, 10.1021/jacs.5c13802. 41732963

[253] P. Huang , Y. Liu , C. Zhao , et al., “Permanent Efferocytosis Prevention by Terminating MerTK Recycle on Tumor‐Associated Macrophages for Cancer Immunotherapy,” Journal of the American Chemical Society 147, no. 18 (2025): 15901–15914, 10.1021/jacs.5c05640. 40294287

[254] Y. Ren , Q. Fan , X. Yao , et al., “A Phosphatidylserine‐Targeted Self‐Amplifying Nanosystem Improves Tumor Accumulation and Enables Efficient Tumor Therapy by Modulating Anticancer Immunity,” Biomaterials 324 (2026): 123526, 10.1016/j.biomaterials.2025.123526. 40577987

[255] A. I. Medeiros , C. H. Serezani , S. P. Lee , and M. Peters‐Golden , “Efferocytosis Impairs Pulmonary Macrophage and Lung Antibacterial Function via PGE2/EP2 Signaling,” Journal of Experimental Medicine 206, no. 1 (2009): 61–68, 10.1084/jem.20082058. PMC2626688 19124657

[256] D. Gu , D. Fang , M. Zhang , et al., “Gastrin, via Activation of PPARα, Protects the Kidney Against Hypertensive Injury,” Clinical Science 135, no. 2 (2021): 409–427, 10.1042/CS20201340. PMC8594318 33458737

[257] Z. Lin , X. Zhou , S. Liu , M. Wan , and J. Liu , “Advancements in Nanomedicine for the Therapeutic Regulation of Efferocytosis: Opportunities and Challenges,” Theranostics 16, no. 10 (2026): 5537–5570, 10.7150/thno.128155. PMC13080804 41993634

